# Comparative Analysis of Immune Repertoires between Bactrian Camel's Conventional and Heavy-Chain Antibodies

**DOI:** 10.1371/journal.pone.0161801

**Published:** 2016-09-02

**Authors:** Xinyang Li, Xiaobo Duan, Kai Yang, Wei Zhang, Changjiang Zhang, Longfei Fu, Zhe Ren, Changxi Wang, Jinghua Wu, Ruxue Lu, Yanrui Ye, Mengying He, Chao Nie, Naibo Yang, Jian Wang, Huanming Yang, Xiao Liu, Wen Tan

**Affiliations:** 1 Guangdong Provincial Key Laboratory of Fermentation and Enzyme Engineering, School of Bioscience and Bioengineering, South China University of Technology, Guangzhou 510006, China; 2 BGI-Shenzhen, Shenzhen 518083, China; 3 Department of Biology, University of Copenhagen, Copenhagen 2200, Denmark; 4 Key Laboratory of Industrial Biotechnology of Guangdong Higher Education Institutes, School of Bioscience and Bioengineering, South China University of Technology, Guangzhou 510006, China; 5 Pre-Incubator for Innovative Drugs & Medicine, School of Bioscience and Bioengineering, South China University of Technology, Guangzhou 510006, China; 6 Zhangye City Bureau of Animal Husbandry and Veterinary, Zhangye, Gansu, 734000, China; 7 James D. Watson Institute of Genome Sciences, Hangzhou 310058, China; Chang Gung University, TAIWAN

## Abstract

Compared to classical antibodies, camel heavy chain antibodies (HCAbs) are smaller in size due to lack of the light chain and the first constant domain of the heavy chain (CH1 region). The variable regions of HCAbs (VHHs) are more soluble and stable than that of conventional antibodies (VHs). Even with such simple structure, they are still functional in antigen binding. Although HCAbs have been extensively investigated over the past two decades, most efforts have been based upon low throughput sequence analysis, and there are only limited reports trying to analyze and describe the complete immune repertoire (IR) of camel HCAbs. Here we leveraged the high-throughput data generated by Next Generation Sequencing (NGS) of the variable domains of the antibody heavy chains from three Bactrian camels to conduct in-depth comparative analyses of the immunoglobulin repertoire. These include analyses of the complementary determining region 3 (CDR3) length and distribution, mutation rate, antibody characteristic amino acids, the distribution of the cysteine (Cys) codons, and the non-classical VHHs. We found that there is higher diversity in the CDR2 than in the other sub-regions, and there is a higher mutation rate in the VHHs than in the VHs (*P* < 0.05). In addition to substitutions at amino acid (AA) residue positions NO.49/50/52 between VH and VHH clones, we also observed other substitutions at the positions NO.40/54/57/96/101 that could lead to additional structural alterations. We also found that VH-derived VHH clones, referred to as non-classical VHH clones in this study, accounted for about 8% of all clones. Further, only 5%-10% clones had the Trp > Arg AA substitution at the first position of framework 4 for all types of clones. We present, for the first time, a relatively complete picture of the Bactrian camel antibody immune repertoire, including conventional antibody (Ab) and HCAbs, using PCR and *in silico* analysis based on high-throughput NGS data.

## Introduction

It is generally recognized that all camelids produce, besides classical antibodies, high amounts of heavy-chain antibodies (HCAbs) circulating in their blood. Unlike conventional antibodies, these HCAbs are devoid of light chains and the CH1 region and are composed of only a heavy-chain homodimer. These antibodies are expressed after a variable (V), diversity (D), and joining (J) segments rearrangement and require dedicated constant genes [1].

There is a continual demand in biomedicine for antibodies that recognize target molecules with high affinity and specificity [2]. Nanobodies (Nbs) are single-domain antigen-binding fragments derived from the HCAbs. Nbs have a number of unique advantages that make them highly attractive in various applications. As such, they have emerged as an alternative to conventional antibodies. The variable region of a Camel’s HCAbs (referred as to VHH, conventional antibody heavy chain variable regions are referred to as VH) is one of the smallest antigen-binding single polypeptide chains found in natural antibodies [3–7]. Unlike the variable regions of conventional antibodies, Nbs are extremely stable, can bind antigens with affinities in the nanomolar range, and are smaller in size (approximately 15 kDa) than most other antibody constructs [1, 4, 8–11]. Moreover, they can readily be produced in many recombinant protein expression systems, including bacterial and yeast cells [5, 12].

The immune system’s antibody repertoire is highly plastic and can be directed to create antibodies with broad chemical diversity and high selectivity [13]. In recent years, a powerful new technology based on NGS has been developed to probe the adaptive immune system. Millions of T cell receptors and immunoglobulin sequences from a single sample can be amplified in a single multiplex PCR reaction and analyzed in parallel [14]. For instance, the high sensitivity of this technology has enabled a more reliable estimation of minimal residual disease in various leukemias [15, 16]. In addition, it has been shown that combining immune repertoire sequencing and polyclonal antibody mass spectrometry is an efficient way to obtain sequences of antigen specific antibodies. [2, 17, 18].

Recently, Griffin *et al*. [19] described the sequence diversity of functional variable and constant regions observed in 57 conventional heavy, 18 kappa and 35 lambda light chains of *Camelus dromedarius* and *Camelus bactrianus* based on Sanger sequencing. In addition, Klarenbeek *et al*. [20] constructed the germline repertoire of V genes by using the publicly available High-Throughput Genomic and Whole Genome Shotgun databases of *Lama pacos* and *Camelus ferus*. The aforementioned studies were based on the repertoire of conventional antibody V genes and were based on low throughput sequencing and data mining. To date, there has not been a comprehensive analysis of the camel`s VHH repertoire based on high-throughput sequencing (HTS) of the entire immune repertoire (IR) of *Camelus*. *bactrianus*.

In this study we fill this void by constructing the immune repertoires of Bactrian camel`s clan III family genes. Camel’s VH(3) (Clan III) family is by far the most abundant and representative V family in camelids [21]. We analyzed the characteristics of the immune repertoire between VH and VHH clones and compared them in depth across multiple aspects. The analysis of the camelus IR data presented here will lay the foundation for future studies and biomedical applications of camel antibodies.

## Materials and Methods

### Animals and ethics

Three healthy Alashan domestic Bactrian camels (NO. 1, five years old, female; NO. 2, three years old, male, and NO. 3, three years old, female) were feed freely in Lao Ye’s Farm of Pingshan, Zhangye City, Gansu province of China. The geographic coordinates of this farm is 100°39'51"N, 39°15'54"E. This project has been reviewed and approved by the Bioethics and Biological Safety Review Committee of BGI-Shenzhen (Permit Number is FT 15052). All blood collection was performed under gentle fixation and all efforts were made to minimize suffering.

### Blood sampling and RNA extraction

Bactrian camel peripheral blood mononuclear cells (PBMCs) were separated from peripheral blood by density gradient centrifugation in Percoll. The RNA was extracted from approximately 5 × 10^6^ PBMCs from each camel using an RNEasy kit (Qiagen, Hilden, Germany), according to the manufacturer's protocol, and then stored at −80°C.

### Immune library construction and sequencing

N6 random primers were used to prepare cDNA templates from RNA. To amplify the camel immunoglobulin genes from cDNA, one pair of gene-specific primers CALL001 (5`-GTCCTGGCTGCTCTTCTACAAGG-3`) and CALL002 (5`-GGTACGTGCTGTTGAACTGTTCC-3`) [22] were used in subsequent PCR. The CALL001 and CALL002 primers amplified all V elements of the VH(3) family (by far the most abundant V family in camelids) [23]. The 750-bp and 1,000-bp PCR products were separated by 2% agarose gel electrophoresis and purified using a Qiagen gel-purification kit (Qiagen, Hilden, Germany) following the manufacturer’s protocol. Next, we re-amplified the antibody-encoding V genes (VHs and VHHs) with nested primers VHH-forward (5`-ATGGCTSAKGTGCAGCTGGTGGAGTCTGG-3`) and VHH-reverse (5`-GGAGACGGTGACCTGGGT-3`) [22], annealing at framework 1 and framework 4 of the immunoglobulin variable regions respectively. For Mi-Seq (Illumina, San Diego, CA) sequencing, PCR products from the previous step were tagged with 8 bp barcodes for cluster identification, after which they were ligated to Mi-Seq adaptors for library preparation. Final products were run on a Mi-Seq sequencer to generate 2 ×300-bp paired-end reads.

### Data analysis

Sequence data were analyzed by IMonitor [24], a pipeline we previously developed for analyzing IR data. High-quality paired reads were merged into contigs using an accurate read connection tool called Connecting Overlapped Pair-End (COPE) [25] and another tool called FqMergerc (developed at BGI) [24]. Contigs were aligned to the camelus BCR reference sequences from the international ImMunoGeneTics database (IMGT) and were used for BLAST alignment of V and J germline sequences. It was followed by re-alignment of each result and selection of the best V/J alignment of each contig. Contigs with less than 2 reads corresponding to the CDR3 region were removed and the remaining sequences were translated. Shugay *et al*. [26] demonstrated that errors introduced during PCR and sequencing of the VHH may introduce some false positive clonal variants into antibody sequencing datasets. To avoid this critical issue, we performed PCR and sequencing error correction with “IMonitor” [24]. After the correction process, the mean error rate of all sequences decreased from 0.082% to 0.013%, and the percent of error-bearing sequences decreased significantly from 6.313% to 0.912%. Finally, we performed analyses and generated statistics on CDR3 sequence length and distribution, conservation and diversity, nucleotide mutations, AA characteristics, the distribution of cysteine (Cys) codons, and non-classical VHH clones. All NGS sequence data has been uploaded into the Sequence Read Archive (SRA) repository of the NCBI under accession number PRJNA321369.

### Statistical analysis

All the statistical analyses were performed with the software IMonitor [24]. Comparisons between groups were performed using two-tailed t-tests with two-sided *P* values smaller than 0.05 considered statistically significant. The *P* values were corrected for multiple testing using the Benjamini-Hochberg method.

## Results

### CDR3 length and distribution

**Fig 1:**
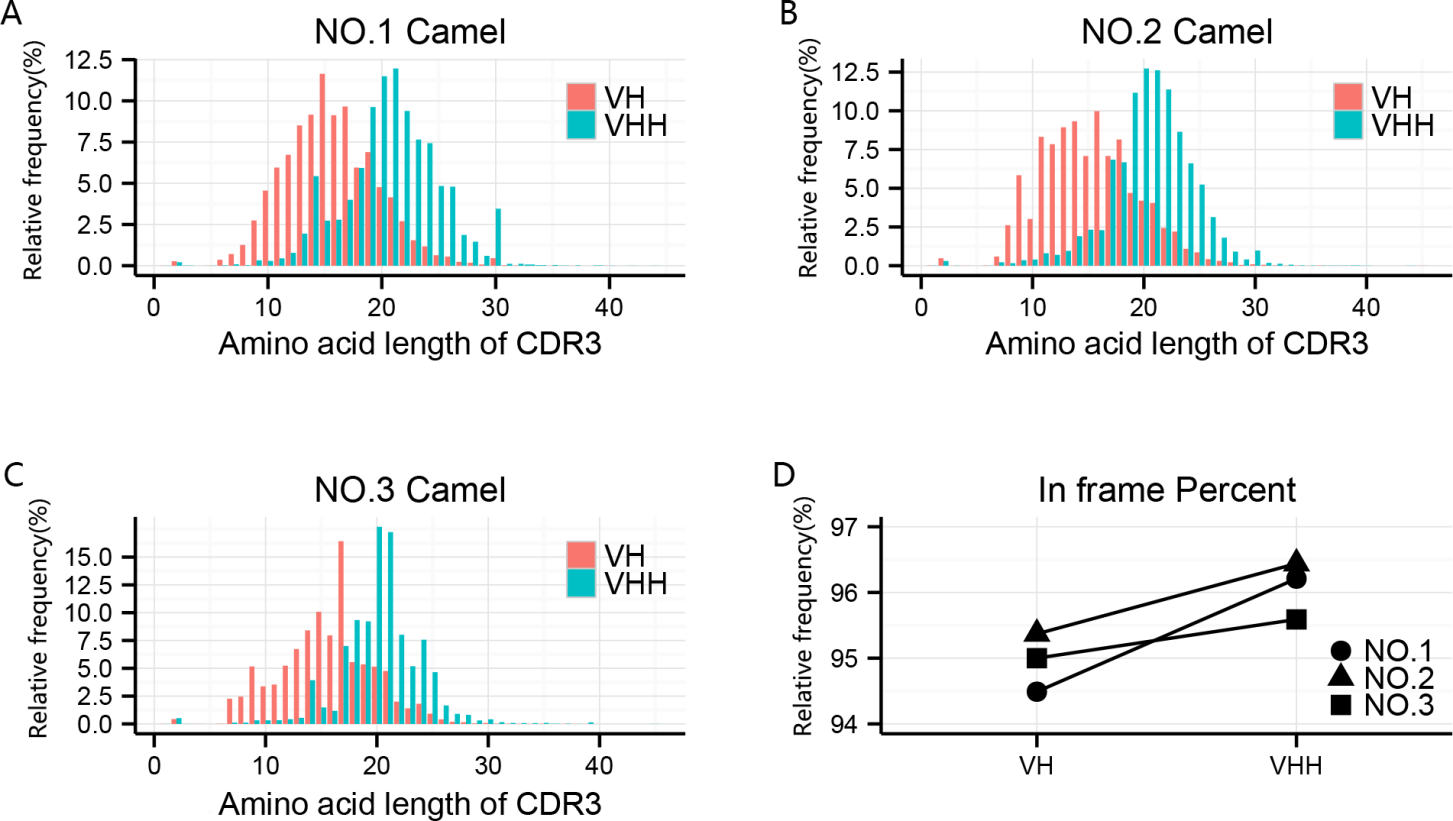
Schematic diagram of the CDR3 (AA)-relative analysis and in-frame percent (ORF) statistics. **a-c** The CDR3-related analysis of the NO.1, NO.2, and NO.3 camels including CDR3 length (AA) and proportion; **d** The comparison of in-frame percent of antibody variable region genes between VH and VHH clones of three camels.

The Mi-Seq sequencing runs yielded an average of 546,192 raw reads per sample. The rate of pair-end reads being merged into contigs was more than 91% (Table 1). In a previous study [27] the CDR3 length of a portion of the VHH region was reported to be longer than that of the heavy chain variable region genes of the classical antibody (VH region). In this study, we verified that the average length of the Nbs’ CDR3 is five amino acids (AAs) longer than that of conventional antibodies based on three different camels’ data (18 AAs versus 13AAs). In addition, the two antibody clones (VHs and VHHs) were compared across several different dimensions (Fig 1A–1C). Calculating the in-frame percentage, representing the proportion of antibody clones containing the correct open reading frame, demonstrated an increasing trend from VH to VHH clones, however the *P* value was not significant (*P* = 0.075, Fig 1D).

**Table 1:** Sequencing data overview of three camel samples.

Sample ID	Raw data	Clean Data (%)	Merged (%)	V-align (%)	J-align (%)	VJ-align (%)	Effective data	Effective data/raw data (%)
**1-VH**	585688	74.3	92.09	71.31	91.66	70.46	351209	59.97
**1-VHH**	610474	66.74	91.71	53.27	90.82	52.54	309782	50.74
**2-VH**	461284	73.69	92.47	70.18	91.72	69.29	272373	59.05
**2-VHH**	540738	64.22	91.71	53.67	91.21	52.82	266320	49.25
**3-VH**	544848	75.67	91.95	58.02	88.69	57.33	310279	56.95
**3-VHH**	573617	71.64	91.12	45.52	90.02	44.84	292751	51.04

Sample ID: “1-VH” and “1-VHH” represent the NO.1 camel`s conventional antibody and nanobody clones, respectively. The same nomenclature is used in the other samples. Raw data are the total number of reads; Clean data (%) is the rate of the filtered sequence reads, with the low-quality data removed; Merged (%) is the percent of sequences generated by merging the pair-end reads to one intact read; V-align (%), J-align (%) and VJ-align (%) are percentages of the sequencing data that were matched with the IMGT reference sequences; Effective data are the number of reads after filtering; Effective data/raw data (%) is the percent of raw data that remained after filtering.

### Conservation and diversity evaluation

**Fig 2:**
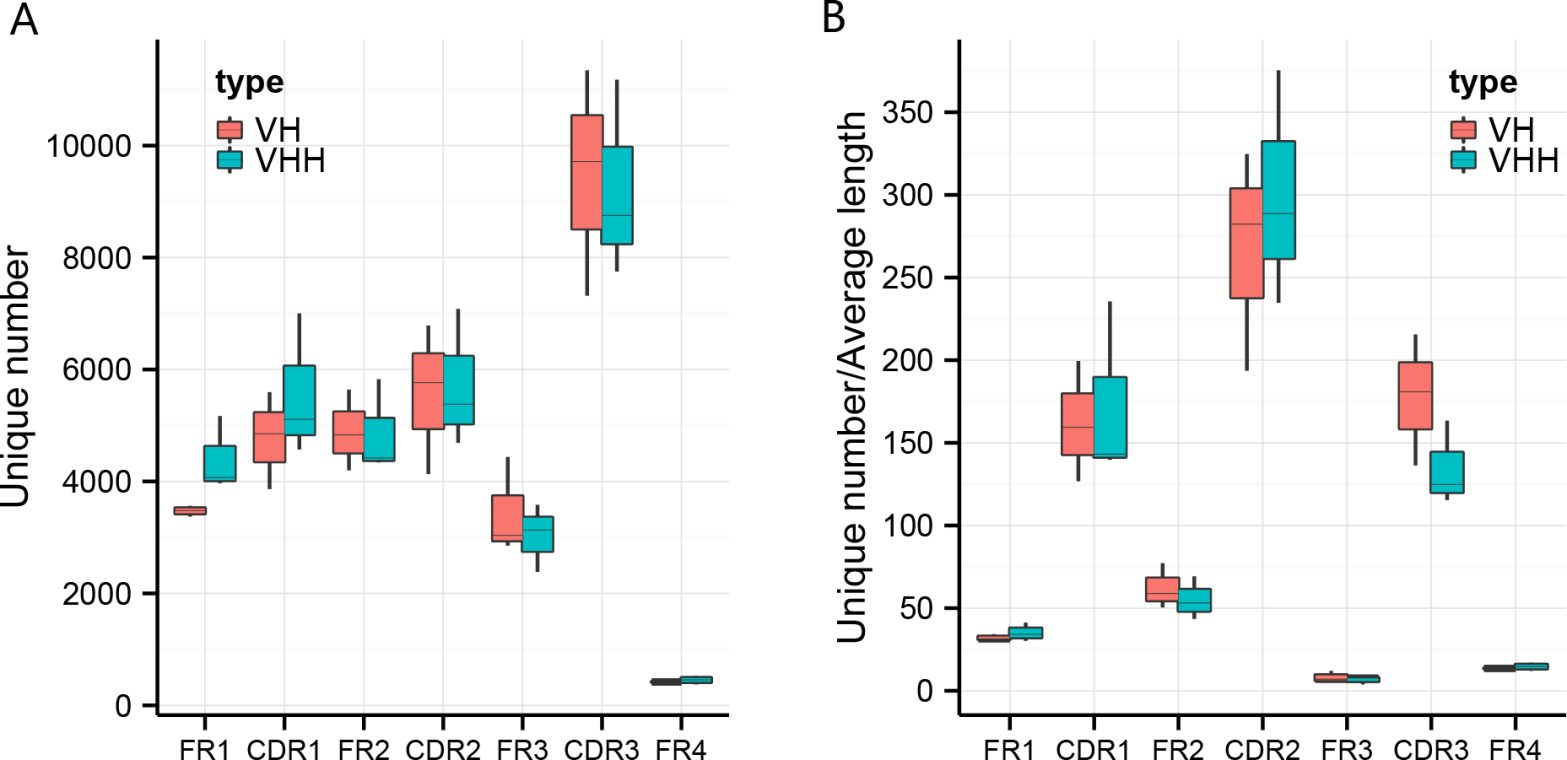
Box plots of the unique sequence numbers of each sub-region and diversity evaluation. Unique number represents the number of unique sequences within the reads. **a** The unique number of sub-regions including FR1, CDR1, FR2, CDR2, FR3, CDR3, and FR4 in three samples. **b** Diversity evaluation using the normalized unique number by length, which equals to unique number divided by average length of each sub-region. Box plot explanation: upper horizontal line of box, 75th percentile; lower horizontal line of box, 25th percentile; horizontal bar within box, the median of the three samples’ data; upper end of the whisker, maximum of the three samples’ data; lower end of the whisker, minimum of the three samples’ data.

The numbers of unique variable regions of each antibody, represented by the number of unique sequence reads, are shown in Fig 2A. The average number of unique CDR3s from each sample was 9459 and 9226 for VH clones and VHH clones, respectively. In order to evaluate the diversity of VH and VHH clones among sub-regions, irrespective of the effect of region length, we normalized the unique clone number by the average length of each sub-region (Fig 2B). Examination of this analysis suggested the following: (1) it was consistent with the fact that the framework regions (FRs) are more conserved than the CDRs, (2) the FR2 sub-region is less conserved than other FR sub-regions, (3) the diversity of CDR2 region is higher than all other sub-regions.

### Mutation rate analysis

**Fig 3:**
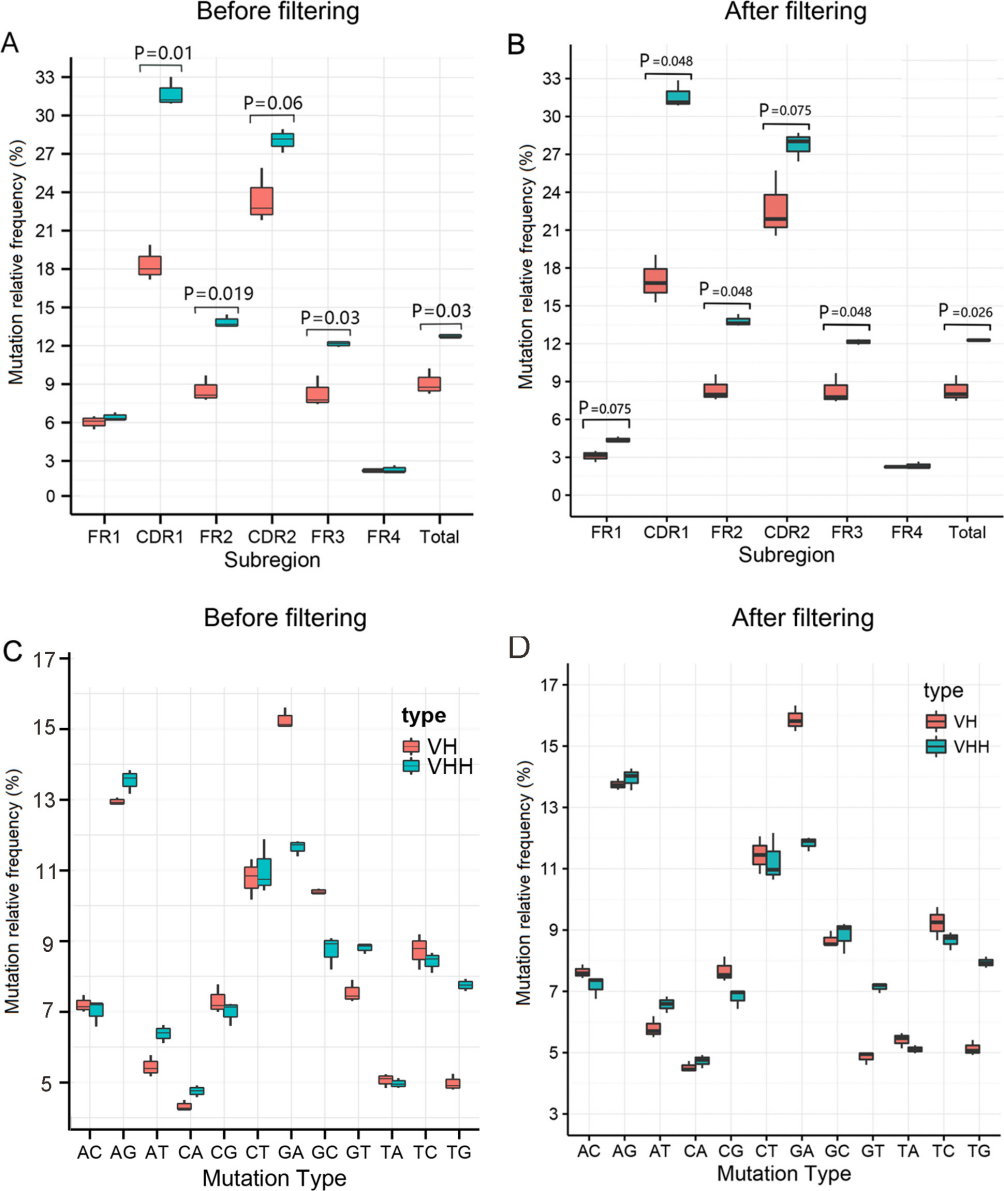
Box plots of the mutation rate analysis. **a** The mutation rates of each sub-region including FR1, CDR1, FR2, CDR2, FR3, and FR4 in three samples before filtering. Mutations are defined as mismatches with the IMGT references on each sample’s sequences. **b** The mutation rates of each sub-region including FR1, CDR1, FR2, CDR2, FR3, and FR4 in three samples after filtering. **c** Statistics of specific nucleic acid mutations of the VH and VHH clones before filtering. **d** Statistics of specific nucleic acid mutations of the VH and VHH clones after filtering. Box plot explanation: upper horizontal line of box, 75th percentile; lower horizontal line of box, 25th percentile; horizontal bar within box, the median of the three samples’ data; upper end of the whisker, maximum of the three samples’ data; lower end of the whisker, minimum of the three samples’ data.

Alignments of the variable (V) and joining (J) segments as well as mutation rate analysis were performed as previously described [24] with the Arabian camel V and llama J segment references from IMGT. It is important to note that the incomplete references in the IMGT could result in a partial “false” high mutation. In order to correct for this, we filtered some sequences which align to an improper V germline reference based upon criteria determined in S1 and S2 Figs. The S1 and S2 Tables show the detailed aligning and filtering information of the S2 Fig. As shown in Fig 3A and 3B, we respectively show the mutation rate analysis results before and after filtering. However, the decrease of the mutation rate is limited. It suggests that the IR of the camel’s BCR probably has a natural high mutation rate for both VHs and VHHs. After filtering, the VHH clones had a higher mutation rate (12.280%) than the VH clones (8.325%, *P* < 0.05, Fig 3B). The most common mutations were G to A in VH and A to G in VHH (Fig 3C and 3D), which has no change before and after filtering.

### Confirmation of the amino acid characteristics

**Fig 4:**
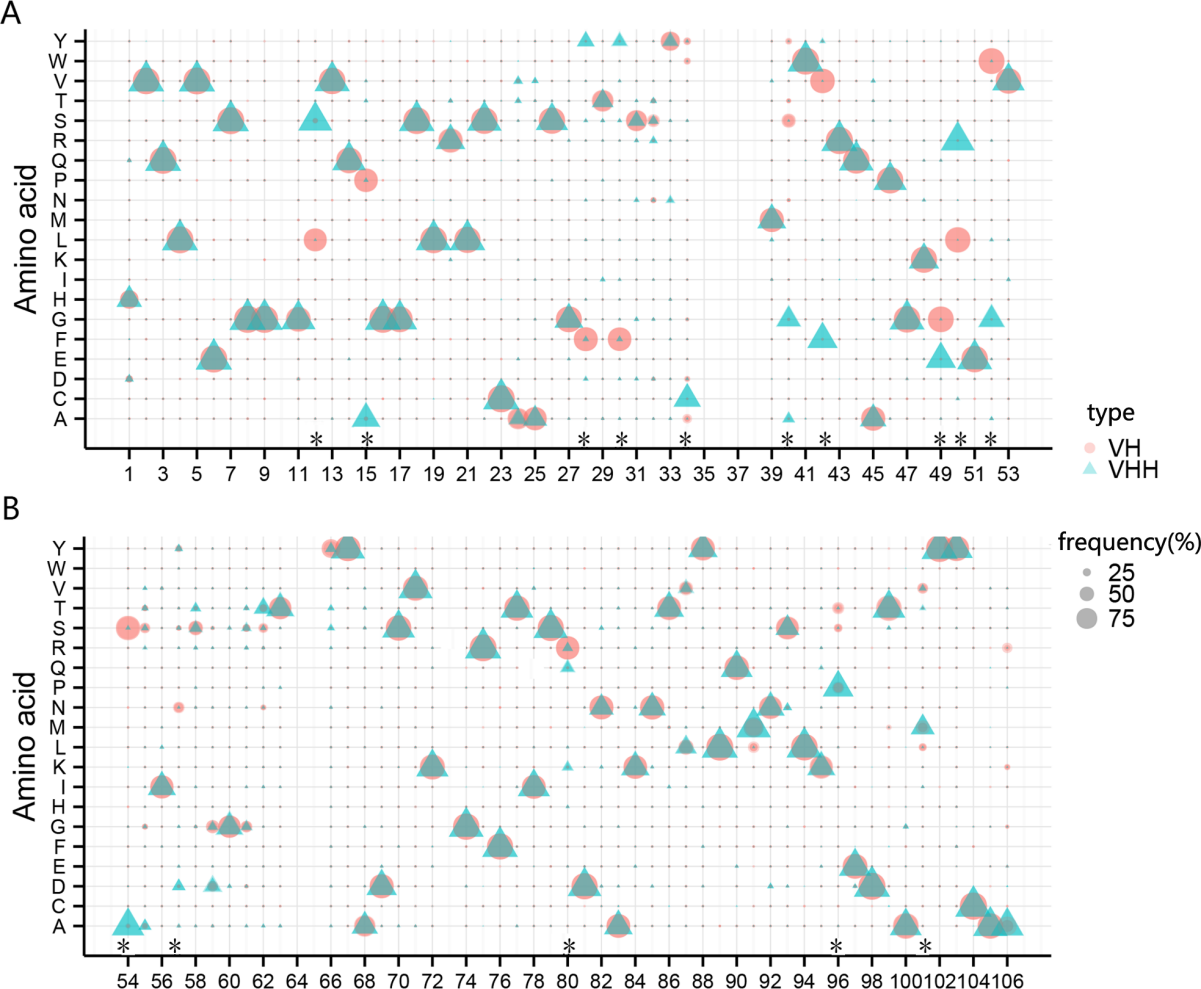
Amino acids frequency distribution of each position for VH and VHH clones. The horizontal axis lists the actual positions of the amino acids in IMGT references of the Arabian camel. The vertical axis lists abbreviations of twenty different amino acids. The circles denote VH clones and the triangles, VHHs. The size represents the proportion of clone number. The asterisk-labeled positions represent the amino acids which have little consistency between VH and VHH clones.

The occurrence and distribution of special AAs within the FR and CDR regions were also analyzed because of their close associations with the protein structure, solubility, and heavy-light chain interaction. We focused our analysis on specific AAs such as NO.12 in FR1, NO.28 in CDR1, NO.42/49/50/52 in FR2, and NO.80 in FR3 (Table 2 and Fig 4). In these positions, high AA concordance was observed among the same type of antibody clones, whereas there was little concordance between VH and VHH clones; as has been reported by others [27–30]. These characteristic AA substitutions between the VHs and the VHHs are probably an adaptation to increase the solubility and stability; a necessity as a result of the loss of the light chain and CH1 region. For instance, substitutions at residue positions NO.49/50/52, previously described by Davies and Riechmann [21] to result in an increase in solubility of the Nbs, were also seen in our data.

**Table 2:** Specific amino acids differences between VH and VHH clones of three camels.

Positions	12	15	28	30	34	40	42	49	50	52	54	57	80	96	101
**VH**	L	P	F	F	Y	S	V	G	L	W	S	N	R	P	V
					A	Y						Y		T	M
					W	T						S		S	L
					D	N						D			
**VHH**	S	A	Y	Y	C	G	F	E	C	F	A	D	Q	P	M
			F	F		A	Y	Q	R	G		Y	R		V
			D	S						L		S	K		T
			S							W					

Positions refer to the absolute position of the amino acids in IMGT references of the Arabian camel. VH and VHH denote the clones of the variable regions of conventional antibodies and HCAbs, respectively. Single letters in the table are abbreviations for amino acids. Multiple AAs are included at some positions to add up to a combined appearance frequency of >50%. The AA appearance frequency (shown in S1 File.) at a position is defined as: (total specific AA) / (total AAs at that position).

We also observed other substitutions that could lead to additional structural alterations. From our data one of the alterations is observed at position NO.54, where a serine (Ser, hydrophilic) in VH is changed to an aspartic acid (Asp, acidic) in VHH. Similar phenomenon could also be found at position NO.40. These two substitutions may contribute to small structural alterations in the heavy-light chain interaction or result in a folding over the inner VHH domain (Table 2 and Fig 4). Furthermore, at the NO.57 position, the AA with the highest frequency of usage is the Asn residue in VHs, however it is the Asp residue in VHHs and the Pro96 residue frequency was increased by about 50% in VHHs, compared to that of VHs (S1 File). Another interesting substitution is at position NO.101, the hydrophilic Thr residue is utilized in VHHs as opposed to the hydrophobic leu residue that is 3^rd^ most common at this position in VHs (S1 File). However, the mechanisms and significance of this limited set of reported substitutions as well as their contributions to the structure and functional behaviors of VHH remain to be studied. In addition, the percentage of the twenty AAs at all positions can be found in S1 File.

### The distribution of cysteine (Cys) codons

**Fig 5:**
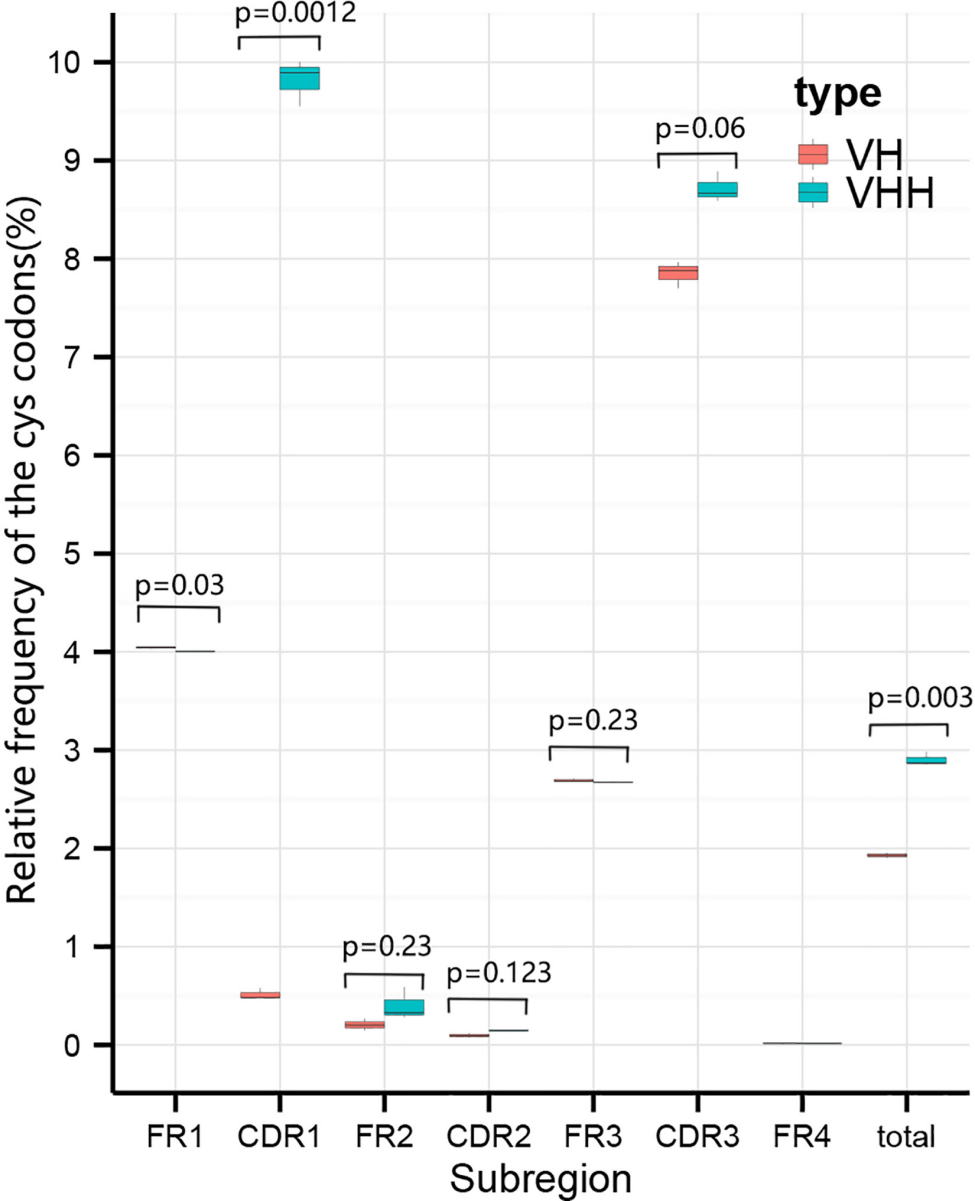
Box plots of the distribution and proportion of Cys codons in VH and VHH clones. The distribution and proportion of Cys codons (TGC and TGT) in each sub-region including FR1, CDR1, FR2, CDR2, FR3, CDR3, and FR4 in three samples was plotted. The percent equals to the total number of Cys codons divided by the total number of codons in each sub-region. Box plot explanation: upper horizontal line of box, 75th percentile; lower horizontal line of box, 25th percentile; horizontal bar within box, the median of the three samples’ data; upper end of the whisker, maximum of the three samples’ data; lower end of the whisker, minimum of the three samples’ data.

An excess of Cys encoding codons “TGT” and “TGC” were found in VHHs clones when compared against VHs clones as expected based on the fact that more disulfide bonds are needed for Nbs to maintain a stable structure [27]. In most sub-regions of the VH and VHH clones the presence of Cys codons was consistent with these codons being found in FR1, FR3 and CDR3 sub-regions more often than any other sub-region. However, the Cys codon of the CDR1 sub-region of the VHH clones was above 20 times more frequent than that of the VH clones (9.816% versus 0.514%, *P* < 0.005, Fig 5). This suggests that the Nbs may have altered the protein folding (possibly by forming a second internal disulfide bond [27]) to maintain a more stable structure compared to conventional antibodies.

### Non-classical VHH clones analysis

Previous studies [19, 31] have reported the existence of special VH(3) (clan III) family clones of VHH, which lack VHH hallmark AAs in the FR2 region and it is known that VH germline genes are promiscuous and can be used to generate VH-derived VHH clones. In this study we refer to these as non-classical VHH clones. We found non-classical VHH clones, formed by VH (3)-D-J gene rearrangement, accounted for about 8% of all clones.

**Fig 6:**
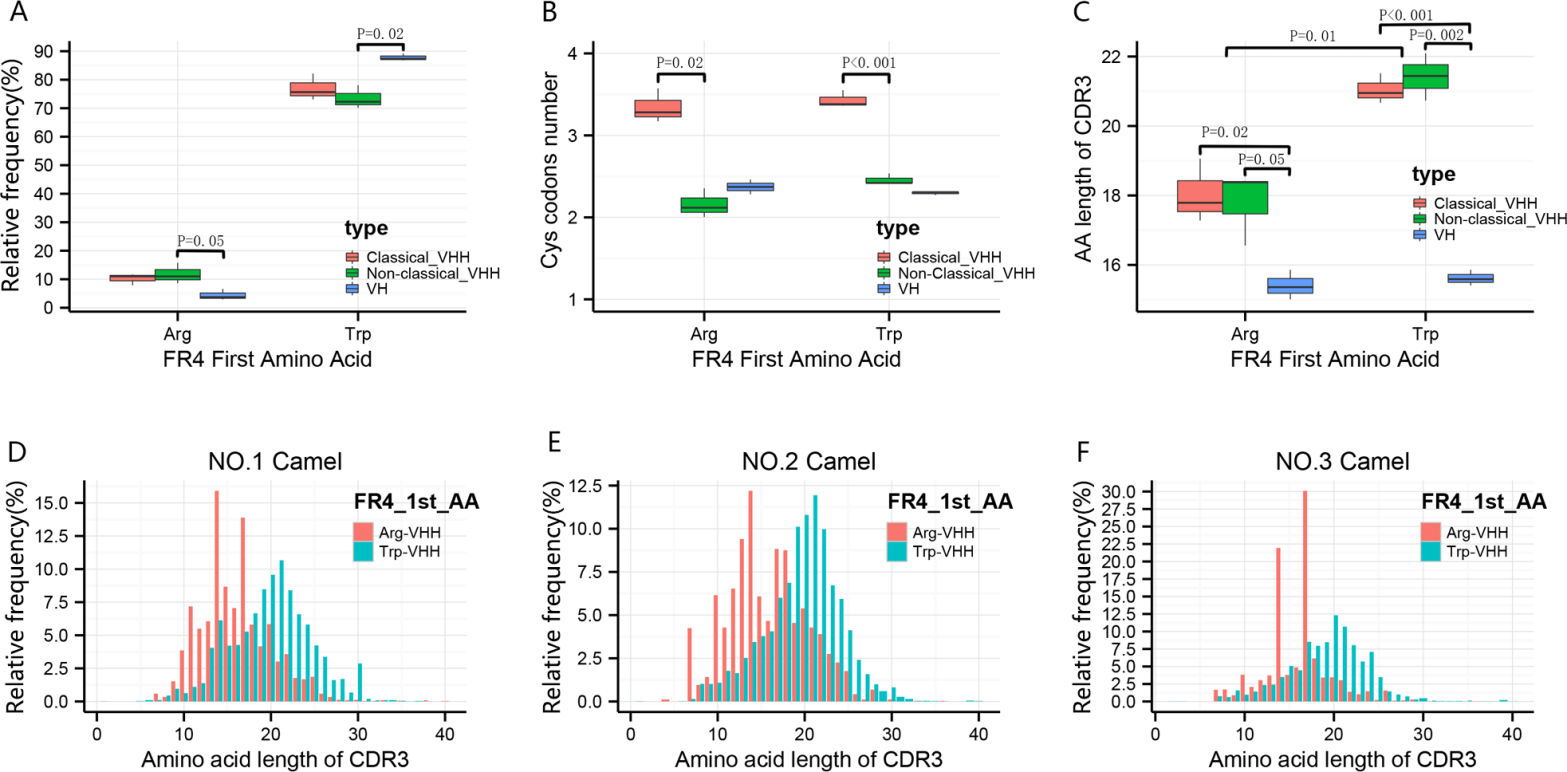
Box plots of the analysis of non-classical VHH clones. **a** The percentage of Trp-clones and Arg-clones (the clones whose first AA of FR4 are Trp and Arg) for classical VHH, non-classical VHH and VH(3) family clones. **b** Statistics of Cys codons within the Trp-clones and Arg-clones for classical VHH, non-classical VHH, and VH(3) family clones. **c** The average CDR3 length statistics of Trp-clones and Arg-clones for classical VHH, non-classical VHH and VH(3) family clones. **d-f** The proportion distribution of CDR3 average length of Trp-clones and Arg-clones. FR4_1st_AA represents the first AA of FR4 sub-region. Classical VHH represents the VHH having four FR2 hallmark amino acids. Non-classical VHH represents the VHH lacking four FR2 hallmark amino acids. VH represents the VH(3) (clan III) family clones. Box plot explanation: upper horizontal line of box, 75th percentile; lower horizontal line of box, 25th percentile; horizontal bar within box, the median of the three samples’ data; upper end of the whisker, maximum of the three samples’ data; lower end of the whisker, minimum of the three samples’ data.

According to previous reports [27, 31], the first AA of FR4 sub-region is a major component of the light chain contacting side of the heavy chain domain. The tryptophan (Trp) > arginine (Arg) substitution is common in non-classical VHH clones and these clones generally have a shorter CDR3 than clones lacking the Trp > Arg substitution. We performed comprehensive analyses associated with this substitution. From Fig 6A our data show that for both VH(3) family clones and VHHs (including classical and non-classical clones), the majority had no Trp > Arg substitution at the NO.1 position of FR4 and only a few clones had this Trp > Arg substitution. Compared with VH(3) clones, the Trp-clones (the clones whose first AA of FR4 is Trp) percentage of non-classical VHHs decreases significantly (*P* = 0.02) and the Arg-clones (the clones whose first AA of FR4 is Arg) percentage of non-classical VHHs increase (*P* = 0.05). As to the evidence of disulfide bonds (Fig 6B), we found that there is no difference in the number of Cys codons between VH(3) family clones and classical VHHs for either Trp-clones or Arg-clones and the classical VHHs have more Cys codons than VH(3) family clones and non-classical VHHs(Arg-clones, *P* = 0.02; Trp-clones, *P* < 0.001).

Lastly, we examined the CDR3 average length and its distribution. It was noticed that the CDR3 average length of Trp-clones is significantly longer (4AA) than that of Arg-clones (*P* = 0.01) in agreement with a previous study [31] and both classical VHHs and non-classical VHHs have a CDR3 average length that is longer than VH(3) family clones (Shown in Fig 6C). The statistics of the CDR3 length distribution of the Trp-clones and Arg-clones of the VHHs of three camels was examined and illustrated in Fig 6D–6F.

## Discussion

In recent years, there has been an increasing interest in camelid antibodies, particularly Nbs that are single-domain antibodies derived from the variable regions of camelid HCAbs. Nbs demonstrate great potential as high-affinity reagents for research, diagnostics, and therapeutics due in large part to their high specificity, small size (approximately 15 kDa), and straightforward bacterial expression [2]. However, little is known about the repertoires of the Bactrian camel antibodies. In this study, we used the massively parallel sequencing to describe, for the first time, the relatively complete VH and VHH repertoires of the Bactrian camel.

Repertoire diversity is a fundamental determinant of the competence of the immune system. Previous studies [19, 20] extrapolated the diversity of the camel immune repertoire based on a limited fraction of VJ combinations, which were chosen at random, thus making it difficult to accurately determine the actual diversity and the extent of clonal amplification within the repertoire. It is anticipated that the VHH clones would have a higher diversity than the VHs because of the VHHs accounting for 75% of the total number of antibodies. However, we found the diversities of VHs and VHHs are similar (Fig 2).

In addition, we have compared some characteristic amino acids, which may greatly impact the antibody structures and their properties. We validated, with tens of thousands of reads, the previous findings [27–30] based on low throughput sequences, that there are distinct AA differences between VH and VHH clones (Table 2 & Fig 4). For example, the conserved Leu (leucine) in VHs is replaced by Ser in VHHs at the position NO.12. In conventional Abs the Leu residue makes contact with the hydrophobic residues Phel49 and Pro150 of the CH1 domain. In view of the absence of the CH1 domain in the HCAbs the conservation of Leu is apparently no longer required [32]. In addition, we observed the substitution of a Tyr at positions 28 and 30 of VHH instead of a Phe as found in VH. These substitutions were originally described by Nguyen *et al* [30] and speculated to increase the potential repertoire. In addition, some limited reported substitutions, such as ones at NO. 57/96/101, were observed in our data.

Finally, we realized that the primer (CALL001) used to amplify the variable region genes in this study may exclude some genes originating from the VH(4) (Clan II) family; a previously described [19, 31] variable region gene family. In addition, the reverse primer (VHH-reverse), which anneals in the FR4, also completely matched the llama IgHJ4 germline gene and it has two or three mismatches annealing to the IgHJ2/3/5/6 germline genes. As such, the primer may preferentially amplify the V-D-J rearrangements that were generated from IgHJ4 gene to some degree. This bias may result in incomprehensive speculation about the V-J usage in Bactrian camels. Therefore, analyses such as V/J gene usage and the V-J pairing coverage rate were not conducted in this study. Such analyses remain to be explored using better-designed primers that would comprehensively amplify all variable region genes without bias.

In this study, through deep sequencing, we analyzed the characteristics of the VH and VHH immunoglobulin repertoire comparatively and verified some of the characteristics of previously reported amino acid composition. Our analysis of the Bactrian camel’s antibody repertoire will lay a solid foundation for multiple areas of biomedical research such as monoclonal antibody preparation and vaccine evaluation.

## Supporting Information

S1 Fig The aligning information of the third camel’s sequencing data on the mutation rate and the variation relative frequency. **a** The frequency distribution of mutation rates across each V germline gene. Mutation rates were devided into three ranges: <5%, 5%-10%, and >10%. Six top used V genes (S20, S30, S45, S50, S52, S72) have highest proportion of reads with mutation rate>10%, so they contribute largely to the exceptionally high mutation rate. **b** Relative frequencies of variation in each nucleotide of the six top used V germline genes. Mutation_rate = nucleotide variations number / sequence length. Nucleotide variation relative frequency = nucleotide variations number / total nucleotide number. It is a concept for a specific nucleotide site for all aligned reads. The cutoff line of 0.70 differentiates “hotspot” sites with high variation frequencies.
(TIF)

S2 Fig Schematic diagram of data filtering. **The 3-VH sample data were used to describe this filtering process.** 1. All the reads were aligned to the IGHV1S20 germline and we acquired about 15577 aligned reads. After analyses, the IGHV1S20 has three hotspots (NO. 163, 259, and 301) meeting the following filtering criterion: their variation relative frequencies are greater than 70%; the hotspots appear in the FR region, show consistency in three camels’ sequencing data, and have primary variation directions (such as, NO.163: G>A, 76.6%; NO. 259 C>G, 62.9% and NO. 301 A>C, 50.9%; the rates are all greater than 50%). 2. We speculate there exsits a potential more appropriate germline gene. Its nucleotides should probably be 163A, 259G, and 301C in these positions. 3. We filter out the aligned reads which have all above hotspots. For the aligned reads of IGHV1S20, there exist 5003 reads which have all the hotspots (NO. 163, 259, and 301, S1 Table). 4–6. The similar filtering is performed in the reads of the IGHV1S30. 7. So we filter out 10722 (5003 + 5719) reads from the 3-VH sample data (S2 Table).
(TIF)

S1 File The statistics of the appearance frequency of every type of amino acid at all 106 positions of three camels. Positions refer to the absolute position of the amino acids in IMGT references of the Arabian camel. “1-VH” and “1-VHH” represented the NO.1 camel`s conventional antibody and nanobody clones, respectively. The same nomenclature is used in the other samples.
(PDF)

S1 Table The relative data information of the aligned reads to the germline genes. Germline refers to the IMGT reference genes of the Arabian camel. Variation relative frequency = nucleotide variations number / total nucleotide number. It is a concept for a specific nucleotide site for all aligned reads. Hotspot refers to the nucleotide site which has high variation relative frequency, equal or greater than 70% (≥70%). The aligned reads numbers classified by the nucleotides in the hotspots were showed in the third colgroup. The common reads number refers to the number of the reads which have all above hotspots.
(XLSX)

S2 Table The number and percentage of the filtering reads. Sample ID refers to the names of the sequencing reads of all the three camels’ samples. Filtering reads number represents the number of the filtered out reads based on the designed criteria (S1 and S2 Figs). Filtering percentage refers to the proportion of the reads which are filtered out.
(XLSX)
