# Supplementary figures and images for: Comparative Analysis of Immune Repertoires between Bactrian Camel's Conventional and Heavy-Chain Antibodies
Source: PLoS One. 2016 Sep 2;11(9):e0161801. doi: 10.1371/journal.pone.0161801 (PMC5010241; doi:10.1371/journal.pone.0161801)

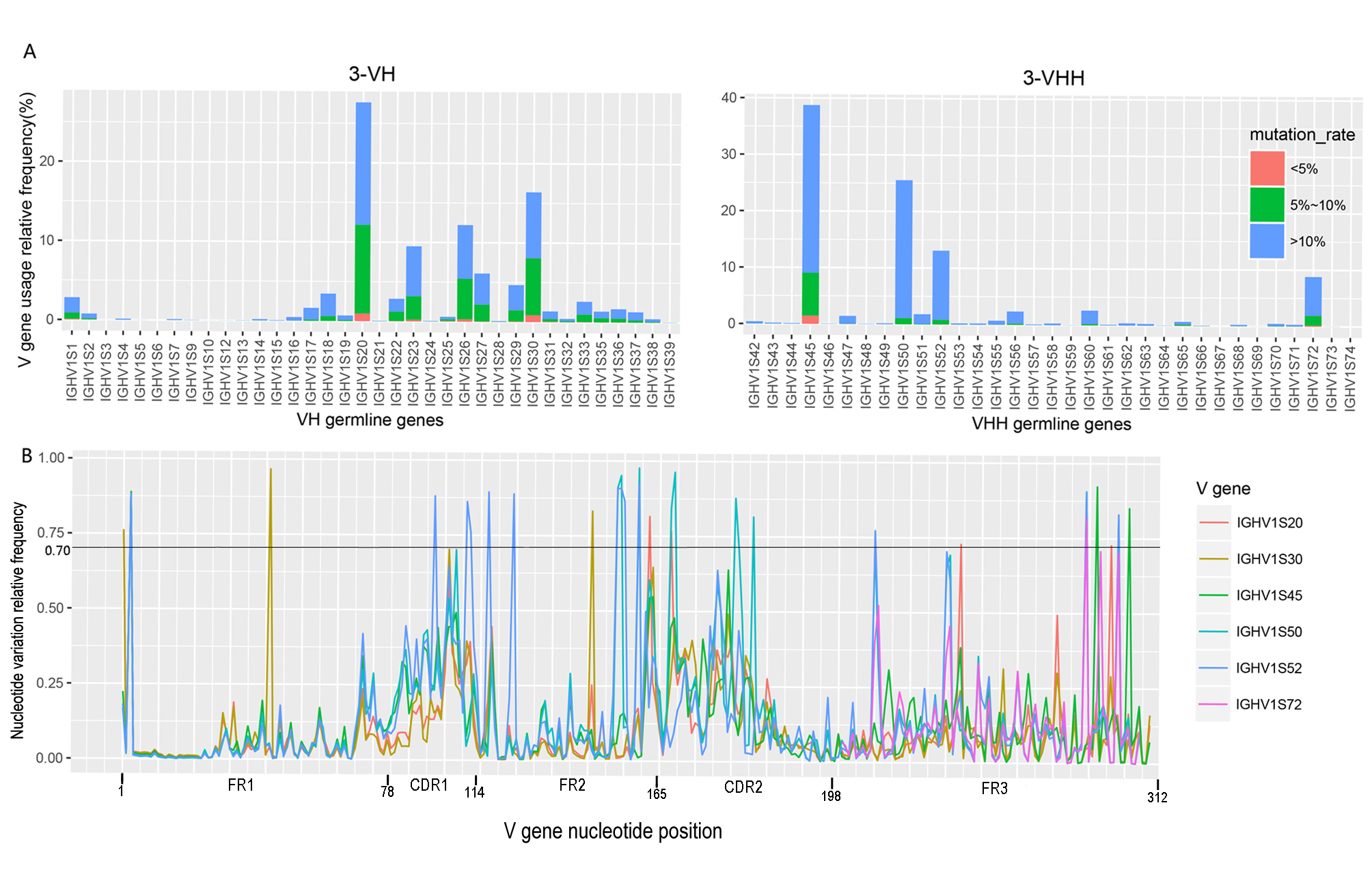

Supplement: S1 Fig — a The frequency distribution of mutation rates across each V germline gene. Mutation rates were devided into three ranges: <5%, 5%-10%, and >10%. Six top used V genes (S20, S30, S45, S50, S52, S72) have highest proportion of reads with mutation rate>10%, so they contribute largely to the exceptionally high mutation rate. b Relative frequencies of variation in each nucleotide of the six top used V germline genes. Mutation_rate = nucleotide variations number / sequence length. Nucleotide variation relative frequency = nucleotide variations number / total nucleotide number. It is a concept for a specific nucleotide site for all aligned reads. The cutoff line of 0.70 differentiates “hotspot” sites with high variation frequencies. (TIF) [file pone.0161801.s001.tif]

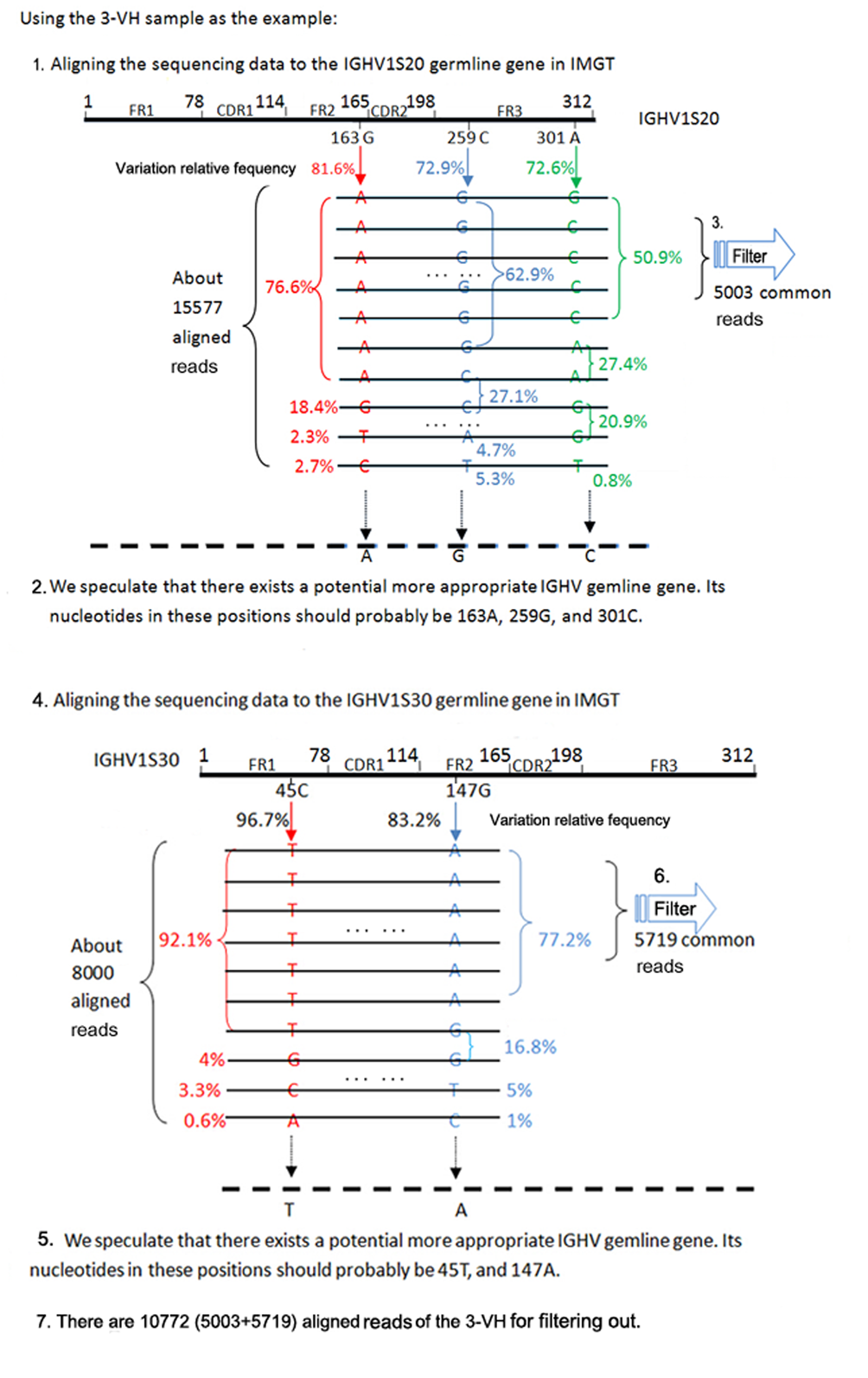

Supplement: S2 Fig — The 3-VH sample data were used to describe this filtering process. 1. All the reads were aligned to the IGHV1S20 germline and we acquired about 15577 aligned reads. After analyses, the IGHV1S20 has three hotspots (NO. 163, 259, and 301) meeting the following filtering criterion: their variation relative frequencies are greater than 70%; the hotspots appear in the FR region, show consistency in three camels’ sequencing data, and have primary variation directions (such as, NO.163: G>A, 76.6%; NO. 259 C>G, 62.9% and NO. 301 A>C, 50.9%; the rates are all greater than 50%). 2. We speculate there exsits a potential more appropriate germline gene. Its nucleotides should probably be 163A, 259G, and 301C in these positions. 3. We filter out the aligned reads which have all above hotspots. For the aligned reads of IGHV1S20, there exist 5003 reads which have all the hotspots (NO. 163, 259, and 301, S1 Table). 4–6. The similar filtering is performed in the reads of the IGHV1S30. 7. So we filter out 10722 (5003 + 5719) reads from the 3-VH sample data (S2 Table). (TIF) [file pone.0161801.s002.tif]
