# Supplementary material for: Comparative Analysis of Immune Repertoires between Bactrian Camel's Conventional and Heavy-Chain Antibodies
Source: PLoS One. 2016 Sep 2;11(9):e0161801. doi: 10.1371/journal.pone.0161801 (PMC5010241; doi:10.1371/journal.pone.0161801)
Supplement: S1 File — Positions refer to the absolute position of the amino acids in IMGT references of the Arabian camel. “1-VH” and “1-VHH” represented the NO.1 camel`s conventional antibody and nanobody clones, respectively. The same nomenclature is used in the other samples. (PDF) [file pone.0161801.s003.pdf]

Table S3: 1-VH

The positions of amino acids	Amino acids	Counts	Percentage(%)
1	H	34782	66.05139
1	D	11092	21.06383
1	Q	4289	8.14486
1	S	882	1.67493
1	E	784	1.48882
1	M	269	0.51083
1	L	162	0.30764
1	P	95	0.18041
1	A	80	0.15192
1	Y	76	0.14432
1	R	41	0.07786
1	N	32	0.06077
1	G	24	0.04558
1	W	18	0.03418
1	C	12	0.02279
1	V	10	0.01899
1	I	5	0.0095
1	K	4	0.0076
1	F	1	0.0019
1	T	1	0.0019
2	V	51345	97.20198
2	C	837	1.58454
2	M	168	0.31804
2	G	124	0.23475
2	L	100	0.18931
2	W	89	0.16849
2	A	65	0.12305
2	H	25	0.04733
2	E	19	0.03597
2	R	15	0.0284
2	S	15	0.0284
2	D	9	0.01704
2	Q	6	0.01136
2	F	4	0.00757
2	I	1	0.00189
2	P	1	0.00189
3	Q	56664	97.66287
3	A	780	1.34436
3	S	167	0.28783
3	C	103	0.17752
3	P	100	0.17235
3	V	52	0.08962
3	R	33	0.05688

3	K	29	0.04998
3	E	28	0.04826
3	L	27	0.04654
3	H	18	0.03102
3	W	9	0.01551
3	G	5	0.00862
3	M	5	0.00862
4	L	57368	97.93438
4	A	692	1.18133
4	W	157	0.26802
4	S	88	0.15023
4	P	73	0.12462
4	Q	66	0.11267
4	V	55	0.09389
4	M	52	0.08877
4	R	11	0.01878
4	G	5	0.00854
4	C	3	0.00512
4	H	3	0.00512
4	E	2	0.00341
4	D	1	0.00171
4	F	1	0.00171
4	T	1	0.00171
5	V	58441	98.33586
5	G	631	1.06175
5	W	207	0.34831
5	L	50	0.08413
5	A	32	0.05384
5	E	26	0.04375
5	M	21	0.03534
5	F	7	0.01178
5	C	5	0.00841
5	R	4	0.00673
5	S	4	0.00673
5	P	1	0.00168
5	T	1	0.00168
6	E	58681	98.42668
6	G	578	0.96949
6	S	127	0.21302
6	W	56	0.09393
6	A	49	0.08219
6	V	44	0.0738
6	M	39	0.06542
6	K	16	0.02684
6	Q	11	0.01845
6	D	6	0.01006

6	R	4	0.00671
6	Y	4	0.00671
6	C	2	0.00335
6	L	2	0.00335
7	S	58862	98.55504
7	V	511	0.85559
7	L	122	0.20427
7	F	67	0.11218
7	A	57	0.09544
7	Y	38	0.06362
7	P	27	0.04521
7	T	14	0.02344
7	E	12	0.02009
7	G	6	0.01005
7	W	4	0.0067
7	C	3	0.00502
7	D	1	0.00167
7	R	1	0.00167
8	G	59100	98.82778
8	W	518	0.86621
8	L	52	0.08696
8	E	30	0.05017
8	H	29	0.04849
8	R	25	0.04181
8	V	13	0.02174
8	D	11	0.01839
8	S	10	0.01672
8	A	4	0.00669
8	Q	4	0.00669
8	P	3	0.00502
8	F	1	0.00167
8	M	1	0.00167
9	G	59516	99.34069
9	E	131	0.21866
9	R	126	0.21031
9	V	86	0.14355
9	A	21	0.03505
9	M	10	0.01669
9	D	5	0.00835
9	K	5	0.00835
9	P	3	0.00501
9	S	3	0.00501
9	F	2	0.00334
9	C	1	0.00167
9	W	1	0.00167
9	Y	1	0.00167

10	NA	0	0
11	G	54591	90.98955
11	D	2707	4.51189
11	A	1031	1.71842
11	E	353	0.58836
11	R	346	0.5767
11	N	314	0.52336
11	S	239	0.39835
11	V	165	0.27501
11	T	78	0.13001
11	Q	62	0.10334
11	C	29	0.04834
11	H	21	0.035
11	K	21	0.035
11	F	15	0.025
11	Y	8	0.01333
11	L	6	0.01
11	W	5	0.00833
11	I	4	0.00667
11	M	2	0.00333
12	L	51095	85.11152
12	S	7230	12.04338
12	V	393	0.65464
12	F	214	0.35647
12	A	209	0.34814
12	T	180	0.29984
12	M	169	0.28151
12	W	156	0.25986
12	P	108	0.1799
12	E	83	0.13826
12	R	55	0.09162
12	Y	53	0.08828
12	Q	24	0.03998
12	K	17	0.02832
12	H	15	0.02499
12	G	13	0.02165
12	I	9	0.01499
12	D	5	0.00833
12	N	4	0.00666
12	C	1	0.00167
13	V	58169	96.81763
13	A	996	1.65776
13	G	311	0.51763
13	M	169	0.28129
13	E	127	0.21138
13	I	113	0.18808

13	L	77	0.12816
13	C	45	0.0749
13	W	26	0.04327
13	T	12	0.01997
13	F	11	0.01831
13	R	9	0.01498
13	Q	5	0.00832
13	P	4	0.00666
13	S	4	0.00666
13	D	2	0.00333
13	K	1	0.00166
14	Q	57269	95.28159
14	R	876	1.45745
14	E	596	0.9916
14	H	321	0.53407
14	A	239	0.39764
14	L	234	0.38932
14	P	178	0.29615
14	K	165	0.27452
14	S	65	0.10814
14	T	54	0.08984
14	C	31	0.05158
14	N	19	0.03161
14	G	16	0.02662
14	D	15	0.02496
14	M	12	0.01997
14	V	9	0.01497
14	F	2	0.00333
14	W	2	0.00333
14	Y	2	0.00333
15	P	52835	87.88111
15	A	5040	8.38309
15	T	730	1.21422
15	S	651	1.08282
15	V	348	0.57883
15	L	192	0.31936
15	I	84	0.13972
15	F	49	0.0815
15	D	39	0.06487
15	H	35	0.05822
15	R	34	0.05655
15	N	30	0.0499
15	G	22	0.03659
15	E	14	0.02329
15	Y	10	0.01663
15	Q	7	0.01164

15	M	1	0.00166
16	G	59451	98.81983
16	W	232	0.38563
16	E	165	0.27426
16	R	149	0.24767
16	A	75	0.12467
16	V	36	0.05984
16	L	29	0.0482
16	S	7	0.01164
16	D	5	0.00831
16	T	5	0.00831
16	P	3	0.00499
16	C	1	0.00166
16	N	1	0.00166
16	Q	1	0.00166
16	Y	1	0.00166
17	G	57771	95.9986
17	E	1240	2.06052
17	R	480	0.79762
17	D	151	0.25092
17	V	108	0.17946
17	A	102	0.16949
17	K	73	0.1213
17	T	49	0.08142
17	W	47	0.0781
17	Q	44	0.07312
17	S	42	0.06979
17	N	34	0.0565
17	L	21	0.0349
17	M	6	0.00997
17	Y	6	0.00997
17	F	2	0.00332
17	P	2	0.00332
17	C	1	0.00166
18	S	59097	98.08142
18	T	393	0.65225
18	P	286	0.47467
18	F	153	0.25393
18	A	146	0.24231
18	G	36	0.05975
18	Y	29	0.04813
18	L	25	0.04149
18	V	24	0.03983
18	C	14	0.02324
18	I	13	0.02158
18	E	9	0.01494

18	R	8	0.01328
18	M	6	0.00996
18	N	6	0.00996
18	D	5	0.0083
18	H	1	0.00166
18	Q	1	0.00166
18	W	1	0.00166
19	L	59024	97.85957
19	V	331	0.54879
19	P	292	0.48413
19	R	212	0.35149
19	M	161	0.26693
19	Q	150	0.24869
19	A	49	0.08124
19	T	26	0.04311
19	I	20	0.03316
19	H	16	0.02653
19	S	15	0.02487
19	W	8	0.01326
19	G	4	0.00663
19	E	3	0.00497
19	F	2	0.00332
19	D	1	0.00166
19	Y	1	0.00166
20	R	54434	90.22559
20	T	2868	4.75378
20	K	1228	2.03544
20	S	738	1.22325
20	G	366	0.60665
20	N	169	0.28012
20	I	158	0.26189
20	M	123	0.20388
20	A	65	0.10774
20	L	40	0.0663
20	Q	39	0.06464
20	V	36	0.05967
20	E	29	0.04807
20	H	16	0.02652
20	D	8	0.01326
20	W	7	0.0116
20	C	3	0.00497
20	P	3	0.00497
20	F	1	0.00166
21	L	58428	96.72069
21	V	895	1.48157
21	I	543	0.89887

21	P	284	0.47013
21	F	101	0.16719
21	H	98	0.16223
21	R	26	0.04304
21	G	13	0.02152
21	S	7	0.01159
21	T	6	0.00993
21	A	3	0.00497
21	Q	2	0.00331
21	C	1	0.00166
21	E	1	0.00166
21	W	1	0.00166
22	S	58888	97.38866
22	A	541	0.8947
22	T	461	0.7624
22	P	265	0.43826
22	F	101	0.16703
22	V	44	0.07277
22	H	37	0.06119
22	D	25	0.04134
22	L	25	0.04134
22	Y	25	0.04134
22	I	16	0.02646
22	N	13	0.0215
22	G	8	0.01323
22	Q	8	0.01323
22	E	4	0.00662
22	C	3	0.00496
22	R	2	0.00331
22	M	1	0.00165
23	C	60012	99.11148
23	R	246	0.40628
23	Y	101	0.1668
23	S	97	0.1602
23	G	32	0.05285
23	W	30	0.04955
23	F	13	0.02147
23	L	9	0.01486
23	A	3	0.00495
23	P	3	0.00495
23	V	2	0.0033
23	D	1	0.00165
23	T	1	0.00165
24	A	47240	77.99112
24	V	5681	9.37908
24	T	3152	5.20381

24	S	1619	2.6729
24	E	1102	1.81935
24	L	382	0.63066
24	G	373	0.61581
24	K	232	0.38302
24	Q	218	0.35991
24	I	217	0.35826
24	R	174	0.28727
24	D	95	0.15684
24	P	40	0.06604
24	N	19	0.03137
24	M	11	0.01816
24	F	8	0.01321
24	H	8	0.01321
25	A	52344	86.33207
25	T	2822	4.65438
25	V	2293	3.78189
25	G	1520	2.50697
25	S	446	0.7356
25	P	312	0.51459
25	D	185	0.30512
25	I	152	0.2507
25	F	105	0.17318
25	Y	96	0.15833
25	C	84	0.13854
25	H	64	0.10556
25	L	57	0.09401
25	R	48	0.07917
25	E	32	0.05278
25	M	31	0.05113
25	N	28	0.04618
25	K	5	0.00825
25	W	4	0.0066
25	Q	3	0.00495
26	S	58311	96.08958
26	G	578	0.95248
26	P	480	0.79098
26	T	391	0.64432
26	A	316	0.52073
26	N	174	0.28673
26	F	173	0.28508
26	D	68	0.11206
26	Y	68	0.11206
26	L	32	0.05273
26	R	28	0.04614
26	H	17	0.02801

26	I	15	0.02472
26	C	10	0.01648
26	E	7	0.01154
26	V	6	0.00989
26	Q	5	0.00824
26	W	3	0.00494
26	K	1	0.00165
26	M	1	0.00165
27	G	57771	95.16052
27	E	1052	1.73286
27	A	502	0.8269
27	R	491	0.80878
27	V	191	0.31462
27	D	153	0.25202
27	K	102	0.16801
27	S	83	0.13672
27	Q	80	0.13178
27	T	75	0.12354
27	L	55	0.0906
27	I	53	0.0873
27	N	43	0.07083
27	M	29	0.04777
27	P	15	0.02471
27	F	7	0.01153
27	W	3	0.00494
27	H	2	0.00329
27	C	1	0.00165
27	Y	1	0.00165
28	F	53780	88.53549
28	L	2031	3.34354
28	Y	1503	2.47432
28	S	1253	2.06276
28	I	597	0.98281
28	V	529	0.87087
28	D	232	0.38193
28	N	145	0.23871
28	A	121	0.1992
28	H	109	0.17944
28	W	103	0.16956
28	G	89	0.14652
28	T	57	0.09384
28	R	56	0.09219
28	P	43	0.07079
28	C	28	0.0461
28	M	28	0.0461
28	K	19	0.03128

28	E	17	0.02799
28	Q	4	0.00659
29	T	48518	79.83874
29	A	3035	4.99424
29	S	2406	3.95919
29	I	1831	3.013
29	P	1400	2.30377
29	D	671	1.10416
29	R	513	0.84417
29	N	368	0.60556
29	L	359	0.59075
29	V	345	0.56771
29	G	303	0.4986
29	F	207	0.34063
29	M	190	0.31265
29	K	178	0.29291
29	H	127	0.20898
29	E	119	0.19582
29	Q	115	0.18924
29	Y	70	0.11519
29	W	13	0.02139
29	C	2	0.00329
30	F	51997	85.52138
30	L	2384	3.92105
30	S	1664	2.73684
30	Y	1125	1.85033
30	V	1011	1.66283
30	I	889	1.46217
30	A	467	0.76809
30	D	232	0.38158
30	T	226	0.37171
30	G	203	0.33388
30	R	117	0.19243
30	N	100	0.16447
30	W	89	0.14638
30	H	72	0.11842
30	C	64	0.10526
30	P	56	0.09211
30	M	30	0.04934
30	Q	27	0.04441
30	E	24	0.03947
30	K	23	0.03783
31	S	47374	77.90239
31	G	2701	4.44156
31	R	2245	3.69171
31	T	2171	3.57002

31	N	2051	3.37269
31	D	1573	2.58666
31	M	609	1.00145
31	A	543	0.89292
31	K	399	0.65612
31	E	258	0.42426
31	I	234	0.38479
31	V	146	0.24008
31	H	115	0.18911
31	C	95	0.15622
31	P	87	0.14306
31	Y	63	0.1036
31	L	59	0.09702
31	Q	55	0.09044
31	F	18	0.0296
31	W	16	0.02631
32	S	26918	44.24392
32	N	9289	15.26792
32	T	7744	12.72847
32	D	3813	6.26726
32	R	3005	4.93918
32	G	2073	3.4073
32	A	1644	2.70217
32	I	1129	1.85569
32	Y	1059	1.74063
32	K	848	1.39382
32	V	636	1.04536
32	L	593	0.97469
32	E	441	0.72485
32	M	417	0.6854
32	P	398	0.65417
32	H	363	0.59665
32	F	209	0.34352
32	Q	162	0.26627
32	W	56	0.09204
32	C	43	0.07068
33	Y	42519	69.84411
33	S	2949	4.84419
33	N	2858	4.69471
33	H	2564	4.21177
33	F	1564	2.56911
33	A	1553	2.55105
33	T	1148	1.88577
33	W	1035	1.70015
33	V	936	1.53753
33	D	734	1.20571

33	R	673	1.10551
33	G	490	0.8049
33	C	453	0.74412
33	L	398	0.65378
33	K	256	0.42052
33	I	240	0.39424
33	E	190	0.3121
33	Q	151	0.24804
33	P	96	0.1577
33	M	70	0.11499
34	Y	14650	24.04714
34	A	11684	19.17862
34	W	10611	17.41735
34	D	7501	12.31247
34	G	3871	6.35403
34	S	2140	3.51269
34	C	1668	2.73793
34	H	1632	2.67884
34	R	1301	2.13552
34	V	1187	1.94839
34	T	1139	1.8696
34	F	882	1.44775
34	P	859	1.41
34	L	620	1.01769
34	E	344	0.56466
34	N	313	0.51377
34	I	266	0.43662
34	Q	113	0.18548
34	M	74	0.12147
34	K	67	0.10998
35	NA	0	0
36	NA	0	0
37	NA	0	0
38	NA	0	0
39	M	53967	88.47484
39	I	2597	4.25759
39	L	1375	2.25421
39	V	1159	1.90009
39	T	1022	1.67549
39	F	168	0.27542
39	A	113	0.18526
39	S	112	0.18362
39	W	105	0.17214
39	G	99	0.1623
39	K	91	0.14919
39	R	66	0.1082

39	P	62	0.10164
39	Q	22	0.03607
39	Y	14	0.02295
39	E	12	0.01967
39	N	9	0.01475
39	C	3	0.00492
39	H	1	0.00164
40	S	27031	44.21092
40	Y	8564	14.00697
40	T	6190	10.12414
40	N	5139	8.40516
40	G	4065	6.64857
40	H	3324	5.43661
40	A	2236	3.65712
40	I	1154	1.88744
40	F	944	1.54397
40	R	698	1.14162
40	M	469	0.76708
40	D	455	0.74418
40	C	287	0.46941
40	V	213	0.34838
40	K	141	0.23061
40	L	109	0.17828
40	W	56	0.09159
40	Q	28	0.0458
40	E	25	0.04089
40	P	13	0.02126
41	W	60994	99.30803
41	R	278	0.45263
41	C	52	0.08466
41	L	45	0.07327
41	Y	13	0.02117
41	S	12	0.01954
41	G	11	0.01791
41	F	7	0.0114
41	A	2	0.00326
41	H	2	0.00326
41	K	1	0.00163
41	P	1	0.00163
41	V	1	0.00163
42	V	55810	90.34838
42	I	2345	3.79622
42	F	1857	3.00622
42	A	587	0.95027
42	L	375	0.60707
42	G	349	0.56498

42	Y	231	0.37396
42	D	74	0.1198
42	S	55	0.08904
42	H	20	0.03238
42	N	13	0.02105
42	Q	12	0.01943
42	T	10	0.01619
42	P	9	0.01457
42	W	9	0.01457
42	K	7	0.01133
42	M	5	0.00809
42	C	2	0.00324
42	E	1	0.00162
42	R	1	0.00162
43	R	61587	99.27303
43	H	216	0.34817
43	C	133	0.21438
43	S	34	0.05481
43	L	27	0.04352
43	P	15	0.02418
43	G	12	0.01934
43	A	5	0.00806
43	Q	3	0.00484
43	F	2	0.00322
43	W	2	0.00322
43	T	1	0.00161
43	Y	1	0.00161
44	Q	60638	97.5609
44	R	577	0.92834
44	L	428	0.68861
44	H	234	0.37648
44	E	82	0.13193
44	K	49	0.07884
44	P	28	0.04505
44	S	28	0.04505
44	A	27	0.04344
44	M	27	0.04344
44	W	12	0.01931
44	Y	9	0.01448
44	V	7	0.01126
44	G	5	0.00804
44	D	2	0.00322
44	C	1	0.00161
45	A	54187	87.0418
45	T	2145	3.44556
45	G	1786	2.86889

45	V	1564	2.51229
45	P	746	1.19832
45	S	543	0.87223
45	I	394	0.63289
45	R	321	0.51563
45	L	254	0.40801
45	D	140	0.22489
45	N	52	0.08353
45	H	32	0.0514
45	F	29	0.04658
45	Y	24	0.03855
45	E	20	0.03213
45	C	8	0.01285
45	K	5	0.00803
45	M	3	0.00482
45	Q	1	0.00161
46	P	60750	97.46667
46	S	560	0.89846
46	A	310	0.49736
46	L	184	0.29521
46	Q	177	0.28398
46	T	113	0.1813
46	R	101	0.16204
46	H	49	0.07862
46	E	22	0.0353
46	V	14	0.02246
46	G	13	0.02086
46	I	13	0.02086
46	K	8	0.01284
46	W	7	0.01123
46	D	5	0.00802
46	C	2	0.00321
46	M	1	0.0016
47	G	61239	98.13156
47	E	562	0.90057
47	R	162	0.25959
47	A	117	0.18748
47	D	92	0.14742
47	W	75	0.12018
47	V	68	0.10897
47	K	26	0.04166
47	Q	23	0.03686
47	L	16	0.02564
47	S	12	0.01923
47	T	7	0.01122
47	H	3	0.00481

47	C	1	0.0016
47	M	1	0.0016
47	N	1	0.0016
48	K	59939	95.96228
48	R	632	1.01183
48	E	530	0.84853
48	Q	350	0.56035
48	T	214	0.34261
48	N	201	0.3218
48	M	186	0.29779
48	G	118	0.18892
48	A	84	0.13448
48	V	74	0.11847
48	S	60	0.09606
48	L	42	0.06724
48	I	14	0.02241
48	W	5	0.008
48	D	4	0.0064
48	P	4	0.0064
48	Y	2	0.0032
48	C	1	0.0016
48	F	1	0.0016
49	G	59973	95.88623
49	E	1258	2.01132
49	A	397	0.63473
49	R	203	0.32456
49	K	171	0.2734
49	T	136	0.21744
49	D	129	0.20625
49	V	78	0.12471
49	S	57	0.09113
49	Q	40	0.06395
49	W	33	0.05276
49	P	28	0.04477
49	N	15	0.02398
49	L	14	0.02238
49	C	4	0.0064
49	H	4	0.0064
49	F	3	0.0048
49	I	2	0.0032
49	Y	1	0.0016
50	L	59444	94.89631
50	P	879	1.40323
50	R	828	1.32182
50	F	803	1.28191
50	V	234	0.37356

50	I	145	0.23148
50	H	138	0.2203
50	Q	45	0.07184
50	M	42	0.06705
50	C	39	0.06226
50	S	11	0.01756
50	T	9	0.01437
50	A	6	0.00958
50	G	5	0.00798
50	Y	4	0.00639
50	N	3	0.00479
50	K	2	0.00319
50	W	2	0.00319
50	D	1	0.0016
50	E	1	0.0016
51	E	61193	97.60739
51	D	555	0.88527
51	Q	251	0.40036
51	G	221	0.35251
51	V	162	0.2584
51	K	136	0.21693
51	A	91	0.14515
51	N	48	0.07656
51	W	8	0.01276
51	R	7	0.01117
51	L	6	0.00957
51	M	4	0.00638
51	S	4	0.00638
51	F	3	0.00479
51	H	2	0.00319
51	C	1	0.0016
51	T	1	0.0016
52	W	60340	96.14404
52	G	752	1.19822
52	R	646	1.02932
52	C	293	0.46686
52	S	155	0.24697
52	Y	151	0.2406
52	L	137	0.21829
52	F	79	0.12588
52	A	62	0.09879
52	E	33	0.05258
52	Q	24	0.03824
52	P	19	0.03027
52	V	19	0.03027
52	M	16	0.02549

52	T	14	0.02231
52	H	6	0.00956
52	K	5	0.00797
52	D	4	0.00637
52	I	4	0.00637
52	N	1	0.00159
53	V	59279	94.36326
53	I	1553	2.47214
53	L	1218	1.93887
53	M	262	0.41706
53	A	258	0.4107
53	D	62	0.09869
53	Q	49	0.078
53	E	40	0.06367
53	G	33	0.05253
53	T	25	0.0398
53	F	22	0.03502
53	S	6	0.00955
53	C	4	0.00637
53	K	3	0.00478
53	P	3	0.00478
53	H	2	0.00318
53	W	1	0.00159
54	S	56599	89.98967
54	A	4346	6.90993
54	T	531	0.84426
54	G	413	0.65665
54	C	407	0.64711
54	P	209	0.3323
54	L	99	0.15741
54	F	91	0.14469
54	V	82	0.13038
54	N	38	0.06042
54	Y	32	0.05088
54	I	15	0.02385
54	W	9	0.01431
54	Q	8	0.01272
54	R	5	0.00795
54	M	4	0.00636
54	D	3	0.00477
54	E	2	0.00318
54	H	2	0.00318
55	S	22509	35.75638
55	T	10970	17.42625
55	A	10426	16.56209
55	G	7981	12.67811

55	R	2645	4.20168
55	V	1694	2.69098
55	I	1259	1.99997
55	D	789	1.25336
55	N	757	1.20252
55	Y	757	1.20252
55	L	685	1.08815
55	H	592	0.94041
55	F	534	0.84828
55	C	351	0.55758
55	E	316	0.50198
55	M	203	0.32247
55	Q	202	0.32088
55	K	138	0.21922
55	W	113	0.1795
55	P	30	0.04766
56	I	53478	84.87224
56	V	3204	5.08491
56	L	2062	3.2725
56	S	1442	2.28853
56	M	933	1.48072
56	T	807	1.28075
56	F	435	0.69037
56	A	138	0.21901
56	N	118	0.18727
56	C	102	0.16188
56	R	68	0.10792
56	G	60	0.09522
56	D	39	0.06189
56	K	39	0.06189
56	H	37	0.05872
56	E	29	0.04602
56	P	6	0.00952
56	Y	6	0.00952
56	Q	5	0.00794
56	W	2	0.00317
57	N	20544	32.58263
57	Y	14212	22.54013
57	S	9069	14.38337
57	D	4353	6.90383
57	T	2611	4.14103
57	K	2046	3.24494
57	H	1530	2.42657
57	R	1430	2.26797
57	G	1398	2.21722
57	A	1190	1.88733

57	V	973	1.54317
57	I	764	1.2117
57	F	641	1.01662
57	L	594	0.94208
57	E	581	0.92146
57	W	479	0.75969
57	P	244	0.38698
57	Q	146	0.23155
57	M	124	0.19666
57	C	123	0.19508
58	S	32769	51.91869
58	T	7240	11.47094
58	G	4330	6.86038
58	R	3363	5.32828
58	N	3011	4.77058
58	A	2479	3.92769
58	W	2001	3.17035
58	P	1464	2.31954
58	K	1112	1.76184
58	D	1073	1.70004
58	H	772	1.22314
58	I	768	1.21681
58	V	687	1.08847
58	E	521	0.82546
58	Y	440	0.69713
58	L	413	0.65435
58	F	310	0.49116
58	Q	163	0.25825
58	M	147	0.2329
58	C	53	0.08397
59	G	27847	44.05753
59	D	22172	35.07895
59	S	3873	6.12758
59	A	2105	3.33038
59	N	1592	2.51875
59	R	1149	1.81787
59	V	889	1.40651
59	T	718	1.13597
59	E	712	1.12648
59	L	593	0.9382
59	Y	469	0.74202
59	I	371	0.58697
59	H	239	0.37813
59	F	144	0.22783
59	P	135	0.21359
59	K	108	0.17087

59	W	38	0.06012
59	C	18	0.02848
59	Q	18	0.02848
59	M	16	0.02531
60	G	51017	80.5943
60	D	3423	5.4075
60	S	3384	5.34589
60	A	2777	4.38698
60	R	562	0.88782
60	V	360	0.56871
60	N	325	0.51342
60	E	301	0.47551
60	T	295	0.46603
60	I	217	0.34281
60	P	173	0.2733
60	Y	149	0.23538
60	H	91	0.14376
60	L	75	0.11848
60	F	52	0.08215
60	K	35	0.05529
60	C	26	0.04107
60	M	22	0.03475
60	Q	10	0.0158
60	W	7	0.01106
61	G	23552	37.1594
61	S	15420	24.32906
61	T	6238	9.84207
61	D	5672	8.94905
61	R	3393	5.35334
61	A	1996	3.14921
61	L	1354	2.13629
61	V	1173	1.85071
61	N	903	1.42472
61	E	833	1.31427
61	I	705	1.11232
61	K	538	0.84883
61	Y	453	0.71473
61	H	244	0.38497
61	P	220	0.34711
61	F	199	0.31397
61	M	173	0.27295
61	Q	155	0.24455
61	W	136	0.21458
61	C	24	0.03787
62	S	17058	26.88882
62	T	14214	22.40578

62	N	11351	17.89278
62	R	3624	5.71257
62	D	2824	4.45152
62	I	2698	4.2529
62	K	2169	3.41903
62	A	1958	3.08643
62	G	1673	2.63718
62	V	1216	1.9168
62	Y	1177	1.85533
62	L	820	1.29258
62	M	574	0.90481
62	P	497	0.78343
62	H	482	0.75978
62	E	455	0.71722
62	Q	362	0.57063
62	F	213	0.33576
62	W	49	0.07724
62	C	25	0.03941
63	T	47875	82.66853
63	A	2491	4.30135
63	I	1512	2.61086
63	L	1424	2.4589
63	S	1300	2.24479
63	P	963	1.66287
63	K	666	1.15002
63	V	404	0.69761
63	E	340	0.5871
63	R	298	0.51457
63	Q	239	0.4127
63	G	161	0.27801
63	M	99	0.17095
63	D	48	0.08288
63	Y	26	0.0449
63	W	23	0.03972
63	F	17	0.02935
63	N	13	0.02245
63	C	11	0.01899
63	H	2	0.00345
63	NA	0	0
64	NA	0	0
65	NA	0	0
66	Y	43004	67.57598
66	N	2552	4.01018
66	F	2357	3.70376
66	V	2207	3.46805
66	S	2061	3.23863

66	H	1828	2.8725
66	D	1789	2.81121
66	A	1368	2.14966
66	L	1254	1.97052
66	W	1011	1.58867
66	T	956	1.50225
66	R	816	1.28225
66	G	709	1.11411
66	I	649	1.01983
66	K	449	0.70555
66	E	223	0.35042
66	Q	162	0.25456
66	C	149	0.23414
66	P	49	0.077
66	M	45	0.07071
67	Y	59575	93.2039
67	S	1645	2.57357
67	F	471	0.73687
67	H	410	0.64144
67	T	342	0.53505
67	V	275	0.43023
67	D	248	0.38799
67	C	243	0.38017
67	L	149	0.23311
67	N	139	0.21746
67	Q	131	0.20495
67	A	128	0.20025
67	I	62	0.097
67	G	28	0.04381
67	E	24	0.03755
67	P	15	0.02347
67	W	15	0.02347
67	M	10	0.01564
67	R	7	0.01095
67	K	2	0.00313
68	A	50771	78.94606
68	S	3133	4.87164
68	T	3099	4.81877
68	V	1879	2.92174
68	P	1207	1.87682
68	G	1200	1.86593
68	L	720	1.11956
68	R	669	1.04026
68	E	581	0.90342
68	I	320	0.49758
68	Q	260	0.40429

68	D	172	0.26745
68	K	169	0.26279
68	H	53	0.08241
68	N	38	0.05909
68	M	24	0.03732
68	Y	7	0.01088
68	C	5	0.00777
68	F	2	0.00311
68	W	2	0.00311
69	D	58224	90.19426
69	E	1757	2.72175
69	N	1313	2.03396
69	G	822	1.27335
69	A	782	1.21139
69	P	395	0.61189
69	S	306	0.47402
69	H	261	0.40431
69	T	155	0.24011
69	V	154	0.23856
69	Q	92	0.14252
69	Y	90	0.13942
69	K	88	0.13632
69	R	58	0.08985
69	L	34	0.05267
69	I	12	0.01859
69	W	6	0.00929
69	F	3	0.00465
69	C	1	0.00155
69	M	1	0.00155
70	S	60601	92.99481
70	F	1856	2.84811
70	A	850	1.30436
70	P	603	0.92533
70	T	316	0.48492
70	H	272	0.4174
70	L	159	0.24399
70	V	101	0.15499
70	Y	97	0.14885
70	D	65	0.09975
70	G	58	0.089
70	N	54	0.08287
70	R	53	0.08133
70	K	18	0.02762
70	M	14	0.02148
70	W	14	0.02148
70	E	11	0.01688

70	C	10	0.01535
70	Q	9	0.01381
70	I	5	0.00767
71	V	59562	90.86915
71	M	2117	3.22974
71	L	1810	2.76138
71	A	1291	1.96958
71	I	195	0.2975
71	K	134	0.20443
71	E	131	0.19986
71	G	94	0.14341
71	T	93	0.14188
71	Q	20	0.03051
71	D	18	0.02746
71	F	18	0.02746
71	S	16	0.02441
71	W	14	0.02136
71	P	12	0.01831
71	H	9	0.01373
71	R	8	0.0122
71	Y	3	0.00458
71	C	1	0.00153
71	N	1	0.00153
72	K	60940	92.82276
72	R	1444	2.19948
72	E	1210	1.84305
72	Q	685	1.04338
72	T	338	0.51484
72	N	335	0.51027
72	M	189	0.28788
72	S	138	0.2102
72	A	129	0.19649
72	L	113	0.17212
72	V	73	0.11119
72	G	20	0.03046
72	D	14	0.02132
72	I	14	0.02132
72	W	4	0.00609
72	H	3	0.00457
72	P	2	0.00305
72	Y	1	0.00152
73	NA	0	0
74	G	63805	96.79744
74	D	1270	1.92669
74	A	298	0.45209
74	E	167	0.25335

74	V	126	0.19115
74	S	124	0.18812
74	C	31	0.04703
74	R	31	0.04703
74	T	17	0.02579
74	N	16	0.02427
74	H	13	0.01972
74	F	6	0.0091
74	P	5	0.00759
74	Y	3	0.00455
74	L	2	0.00303
74	W	2	0.00303
75	R	64375	97.50538
75	Q	1471	2.22805
75	L	73	0.11057
75	G	26	0.03938
75	H	20	0.03029
75	P	17	0.02575
75	K	16	0.02423
75	W	12	0.01818
75	C	7	0.0106
75	S	3	0.00454
75	M	1	0.00151
75	V	1	0.00151
76	F	63672	96.37927
76	L	767	1.161
76	S	515	0.77955
76	A	323	0.48892
76	V	252	0.38145
76	I	205	0.31031
76	Y	188	0.28457
76	C	87	0.13169
76	G	20	0.03027
76	W	15	0.02271
76	D	9	0.01362
76	P	3	0.00454
76	T	3	0.00454
76	N	2	0.00303
76	H	1	0.00151
76	Q	1	0.00151
76	R	1	0.00151
77	T	62389	94.3715
77	A	1655	2.5034
77	S	951	1.43851
77	I	700	1.05884
77	V	114	0.17244

77	P	79	0.1195
77	N	67	0.10135
77	F	65	0.09832
77	L	35	0.05294
77	D	22	0.03328
77	H	9	0.01361
77	R	8	0.0121
77	Q	5	0.00756
77	E	4	0.00605
77	K	2	0.00303
77	M	2	0.00303
77	Y	2	0.00303
77	C	1	0.00151
78	I	58277	88.11824
78	V	3897	5.89249
78	T	965	1.45914
78	M	847	1.28071
78	A	565	0.85431
78	L	545	0.82407
78	F	346	0.52317
78	S	210	0.31753
78	G	174	0.2631
78	C	173	0.26159
78	N	103	0.15574
78	D	17	0.0257
78	P	8	0.0121
78	H	3	0.00454
78	R	2	0.00302
78	Y	2	0.00302
78	K	1	0.00151
79	S	64297	97.18998
79	A	485	0.73312
79	T	439	0.66358
79	F	308	0.46557
79	P	298	0.45045
79	Y	95	0.1436
79	D	70	0.10581
79	N	34	0.05139
79	L	25	0.03779
79	V	25	0.03779
79	E	22	0.03325
79	G	15	0.02267
79	I	15	0.02267
79	C	12	0.01814
79	H	4	0.00605
79	R	4	0.00605

79	W	4	0.00605
79	M	2	0.00302
79	Q	2	0.00302
80	R	56836	85.8991
80	K	4126	6.23583
80	Q	2100	3.17384
80	G	628	0.94913
80	S	488	0.73754
80	L	333	0.50328
80	H	277	0.41864
80	E	259	0.39144
80	T	216	0.32645
80	V	210	0.31738
80	I	205	0.30983
80	A	171	0.25844
80	N	89	0.13451
80	M	60	0.09068
80	Y	60	0.09068
80	F	47	0.07103
80	W	46	0.06952
80	P	11	0.01662
80	D	3	0.00453
80	C	1	0.00151
81	D	63026	95.24572
81	A	811	1.22559
81	G	803	1.2135
81	N	675	1.02007
81	E	398	0.60146
81	I	174	0.26295
81	V	95	0.14357
81	H	83	0.12543
81	S	68	0.10276
81	Y	15	0.02267
81	T	12	0.01813
81	L	6	0.00907
81	P	2	0.00302
81	Q	2	0.00302
81	F	1	0.00151
81	W	1	0.00151
82	N	58949	89.08047
82	D	2477	3.74311
82	S	1713	2.58859
82	K	561	0.84775
82	T	430	0.64979
82	I	393	0.59388
82	V	305	0.4609

82	Y	265	0.40045
82	G	235	0.35512
82	H	225	0.34001
82	A	220	0.33245
82	R	179	0.27049
82	F	61	0.09218
82	L	46	0.06951
82	E	37	0.05591
82	M	33	0.04987
82	P	20	0.03022
82	Q	20	0.03022
82	C	3	0.00453
82	W	3	0.00453
83	A	57447	86.80811
83	T	3636	5.49436
83	V	1113	1.68185
83	D	1093	1.65163
83	S	960	1.45066
83	G	842	1.27235
83	P	286	0.43217
83	N	204	0.30826
83	L	148	0.22364
83	R	109	0.16471
83	I	86	0.12995
83	E	79	0.11938
83	H	67	0.10124
83	F	38	0.05742
83	Y	35	0.05289
83	K	12	0.01813
83	M	11	0.01662
83	Q	8	0.01209
83	W	3	0.00453
84	K	56737	85.72875
84	E	2529	3.82128
84	R	1444	2.18186
84	M	1126	1.70137
84	T	1087	1.64244
84	Q	925	1.39766
84	N	899	1.35838
84	A	336	0.50769
84	G	189	0.28558
84	Y	183	0.27651
84	D	176	0.26593
84	V	174	0.26291
84	L	170	0.25687
84	S	82	0.1239

84	W	40	0.06044
84	H	31	0.04684
84	P	28	0.04231
84	I	19	0.02871
84	F	7	0.01058
85	N	59612	90.07555
85	S	2230	3.3696
85	D	1097	1.6576
85	K	1034	1.56241
85	T	708	1.06981
85	R	320	0.48353
85	G	257	0.38833
85	Y	173	0.26141
85	A	161	0.24328
85	H	138	0.20852
85	I	138	0.20852
85	E	115	0.17377
85	M	58	0.08764
85	Q	54	0.0816
85	F	26	0.03929
85	L	24	0.03626
85	V	16	0.02418
85	P	15	0.02267
85	C	3	0.00453
85	W	1	0.00151
86	T	59070	89.25522
86	M	2961	4.47409
86	I	1237	1.86912
86	A	1149	1.73615
86	S	825	1.24658
86	L	246	0.37171
86	E	191	0.2886
86	V	181	0.27349
86	R	141	0.21305
86	K	90	0.13599
86	P	33	0.04986
86	N	26	0.03929
86	Q	13	0.01964
86	G	8	0.01209
86	F	5	0.00756
86	W	2	0.00302
86	Y	2	0.00302
86	D	1	0.00151
87	L	33074	49.97431
87	V	27600	41.70318
87	A	2031	3.06881

87	M	1942	2.93433
87	I	523	0.79025
87	T	219	0.33091
87	P	175	0.26442
87	G	137	0.207
87	F	117	0.17679
87	Q	102	0.15412
87	H	60	0.09066
87	W	60	0.09066
87	E	59	0.08915
87	S	30	0.04533
87	R	20	0.03022
87	D	9	0.0136
87	K	9	0.0136
87	Y	8	0.01209
87	N	5	0.00755
87	C	2	0.00302
88	Y	58477	88.3592
88	F	1734	2.62009
88	S	1383	2.08972
88	H	1064	1.60771
88	T	663	1.0018
88	D	646	0.97611
88	A	507	0.76608
88	N	429	0.64822
88	W	372	0.56209
88	L	311	0.46992
88	C	169	0.25536
88	I	169	0.25536
88	V	141	0.21305
88	R	61	0.09217
88	E	22	0.03324
88	M	14	0.02115
88	P	8	0.01209
88	Q	5	0.00756
88	G	4	0.00604
88	K	2	0.00302
89	L	65502	98.97403
89	P	286	0.43215
89	Q	115	0.17377
89	M	97	0.14657
89	V	76	0.11484
89	A	34	0.05137
89	F	25	0.03778
89	R	22	0.03324
89	S	10	0.01511

89	G	7	0.01058
89	I	4	0.00604
89	H	2	0.00302
89	T	1	0.00151
90	Q	60085	90.79164
90	E	1590	2.40257
90	L	1411	2.1321
90	H	1144	1.72865
90	R	1090	1.64705
90	K	357	0.53945
90	D	181	0.2735
90	T	87	0.13146
90	V	45	0.068
90	N	40	0.06044
90	S	36	0.0544
90	A	32	0.04835
90	Y	31	0.04684
90	G	18	0.0272
90	P	16	0.02418
90	I	9	0.0136
90	F	4	0.00604
90	M	2	0.00302
90	W	1	0.00151
91	M	47100	71.17061
91	L	16899	25.53529
91	I	873	1.31915
91	V	485	0.73286
91	T	400	0.60442
91	S	170	0.25688
91	A	110	0.16622
91	K	63	0.0952
91	F	43	0.06498
91	Q	15	0.02267
91	W	9	0.0136
91	R	7	0.01058
91	P	3	0.00453
91	C	1	0.00151
91	G	1	0.00151
92	N	55902	84.4709
92	D	4872	7.36185
92	S	2584	3.90456
92	T	977	1.4763
92	H	589	0.89001
92	I	273	0.41252
92	K	204	0.30825
92	Y	191	0.28861

92	G	139	0.21004
92	R	139	0.21004
92	A	98	0.14808
92	V	53	0.08009
92	L	44	0.06649
92	E	42	0.06346
92	F	36	0.0544
92	M	16	0.02418
92	Q	14	0.02115
92	P	5	0.00756
92	C	1	0.00151
93	S	56493	85.37426
93	N	4098	6.19305
93	G	1366	2.06435
93	T	1038	1.56866
93	D	917	1.3858
93	R	869	1.31326
93	A	484	0.73144
93	V	183	0.27656
93	M	154	0.23273
93	I	150	0.22669
93	K	104	0.15717
93	C	81	0.12241
93	L	59	0.08916
93	E	53	0.0801
93	Y	38	0.05743
93	Q	32	0.04836
93	H	29	0.04383
93	F	19	0.02871
93	P	2	0.00302
93	W	2	0.00302
94	L	64638	97.68328
94	P	520	0.78584
94	V	520	0.78584
94	M	127	0.19193
94	T	113	0.17077
94	A	105	0.15868
94	Q	103	0.15566
94	R	18	0.0272
94	I	7	0.01058
94	H	5	0.00756
94	S	5	0.00756
94	F	4	0.00604
94	C	3	0.00453
94	G	3	0.00453
95	K	56784	85.81403

95	R	2317	3.50153
95	E	2089	3.15697
95	Q	1656	2.50261
95	N	1317	1.9903
95	T	964	1.45683
95	S	337	0.50929
95	A	167	0.25238
95	I	135	0.20402
95	L	101	0.15263
95	V	93	0.14054
95	G	81	0.12241
95	D	60	0.09067
95	M	50	0.07556
95	H	13	0.01965
95	Y	4	0.00604
95	P	2	0.00302
95	W	1	0.00151
96	P	21572	32.60038
96	T	21303	32.19386
96	S	18757	28.34625
96	A	1176	1.77721
96	I	1020	1.54146
96	L	630	0.95208
96	F	453	0.68459
96	V	402	0.60752
96	Y	265	0.40048
96	R	228	0.34456
96	N	127	0.19193
96	D	62	0.0937
96	H	59	0.08916
96	E	42	0.06347
96	G	29	0.04383
96	M	20	0.03022
96	Q	19	0.02871
96	C	5	0.00756
96	K	2	0.00302
97	E	61400	92.78989
97	D	3381	5.10949
97	G	648	0.97928
97	A	224	0.33852
97	K	141	0.21308
97	V	114	0.17228
97	Q	113	0.17077
97	N	60	0.09067
97	T	55	0.08312
97	S	22	0.03325

97	H	3	0.00453
97	Y	3	0.00453
97	L	2	0.00302
97	W	2	0.00302
97	I	1	0.00151
97	P	1	0.00151
97	R	1	0.00151
98	D	65529	99.02979
98	G	235	0.35514
98	E	133	0.20099
98	Y	109	0.16472
98	N	79	0.11939
98	V	61	0.09219
98	H	14	0.02116
98	A	10	0.01511
98	T	1	0.00151
99	T	62753	94.8475
99	S	1629	2.46214
99	A	1200	1.81373
99	M	379	0.57284
99	N	41	0.06197
99	R	36	0.05441
99	I	27	0.04081
99	K	27	0.04081
99	P	27	0.04081
99	G	26	0.0393
99	L	6	0.00907
99	D	5	0.00756
99	E	5	0.00756
99	H	1	0.00151
100	A	62910	95.08623
100	G	2710	4.09607
100	S	183	0.2766
100	T	111	0.16777
100	V	104	0.15719
100	D	61	0.0922
100	F	44	0.0665
100	P	14	0.02116
100	Y	9	0.0136
100	C	5	0.00756
100	R	4	0.00605
100	N	3	0.00453
100	E	1	0.00151
100	H	1	0.00151
100	I	1	0.00151
101	V	24311	36.74521

101	M	18614	28.1344
101	L	16948	25.6163
101	I	1536	2.32161
101	Q	1221	1.8455
101	T	1143	1.7276
101	R	1057	1.59762
101	A	416	0.62877
101	K	374	0.56529
101	E	153	0.23125
101	P	132	0.19951
101	H	91	0.13754
101	G	46	0.06953
101	S	42	0.06348
101	D	29	0.04383
101	N	27	0.04081
101	F	13	0.01965
101	C	3	0.00453
101	W	3	0.00453
101	Y	2	0.00302
102	Y	65070	98.35397
102	H	445	0.67262
102	F	346	0.52298
102	C	193	0.29172
102	N	62	0.09371
102	S	12	0.01814
102	L	9	0.0136
102	D	7	0.01058
102	T	7	0.01058
102	V	6	0.00907
102	Q	1	0.00151
102	R	1	0.00151
103	Y	61883	93.60328
103	F	2237	3.38365
103	H	733	1.10872
103	S	347	0.52487
103	C	169	0.25563
103	W	169	0.25563
103	T	118	0.17848
103	L	96	0.14521
103	R	96	0.14521
103	I	86	0.13008
103	V	81	0.12252
103	N	65	0.09832
103	M	10	0.01513
103	A	9	0.01361
103	D	6	0.00908

103	Q	5	0.00756
103	K	2	0.00303
104	C	65964	99.82446
104	R	57	0.08626
104	Y	30	0.0454
104	S	19	0.02875
104	W	8	0.01211
104	F	2	0.00303
105	A	1015	93.37626
105	T	41	3.77185
105	V	25	2.29991
105	E	4	0.36799
105	R	1	0.092
105	S	1	0.092
106	A	123	40.19608
106	K	57	18.62745
106	R	55	17.97386
106	G	34	11.11111
106	S	8	2.61438
106	V	8	2.61438
106	I	7	2.28758
106	T	6	1.96078
106	Q	4	1.30719
106	E	3	0.98039
106	C	1	0.3268

Table S4: 1-VHH

The positions of amino acids	Amino acids	Counts	Percentage(%)
1	H	33972	67.90597
1	D	9673	19.33517
1	Q	4483	8.96098
1	E	771	1.54114
1	S	423	0.84553
1	M	225	0.44975
1	L	104	0.20788
1	P	91	0.1819
1	A	90	0.1799
1	Y	80	0.15991
1	R	27	0.05397
1	W	22	0.04398
1	G	19	0.03798
1	V	15	0.02998
1	N	13	0.02599
1	C	10	0.01999
1	I	4	0.008
1	T	3	0.006
1	K	2	0.004
1	F	1	0.002
2	V	49374	98.27432
2	C	407	0.8101
2	L	103	0.20501
2	M	87	0.17317
2	G	72	0.14331
2	W	61	0.12141
2	A	49	0.09753
2	H	27	0.05374
2	E	18	0.03583
2	Q	15	0.02986
2	S	10	0.0199
2	D	8	0.01592
2	R	6	0.01194
2	P	4	0.00796
3	Q	52971	98.66083
3	A	283	0.5271
3	S	112	0.2086
3	P	60	0.11175
3	V	47	0.08754
3	L	45	0.08381
3	C	43	0.08009
3	R	42	0.07823
3	E	25	0.04656

3	H	23	0.04284
3	K	22	0.04098
3	W	7	0.01304
3	G	5	0.00931
3	M	5	0.00931
4	L	53465	98.92133
4	A	205	0.37929
4	W	114	0.21092
4	Q	57	0.10546
4	P	56	0.10361
4	V	47	0.08696
4	M	41	0.07586
4	S	24	0.0444
4	R	22	0.0407
4	E	5	0.00925
4	H	4	0.0074
4	C	3	0.00555
4	D	2	0.0037
4	F	1	0.00185
4	K	1	0.00185
4	T	1	0.00185
5	V	54148	99.11407
5	G	169	0.30934
5	W	118	0.21599
5	L	57	0.10433
5	E	47	0.08603
5	A	42	0.07688
5	M	24	0.04393
5	S	8	0.01464
5	F	6	0.01098
5	T	5	0.00915
5	Q	4	0.00732
5	R	2	0.00366
5	C	1	0.00183
5	K	1	0.00183
6	E	54301	99.21071
6	G	146	0.26675
6	S	111	0.2028
6	A	59	0.1078
6	V	56	0.10231
6	K	23	0.04202
6	M	10	0.01827
6	W	9	0.01644
6	Q	7	0.01279
6	D	3	0.00548
6	Y	3	0.00548

6	R	2	0.00365
6	C	1	0.00183
6	F	1	0.00183
6	L	1	0.00183
7	S	54454	99.29613
7	L	106	0.19329
7	V	103	0.18782
7	F	50	0.09117
7	Y	37	0.06747
7	P	22	0.04012
7	E	18	0.03282
7	A	17	0.031
7	G	17	0.031
7	T	11	0.02006
7	C	3	0.00547
7	D	2	0.00365
8	G	54704	99.62847
8	W	108	0.19669
8	E	19	0.0346
8	S	19	0.0346
8	V	18	0.03278
8	R	16	0.02914
8	L	7	0.01275
8	D	5	0.00911
8	H	4	0.00728
8	A	3	0.00546
8	Q	3	0.00546
8	P	2	0.00364
9	G	54668	99.39636
9	E	104	0.18909
9	R	89	0.16182
9	V	71	0.12909
9	M	34	0.06182
9	A	15	0.02727
9	S	6	0.01091
9	D	5	0.00909
9	P	4	0.00727
9	W	4	0.00727
10	NA	0	0
11	G	51086	92.7841
11	D	2078	3.77413
11	A	978	1.77628
11	E	362	0.65748
11	S	195	0.35417
11	N	103	0.18707
11	R	84	0.15256

11	V	81	0.14711
11	K	18	0.03269
11	C	17	0.03088
11	Q	17	0.03088
11	T	17	0.03088
11	W	9	0.01635
11	F	3	0.00545
11	H	3	0.00545
11	I	2	0.00363
11	L	2	0.00363
11	Y	2	0.00363
11	M	1	0.00182
11	P	1	0.00182
12	S	50793	92.20506
12	L	2322	4.21515
12	T	557	1.01113
12	P	526	0.95485
12	A	451	0.8187
12	E	151	0.27411
12	V	83	0.15067
12	Q	43	0.07806
12	D	32	0.05809
12	W	29	0.05264
12	M	26	0.0472
12	H	23	0.04175
12	F	16	0.02904
12	R	11	0.01997
12	Y	6	0.01089
12	K	5	0.00908
12	N	5	0.00908
12	C	4	0.00726
12	G	3	0.00545
12	I	1	0.00182
13	V	52969	96.1133
13	A	1617	2.93408
13	M	143	0.25948
13	E	118	0.21411
13	G	114	0.20686
13	L	54	0.09798
13	I	43	0.07802
13	T	21	0.0381
13	K	14	0.0254
13	F	6	0.01089
13	R	4	0.00726
13	C	3	0.00544
13	S	3	0.00544

13	P	1	0.00181
13	W	1	0.00181
14	Q	50953	92.41833
14	E	1741	3.15782
14	R	854	1.54898
14	P	453	0.82165
14	K	287	0.52056
14	H	220	0.39904
14	L	214	0.38815
14	D	146	0.26481
14	T	66	0.11971
14	A	60	0.10883
14	S	48	0.08706
14	N	25	0.04534
14	M	20	0.03628
14	G	13	0.02358
14	C	12	0.02177
14	V	9	0.01632
14	Y	5	0.00907
14	W	4	0.00726
14	I	3	0.00544
15	A	39580	71.77051
15	T	4991	9.05019
15	P	4743	8.60049
15	V	3911	7.09183
15	S	885	1.60477
15	D	305	0.55306
15	I	158	0.2865
15	G	154	0.27925
15	E	131	0.23754
15	N	102	0.18496
15	H	78	0.14144
15	R	42	0.07616
15	F	32	0.05803
15	L	26	0.04715
15	Q	5	0.00907
15	C	3	0.00544
15	Y	2	0.00363
16	G	54798	99.29872
16	E	145	0.26275
16	R	119	0.21564
16	A	38	0.06886
16	V	25	0.0453
16	L	21	0.03805
16	W	15	0.02718
16	D	8	0.0145

16	S	8	0.0145
16	T	6	0.01087
16	M	1	0.00181
16	Q	1	0.00181
17	G	52047	94.27609
17	E	2018	3.65533
17	R	259	0.46914
17	D	208	0.37676
17	K	159	0.28801
17	Q	158	0.2862
17	S	99	0.17933
17	A	77	0.13948
17	V	59	0.10687
17	W	42	0.07608
17	T	40	0.07245
17	M	29	0.05253
17	N	9	0.0163
17	C	2	0.00362
17	L	1	0.00181
18	S	53882	97.53457
18	T	438	0.79285
18	A	300	0.54305
18	P	299	0.54124
18	F	145	0.26247
18	N	62	0.11223
18	Y	24	0.04344
18	H	20	0.0362
18	D	14	0.02534
18	L	12	0.02172
18	G	10	0.0181
18	C	8	0.01448
18	V	7	0.01267
18	E	6	0.01086
18	R	6	0.01086
18	I	3	0.00543
18	K	3	0.00543
18	W	3	0.00543
18	Q	2	0.00362
19	L	54302	98.23082
19	P	216	0.39074
19	V	187	0.33828
19	M	182	0.32923
19	R	141	0.25507
19	Q	131	0.23698
19	A	37	0.06693
19	T	30	0.05427

19	H	20	0.03618
19	S	16	0.02894
19	W	6	0.01085
19	I	5	0.00904
19	F	3	0.00543
19	C	2	0.00362
19	E	1	0.00181
19	G	1	0.00181
20	R	42144	76.21666
20	T	5816	10.51813
20	K	3678	6.6516
20	N	1444	2.61145
20	S	964	1.74338
20	G	296	0.53531
20	A	227	0.41053
20	I	191	0.34542
20	L	133	0.24053
20	V	130	0.2351
20	E	113	0.20436
20	Q	78	0.14106
20	M	51	0.09223
20	W	16	0.02894
20	D	5	0.00904
20	C	4	0.00723
20	H	4	0.00723
20	Y	1	0.00181
21	L	53769	97.13661
21	V	664	1.19955
21	I	481	0.86895
21	P	243	0.43899
21	H	77	0.1391
21	F	58	0.10478
21	G	22	0.03974
21	R	21	0.03794
21	A	8	0.01445
21	T	4	0.00723
21	S	3	0.00542
21	C	1	0.00181
21	E	1	0.00181
21	M	1	0.00181
21	Q	1	0.00181
22	S	52526	94.84823
22	T	1264	2.28245
22	A	1038	1.87436
22	P	220	0.39726
22	F	110	0.19863

22	V	41	0.07404
22	E	33	0.05959
22	Y	27	0.04875
22	R	26	0.04695
22	N	21	0.03792
22	G	16	0.02889
22	Q	16	0.02889
22	K	11	0.01986
22	I	7	0.01264
22	L	6	0.01083
22	C	5	0.00903
22	D	5	0.00903
22	H	4	0.00722
22	M	3	0.00542
23	C	54974	99.20598
23	R	233	0.42047
23	Y	81	0.14617
23	S	68	0.12271
23	W	22	0.0397
23	F	18	0.03248
23	G	10	0.01805
23	P	3	0.00541
23	T	2	0.00361
23	A	1	0.0018
23	L	1	0.0018
23	Q	1	0.0018
24	A	21646	39.05106
24	V	13346	24.07721
24	T	9856	17.78099
24	E	2614	4.71586
24	S	2305	4.1584
24	K	2034	3.66949
24	L	746	1.34584
24	Q	720	1.29894
24	I	523	0.94353
24	G	489	0.88219
24	R	474	0.85513
24	D	461	0.83168
24	P	93	0.16778
24	N	48	0.0866
24	H	31	0.05593
24	Y	18	0.03247
24	F	11	0.01984
24	M	10	0.01804
24	C	4	0.00722
24	W	1	0.0018

25	A	33961	61.24285
25	V	8504	15.33551
25	T	2533	4.56783
25	G	1842	3.32173
25	I	1719	3.09992
25	P	1377	2.48318
25	S	1052	1.8971
25	H	1024	1.84661
25	F	757	1.36512
25	L	709	1.27856
25	Y	532	0.95937
25	D	476	0.85838
25	R	384	0.69248
25	N	169	0.30476
25	M	152	0.27411
25	E	137	0.24706
25	Q	56	0.10099
25	W	32	0.05771
25	K	20	0.03607
25	C	17	0.03066
26	S	48637	87.68953
26	P	2354	4.24412
26	T	1602	2.88831
26	A	739	1.33237
26	F	377	0.67971
26	R	287	0.51744
26	H	258	0.46516
26	D	221	0.39845
26	Y	205	0.3696
26	G	127	0.22897
26	V	125	0.22537
26	N	121	0.21816
26	I	120	0.21635
26	L	116	0.20914
26	Q	62	0.11178
26	E	47	0.08474
26	K	37	0.06671
26	W	16	0.02885
26	C	7	0.01262
26	M	7	0.01262
27	G	43443	78.28837
27	E	2364	4.26015
27	R	1745	3.14465
27	A	1644	2.96264
27	V	1186	2.13728
27	F	928	1.67234

27	K	741	1.33535
27	D	721	1.29931
27	L	605	1.09027
27	T	572	1.0308
27	S	430	0.7749
27	P	280	0.50459
27	Q	253	0.45593
27	N	192	0.346
27	M	140	0.25229
27	I	134	0.24148
27	W	58	0.10452
27	Y	38	0.06848
27	H	15	0.02703
27	C	2	0.0036
28	Y	22772	41.03285
28	F	8272	14.90531
28	D	7041	12.68717
28	N	3436	6.19133
28	S	2651	4.77683
28	L	2076	3.74074
28	H	1594	2.87223
28	V	1434	2.58392
28	A	1100	1.98209
28	R	1061	1.91182
28	I	796	1.43431
28	G	616	1.10997
28	P	553	0.99645
28	T	511	0.92077
28	W	467	0.84149
28	K	406	0.73157
28	E	314	0.5658
28	C	141	0.25407
28	M	135	0.24326
28	Q	121	0.21803
29	T	31116	56.03458
29	I	5443	9.80191
29	S	4507	8.11633
29	P	2901	5.2242
29	A	2348	4.22835
29	R	1844	3.32073
29	N	1037	1.86746
29	G	1012	1.82244
29	D	909	1.63695
29	V	835	1.50369
29	L	776	1.39744
29	M	729	1.3128

29	F	541	0.97425
29	K	433	0.77976
29	Y	326	0.58707
29	E	278	0.50063
29	H	263	0.47362
29	Q	144	0.25932
29	W	72	0.12966
29	C	16	0.02881
30	Y	19514	35.13061
30	F	7561	13.6119
30	S	5089	9.16161
30	I	4208	7.57557
30	D	3268	5.88331
30	L	2261	4.07043
30	V	2135	3.84359
30	A	1862	3.35212
30	H	1860	3.34852
30	G	1714	3.08568
30	N	1510	2.71842
30	R	1179	2.12253
30	T	965	1.73727
30	P	604	1.08737
30	W	515	0.92714
30	K	358	0.6445
30	Q	305	0.54908
30	E	286	0.51488
30	M	197	0.35465
30	C	156	0.28084
31	S	23995	43.17511
31	G	5999	10.79423
31	D	5404	9.72362
31	R	4176	7.51403
31	N	4141	7.45106
31	T	2903	5.22348
31	A	1558	2.80337
31	E	1238	2.22758
31	K	1201	2.161
31	V	997	1.79394
31	I	831	1.49525
31	P	521	0.93746
31	C	513	0.92306
31	M	475	0.85469
31	H	437	0.78631
31	L	426	0.76652
31	Y	299	0.538
31	F	235	0.42284

31	Q	131	0.23571
31	W	96	0.17274
32	S	14406	25.90262
32	R	10800	19.41887
32	T	6903	12.4119
32	D	5712	10.27043
32	N	3941	7.08609
32	G	3079	5.53618
32	A	2223	3.99705
32	P	1010	1.81602
32	L	979	1.76028
32	V	920	1.6542
32	K	915	1.64521
32	I	848	1.52474
32	Y	775	1.39348
32	E	731	1.31437
32	H	612	1.1004
32	M	587	1.05545
32	Q	429	0.77136
32	F	404	0.72641
32	W	218	0.39197
32	C	124	0.22296
33	Y	24811	44.57039
33	N	6861	12.32508
33	S	3389	6.08799
33	H	3056	5.48979
33	C	2408	4.32572
33	F	2329	4.18381
33	A	2069	3.71674
33	G	1691	3.03771
33	R	1300	2.33532
33	L	1114	2.00119
33	D	1091	1.95987
33	T	1065	1.91316
33	K	963	1.72993
33	V	954	1.71376
33	I	843	1.51436
33	P	443	0.7958
33	W	381	0.68443
33	E	378	0.67904
33	M	292	0.52455
33	Q	229	0.41137
34	C	41297	74.0209
34	D	3985	7.14273
34	S	2088	3.74254
34	G	1672	2.9969

34	Y	1436	2.57389
34	A	1043	1.86948
34	F	801	1.43572
34	E	794	1.42317
34	R	482	0.86394
34	V	470	0.84243
34	L	324	0.58074
34	T	311	0.55744
34	N	261	0.46782
34	W	217	0.38895
34	H	196	0.35131
34	P	128	0.22943
34	I	107	0.19179
34	Q	84	0.15056
34	K	73	0.13085
34	M	22	0.03943
35	NA	0	0
36	NA	0	0
37	NA	0	0
38	NA	0	0
39	M	42532	75.98121
39	L	5621	10.04162
39	V	3588	6.40978
39	I	1674	2.99051
39	W	518	0.92538
39	T	463	0.82713
39	K	440	0.78604
39	R	351	0.62704
39	A	304	0.54308
39	F	166	0.29655
39	G	112	0.20008
39	Y	61	0.10897
39	Q	57	0.10183
39	S	42	0.07503
39	E	19	0.03394
39	C	13	0.02322
39	P	8	0.01429
39	N	5	0.00893
39	H	2	0.00357
39	D	1	0.00179
40	G	38866	68.75652
40	A	12571	22.23893
40	S	1362	2.40947
40	M	920	1.62754
40	D	882	1.56032
40	T	480	0.84915

40	V	294	0.52011
40	N	279	0.49357
40	E	195	0.34497
40	R	175	0.30959
40	H	118	0.20875
40	K	99	0.17514
40	I	94	0.16629
40	Y	67	0.11853
40	C	38	0.06722
40	F	31	0.05484
40	L	26	0.046
40	W	16	0.02831
40	Q	11	0.01946
40	P	3	0.00531
41	W	56312	99.02752
41	R	203	0.35699
41	Y	178	0.31302
41	F	64	0.11255
41	I	28	0.04924
41	L	22	0.03869
41	C	15	0.02638
41	G	15	0.02638
41	S	14	0.02462
41	A	3	0.00528
41	V	3	0.00528
41	H	2	0.00352
41	P	2	0.00352
41	Q	2	0.00352
41	M	1	0.00176
41	N	1	0.00176
42	F	45658	79.84262
42	Y	8160	14.26948
42	V	822	1.43744
42	L	798	1.39547
42	I	682	1.19262
42	S	418	0.73096
42	W	256	0.44767
42	H	137	0.23957
42	A	80	0.1399
42	C	53	0.09268
42	T	45	0.07869
42	N	23	0.04022
42	K	12	0.02098
42	M	9	0.01574
42	P	9	0.01574
42	D	7	0.01224

42	G	7	0.01224
42	Q	6	0.01049
42	R	3	0.00525
43	R	56743	98.79
43	H	272	0.47355
43	C	126	0.21937
43	S	76	0.13232
43	L	67	0.11665
43	Q	53	0.09227
43	I	27	0.04701
43	P	19	0.03308
43	G	15	0.02612
43	Y	15	0.02612
43	W	10	0.01741
43	K	6	0.01045
43	A	4	0.00696
43	F	3	0.00522
43	N	2	0.00348
44	Q	54674	95.02242
44	R	931	1.61806
44	E	787	1.36779
44	L	478	0.83076
44	K	211	0.36671
44	H	207	0.35976
44	P	39	0.06778
44	V	37	0.06431
44	G	32	0.05562
44	D	29	0.0504
44	A	28	0.04866
44	T	24	0.04171
44	S	22	0.03824
44	M	16	0.02781
44	F	12	0.02086
44	W	10	0.01738
44	C	1	0.00174
45	A	39290	68.1976
45	V	5084	8.82455
45	I	3279	5.69152
45	T	2946	5.11352
45	G	1965	3.41075
45	S	1714	2.97507
45	P	1434	2.48906
45	R	650	1.12824
45	D	321	0.55718
45	F	214	0.37145
45	Y	181	0.31417

45	L	156	0.27078
45	N	147	0.25516
45	H	88	0.15275
45	E	84	0.1458
45	K	29	0.05034
45	Q	25	0.04339
45	M	3	0.00521
45	C	2	0.00347
46	P	52697	91.31188
46	A	1607	2.78456
46	S	988	1.71198
46	L	435	0.75376
46	T	433	0.75029
46	E	408	0.70697
46	I	273	0.47305
46	Q	261	0.45225
46	V	208	0.36042
46	H	132	0.22873
46	D	121	0.20967
46	R	78	0.13516
46	G	27	0.04678
46	K	20	0.03466
46	F	9	0.01559
46	Y	7	0.01213
46	C	3	0.0052
46	N	3	0.0052
46	M	1	0.00173
47	G	52881	91.48804
47	D	3296	5.70232
47	E	913	1.57956
47	R	231	0.39965
47	A	125	0.21626
47	K	71	0.12284
47	V	45	0.07785
47	W	40	0.0692
47	N	37	0.06401
47	Q	36	0.06228
47	S	36	0.06228
47	T	33	0.05709
47	P	19	0.03287
47	M	18	0.03114
47	L	13	0.02249
47	Y	4	0.00692
47	C	3	0.00519
48	K	45927	79.36236
48	N	4517	7.80543

48	R	1716	2.96527
48	Q	1656	2.86159
48	E	1386	2.39502
48	S	448	0.77415
48	T	320	0.55296
48	D	309	0.53396
48	G	299	0.51668
48	M	296	0.51149
48	A	272	0.47002
48	H	176	0.30413
48	I	168	0.29031
48	L	138	0.23847
48	F	101	0.17453
48	V	51	0.08813
48	P	42	0.07258
48	Y	32	0.0553
48	W	16	0.02765
49	E	47496	81.9066
49	Q	2507	4.32331
49	A	2113	3.64386
49	G	1839	3.17135
49	K	1315	2.26771
49	D	1223	2.10906
49	R	399	0.68807
49	S	248	0.42767
49	V	238	0.41043
49	P	153	0.26385
49	T	133	0.22936
49	N	82	0.14141
49	L	75	0.12934
49	M	62	0.10692
49	Y	49	0.0845
49	H	27	0.04656
49	F	19	0.03277
49	W	9	0.01552
49	I	1	0.00172
50	R	51320	88.3548
50	C	5136	8.84237
50	L	464	0.79884
50	H	352	0.60602
50	P	257	0.44246
50	G	77	0.13257
50	S	77	0.13257
50	N	65	0.11191
50	Q	55	0.09469
50	V	48	0.08264

50	Y	36	0.06198
50	K	35	0.06026
50	T	33	0.05681
50	I	31	0.05337
50	F	29	0.04993
50	A	26	0.04476
50	D	25	0.04304
50	E	12	0.02066
50	W	5	0.00861
50	M	1	0.00172
51	E	55899	96.06455
51	K	566	0.97269
51	D	464	0.7974
51	V	341	0.58602
51	G	295	0.50697
51	Q	255	0.43823
51	A	126	0.21654
51	T	89	0.15295
51	L	45	0.07733
51	R	38	0.0653
51	I	21	0.03609
51	M	17	0.02922
51	N	15	0.02578
51	S	9	0.01547
51	W	4	0.00687
51	H	3	0.00516
51	P	2	0.00344
52	G	38244	65.66283
52	L	5558	9.54278
52	A	4601	7.89966
52	W	2590	4.44689
52	E	1573	2.70075
52	R	1334	2.2904
52	V	1117	1.91783
52	P	744	1.27741
52	F	725	1.24478
52	M	486	0.83444
52	K	373	0.64042
52	Q	280	0.48074
52	S	257	0.44125
52	T	112	0.1923
52	D	102	0.17513
52	I	67	0.11504
52	Y	28	0.04807
52	H	26	0.04464
52	C	13	0.02232

52	N	13	0.02232
53	V	51457	88.24579
53	I	4777	8.19228
53	L	1507	2.58442
53	A	273	0.46818
53	F	98	0.16806
53	D	89	0.15263
53	G	41	0.07031
53	T	20	0.0343
53	C	14	0.02401
53	E	8	0.01372
53	N	7	0.012
53	S	7	0.012
53	M	6	0.01029
53	P	4	0.00686
53	H	2	0.00343
53	Y	1	0.00171
54	A	49192	84.27184
54	S	7248	12.4167
54	G	770	1.3191
54	V	689	1.18034
54	T	313	0.53621
54	L	42	0.07195
54	P	37	0.06339
54	E	22	0.03769
54	I	20	0.03426
54	C	13	0.02227
54	W	9	0.01542
54	Y	5	0.00857
54	D	4	0.00685
54	M	3	0.00514
54	F	2	0.00343
54	Q	2	0.00343
54	R	2	0.00343
55	A	17760	30.38442
55	S	10312	17.64213
55	T	8867	15.16997
55	G	7038	12.04085
55	V	4803	8.21714
55	R	1877	3.21124
55	I	1480	2.53204
55	L	1386	2.37122
55	H	1029	1.76045
55	F	927	1.58594
55	N	517	0.8845
55	Y	508	0.8691

55	D	437	0.74763
55	C	351	0.6005
55	M	343	0.58682
55	Q	324	0.55431
55	E	200	0.34217
55	K	148	0.2532
55	W	102	0.17451
55	P	42	0.07186
56	I	44960	76.83631
56	L	4483	7.66141
56	V	3354	5.73196
56	F	1604	2.74122
56	M	1347	2.30201
56	T	1003	1.71412
56	S	421	0.71949
56	A	328	0.56055
56	R	286	0.48877
56	H	123	0.21021
56	Y	114	0.19483
56	K	102	0.17432
56	N	88	0.15039
56	D	77	0.13159
56	W	53	0.09058
56	G	39	0.06665
56	Q	37	0.06323
56	C	36	0.06152
56	E	31	0.05298
56	P	28	0.04785
57	D	19897	33.9777
57	Y	10800	18.44294
57	S	6236	10.64909
57	E	4104	7.00832
57	G	2576	4.39898
57	N	2256	3.85252
57	R	2240	3.8252
57	T	1868	3.18995
57	A	1831	3.12676
57	F	1345	2.29683
57	H	1236	2.11069
57	V	888	1.51642
57	W	888	1.51642
57	L	748	1.27734
57	C	422	0.72064
57	K	391	0.6677
57	I	379	0.64721
57	Q	277	0.47303

57	M	98	0.16735
57	P	79	0.13491
58	S	21781	37.14422
58	T	12765	21.76879
58	R	4847	8.26583
58	G	3750	6.39506
58	A	2447	4.17299
58	N	2389	4.07408
58	I	2010	3.42775
58	P	1802	3.07304
58	V	1314	2.24083
58	D	1180	2.01231
58	L	874	1.49048
58	F	756	1.28924
58	K	700	1.19374
58	H	517	0.88167
58	M	418	0.71284
58	Y	351	0.59858
58	E	278	0.47409
58	W	235	0.40076
58	Q	170	0.28991
58	C	55	0.09379
59	D	30620	52.13246
59	G	11600	19.74972
59	A	2266	3.85801
59	R	2244	3.82055
59	S	2098	3.57198
59	N	1819	3.09696
59	V	1420	2.41764
59	E	1330	2.26441
59	Y	1102	1.87622
59	T	1056	1.79791
59	I	827	1.40802
59	H	520	0.88533
59	P	449	0.76445
59	L	401	0.68273
59	F	348	0.59249
59	Q	246	0.41883
59	K	216	0.36775
59	M	78	0.1328
59	C	61	0.10386
59	W	34	0.05789
60	G	45385	77.13946
60	D	4532	7.7029
60	S	2157	3.66619
60	A	1140	1.93762

60	R	1076	1.82884
60	T	1020	1.73366
60	N	962	1.63508
60	E	529	0.89912
60	H	465	0.79035
60	V	365	0.62038
60	L	302	0.5133
60	I	228	0.38752
60	P	189	0.32124
60	Y	184	0.31274
60	F	138	0.23455
60	K	67	0.11388
60	M	48	0.08158
60	Q	23	0.03909
60	C	18	0.03059
60	W	7	0.0119
61	S	15881	26.9535
61	G	12978	22.02648
61	T	8040	13.64562
61	R	4757	8.07366
61	D	3676	6.23897
61	N	2500	4.24304
61	A	2142	3.63544
61	V	1702	2.88866
61	I	1617	2.7444
61	K	1218	2.06721
61	L	966	1.63951
61	E	787	1.33571
61	H	689	1.16938
61	P	597	1.01324
61	Y	417	0.70774
61	M	328	0.55669
61	F	292	0.49559
61	Q	234	0.39715
61	W	70	0.11881
61	C	29	0.04922
62	T	30906	52.37329
62	S	7458	12.63832
62	A	4071	6.89871
62	I	3357	5.68877
62	R	2273	3.85182
62	P	1650	2.79609
62	G	1631	2.76389
62	N	1527	2.58765
62	E	1167	1.9776
62	V	1033	1.75052

62	D	994	1.68443
62	K	881	1.49294
62	L	563	0.95406
62	M	340	0.57616
62	Y	312	0.52871
62	Q	279	0.47279
62	H	267	0.45246
62	F	246	0.41687
62	W	40	0.06778
62	C	16	0.02711
63	T	14435	71.6555
63	A	1340	6.65177
63	P	1223	6.07099
63	I	792	3.9315
63	S	662	3.28618
63	K	295	1.46438
63	R	287	1.42467
63	V	255	1.26582
63	Q	187	0.92827
63	L	181	0.89849
63	E	153	0.75949
63	Y	79	0.39216
63	F	56	0.27798
63	G	55	0.27302
63	D	46	0.22834
63	M	45	0.22338
63	N	33	0.16381
63	H	20	0.09928
63	W	1	0.00496
63	NA	0	0
64	NA	0	0
65	NA	0	0
66	Y	16603	28.08308
66	S	11669	19.73749
66	T	7238	12.24269
66	N	3405	5.75937
66	R	2628	4.44512
66	K	2385	4.0341
66	F	2360	3.99181
66	D	2089	3.53343
66	A	1922	3.25096
66	H	1646	2.78412
66	V	1539	2.60314
66	I	1195	2.02128
66	W	1083	1.83184
66	G	807	1.365

66	L	732	1.23814
66	E	703	1.18909
66	M	627	1.06054
66	Q	314	0.53111
66	C	159	0.26894
66	P	17	0.02875
67	Y	55645	93.6753
67	V	1128	1.89893
67	H	469	0.78954
67	F	447	0.7525
67	L	411	0.6919
67	I	332	0.5589
67	T	288	0.48483
67	S	203	0.34174
67	C	195	0.32827
67	N	77	0.12963
67	D	58	0.09764
67	A	45	0.07576
67	R	43	0.07239
67	G	17	0.02862
67	P	12	0.0202
67	Q	11	0.01852
67	M	10	0.01683
67	E	8	0.01347
67	W	3	0.00505
68	A	42080	70.41735
68	T	4205	7.03671
68	S	3279	5.48713
68	V	2196	3.67482
68	G	2132	3.56772
68	D	1308	2.18883
68	L	804	1.34543
68	I	731	1.22327
68	R	686	1.14796
68	E	671	1.12286
68	P	646	1.08103
68	K	372	0.62251
68	H	292	0.48864
68	N	131	0.21922
68	Q	108	0.18073
68	F	42	0.07028
68	M	38	0.06359
68	Y	30	0.0502
68	C	4	0.00669
68	W	3	0.00502
69	D	50751	84.47799

69	E	2893	4.81557
69	N	1605	2.67162
69	A	1347	2.24216
69	G	1188	1.9775
69	S	436	0.72575
69	P	400	0.66582
69	H	310	0.51601
69	V	303	0.50436
69	K	190	0.31627
69	T	182	0.30295
69	R	152	0.25301
69	Q	140	0.23304
69	Y	99	0.16479
69	L	36	0.05992
69	I	18	0.02996
69	F	10	0.01665
69	M	10	0.01665
69	C	4	0.00666
69	W	2	0.00333
70	S	53790	87.89216
70	A	3206	5.23856
70	F	1431	2.33824
70	P	1096	1.79085
70	T	588	0.96078
70	D	279	0.45588
70	Y	191	0.31209
70	V	110	0.17974
70	L	109	0.1781
70	N	107	0.17484
70	W	79	0.12908
70	I	61	0.09967
70	G	45	0.07353
70	H	41	0.06699
70	R	37	0.06046
70	Q	9	0.01471
70	C	7	0.01144
70	M	6	0.0098
70	K	5	0.00817
70	E	3	0.0049
71	V	58186	93.85747
71	A	2020	3.25838
71	M	526	0.84847
71	L	488	0.78717
71	I	295	0.47585
71	T	192	0.30971
71	E	129	0.20808

71	F	47	0.07581
71	G	43	0.06936
71	S	19	0.03065
71	W	12	0.01936
71	N	11	0.01774
71	P	9	0.01452
71	K	6	0.00968
71	D	5	0.00807
71	Q	4	0.00645
71	C	1	0.00161
71	R	1	0.00161
72	K	55207	88.73441
72	E	1863	2.99441
72	R	1713	2.75331
72	Q	1574	2.5299
72	N	485	0.77954
72	T	438	0.704
72	A	263	0.42272
72	M	256	0.41147
72	S	167	0.26842
72	L	92	0.14787
72	V	54	0.08679
72	G	34	0.05465
72	I	23	0.03697
72	D	22	0.03536
72	H	8	0.01286
72	Y	6	0.00964
72	P	5	0.00804
72	F	3	0.00482
72	W	2	0.00321
72	C	1	0.00161
73	NA	0	0
74	G	60075	95.8134
74	D	2009	3.20415
74	A	194	0.30941
74	S	169	0.26954
74	E	89	0.14195
74	V	64	0.10207
74	R	34	0.05423
74	C	24	0.03828
74	N	15	0.02392
74	L	8	0.01276
74	T	8	0.01276
74	H	3	0.00478
74	F	2	0.00319
74	K	2	0.00319

74	I	1	0.00159
74	P	1	0.00159
74	W	1	0.00159
74	Y	1	0.00159
75	R	62643	99.5835
75	Q	150	0.23845
75	L	56	0.08902
75	P	15	0.02385
75	H	11	0.01749
75	W	9	0.01431
75	C	8	0.01272
75	G	5	0.00795
75	S	4	0.00636
75	A	2	0.00318
75	E	1	0.00159
75	T	1	0.00159
76	F	61514	97.62577
76	L	399	0.63323
76	V	325	0.51579
76	S	321	0.50944
76	Y	147	0.2333
76	A	111	0.17616
76	I	107	0.16981
76	C	33	0.05237
76	T	18	0.02857
76	G	17	0.02698
76	W	11	0.01746
76	H	5	0.00794
76	D	1	0.00159
76	R	1	0.00159
77	T	56361	89.29607
77	A	2456	3.89119
77	I	1657	2.62528
77	S	1502	2.37971
77	N	398	0.63057
77	V	218	0.34539
77	F	161	0.25508
77	R	84	0.13309
77	L	63	0.09981
77	D	60	0.09506
77	K	49	0.07763
77	P	35	0.05545
77	M	24	0.03802
77	G	18	0.02852
77	H	18	0.02852
77	E	6	0.00951

77	C	2	0.00317
77	W	2	0.00317
77	Y	2	0.00317
77	Q	1	0.00158
78	I	54529	86.28143
78	V	5351	8.46691
78	L	1114	1.76269
78	T	570	0.90191
78	A	556	0.87976
78	M	479	0.75792
78	F	353	0.55855
78	S	70	0.11076
78	N	61	0.09652
78	G	44	0.06962
78	C	27	0.04272
78	D	15	0.02373
78	Y	14	0.02215
78	P	6	0.00949
78	Q	5	0.00791
78	R	2	0.00316
78	E	1	0.00158
78	H	1	0.00158
78	W	1	0.00158
79	S	60666	95.83432
79	T	905	1.42963
79	A	600	0.94782
79	P	339	0.53552
79	F	302	0.47707
79	Y	156	0.24643
79	D	97	0.15323
79	V	53	0.08372
79	I	35	0.05529
79	L	30	0.04739
79	R	30	0.04739
79	G	24	0.03791
79	N	23	0.03633
79	H	16	0.02528
79	W	10	0.0158
79	C	7	0.01106
79	E	5	0.0079
79	Q	5	0.0079
80	Q	25549	40.33055
80	R	15381	24.27978
80	K	10841	17.11314
80	H	4153	6.55575
80	L	2998	4.73251

80	E	1371	2.1642
80	V	624	0.98502
80	Y	424	0.66931
80	A	331	0.5225
80	T	307	0.48462
80	I	274	0.43252
80	S	260	0.41042
80	P	196	0.3094
80	G	167	0.26362
80	M	117	0.18469
80	F	109	0.17206
80	W	100	0.15786
80	N	95	0.14996
80	D	45	0.07104
80	C	7	0.01105
81	D	58271	91.91299
81	G	1477	2.32973
81	N	1432	2.25875
81	E	1044	1.64674
81	A	491	0.77447
81	V	235	0.37067
81	H	189	0.29812
81	S	125	0.19717
81	I	29	0.04574
81	T	28	0.04417
81	Y	23	0.03628
81	L	22	0.0347
81	P	18	0.02839
81	R	7	0.01104
81	K	3	0.00473
81	Q	3	0.00473
81	F	1	0.00158
82	N	38521	60.71558
82	S	6999	11.0316
82	K	4147	6.53637
82	D	3255	5.13043
82	A	2219	3.49752
82	T	1712	2.6984
82	R	1253	1.97494
82	G	1011	1.59351
82	I	993	1.56514
82	H	939	1.48002
82	Y	790	1.24517
82	V	717	1.13011
82	M	216	0.34045
82	L	196	0.30893

82	E	183	0.28844
82	F	122	0.19229
82	P	106	0.16707
82	Q	46	0.0725
82	W	14	0.02207
82	C	6	0.00946
83	A	48145	75.84159
83	D	3259	5.13382
83	T	3205	5.04875
83	S	3024	4.76363
83	V	1541	2.4275
83	G	1472	2.3188
83	P	545	0.85852
83	R	426	0.67107
83	N	422	0.66477
83	E	374	0.58915
83	L	356	0.5608
83	I	287	0.4521
83	H	105	0.1654
83	F	92	0.14493
83	Y	88	0.13862
83	K	60	0.09452
83	M	51	0.08034
83	Q	24	0.03781
83	W	4	0.0063
83	C	1	0.00158
84	K	50497	79.50155
84	E	2947	4.6397
84	N	2709	4.265
84	R	2056	3.23693
84	Q	1078	1.69718
84	T	965	1.51928
84	A	877	1.38073
84	G	546	0.85961
84	D	431	0.67856
84	S	398	0.6266
84	V	296	0.46602
84	M	214	0.33692
84	L	178	0.28024
84	W	139	0.21884
84	P	98	0.15429
84	H	33	0.05195
84	I	27	0.04251
84	Y	25	0.03936
84	F	3	0.00472
85	N	46604	73.35516

85	K	3387	5.33117
85	D	3327	5.23673
85	S	2490	3.91928
85	T	2145	3.37625
85	R	1543	2.4287
85	H	942	1.48272
85	G	677	1.0656
85	A	646	1.01681
85	Y	355	0.55877
85	I	265	0.41711
85	E	248	0.39035
85	M	220	0.34628
85	P	201	0.31638
85	Q	188	0.29591
85	V	124	0.19518
85	L	96	0.1511
85	F	71	0.11175
85	C	2	0.00315
85	W	1	0.00157
86	T	49179	77.38875
86	I	5435	8.55259
86	S	3380	5.31881
86	M	1772	2.78844
86	A	1703	2.67986
86	V	514	0.80884
86	N	369	0.58066
86	R	258	0.40599
86	E	210	0.33046
86	K	201	0.3163
86	L	186	0.29269
86	F	92	0.14477
86	P	75	0.11802
86	Q	68	0.10701
86	W	48	0.07553
86	G	23	0.03619
86	D	21	0.03305
86	H	8	0.01259
86	Y	6	0.00944
87	L	35847	56.404
87	V	19243	30.27819
87	I	2709	4.26252
87	M	2157	3.39396
87	A	1265	1.99043
87	W	667	1.0495
87	T	489	0.76942
87	F	430	0.67659

87	P	167	0.26277
87	Q	134	0.21084
87	G	103	0.16207
87	H	96	0.15105
87	S	90	0.14161
87	R	79	0.1243
87	E	31	0.04878
87	N	17	0.02675
87	Y	17	0.02675
87	C	5	0.00787
87	K	5	0.00787
87	D	3	0.00472
88	Y	49821	78.38297
88	D	3313	5.21232
88	F	3197	5.02981
88	S	1949	3.06635
88	H	1525	2.39927
88	T	1205	1.89582
88	N	675	1.06197
88	W	485	0.76305
88	I	356	0.56009
88	A	301	0.47356
88	L	211	0.33196
88	V	180	0.28319
88	C	138	0.21711
88	E	53	0.08338
88	R	50	0.07866
88	Q	28	0.04405
88	G	24	0.03776
88	K	24	0.03776
88	M	15	0.0236
88	P	11	0.01731
89	L	63056	99.18832
89	P	256	0.40269
89	Q	88	0.13843
89	M	74	0.1164
89	F	24	0.03775
89	V	18	0.02831
89	S	16	0.02517
89	I	14	0.02202
89	R	13	0.02045
89	H	12	0.01888
89	G	1	0.00157
90	Q	56197	88.40176
90	E	3378	5.31383
90	R	1286	2.02297

90	H	1160	1.82476
90	D	514	0.80856
90	L	324	0.50967
90	T	217	0.34136
90	N	133	0.20922
90	K	113	0.17776
90	V	61	0.09596
90	S	55	0.08652
90	I	39	0.06135
90	A	35	0.05506
90	G	19	0.02989
90	Y	15	0.0236
90	P	9	0.01416
90	M	7	0.01101
90	F	6	0.00944
90	W	2	0.00315
91	M	60048	94.45816
91	L	2235	3.51575
91	I	626	0.98473
91	T	311	0.48922
91	V	237	0.37281
91	K	53	0.08337
91	A	26	0.0409
91	S	12	0.01888
91	R	11	0.0173
91	F	3	0.00472
91	N	3	0.00472
91	G	2	0.00315
91	P	2	0.00315
91	Q	2	0.00315
92	N	46124	72.55167
92	D	8538	13.43002
92	S	3044	4.78812
92	T	2328	3.66187
92	H	1360	2.13924
92	R	364	0.57256
92	K	316	0.49706
92	E	280	0.44043
92	V	271	0.42627
92	A	236	0.37122
92	I	236	0.37122
92	G	211	0.3319
92	Y	154	0.24224
92	L	32	0.05034
92	Q	23	0.03618
92	F	22	0.03461

92	M	20	0.03146
92	P	8	0.01258
92	C	6	0.00944
92	W	1	0.00157
93	S	41293	64.9547
93	N	12267	19.29623
93	D	3373	5.3058
93	G	1752	2.75593
93	T	1254	1.97257
93	R	1111	1.74762
93	A	840	1.32134
93	I	310	0.48764
93	V	253	0.39797
93	K	241	0.3791
93	M	238	0.37438
93	E	186	0.29258
93	L	112	0.17618
93	H	95	0.14944
93	Y	86	0.13528
93	Q	60	0.09438
93	C	55	0.08652
93	F	24	0.03775
93	W	15	0.0236
93	P	7	0.01101
94	L	62059	97.61234
94	V	564	0.88711
94	A	270	0.42468
94	P	261	0.41053
94	M	217	0.34132
94	Q	102	0.16044
94	I	40	0.06292
94	T	24	0.03775
94	R	17	0.02674
94	S	12	0.01887
94	F	6	0.00944
94	K	3	0.00472
94	E	1	0.00157
94	H	1	0.00157
95	K	50064	78.74546
95	R	3379	5.31482
95	T	2431	3.82371
95	E	2330	3.66485
95	Q	2171	3.41476
95	N	1176	1.84973
95	S	541	0.85094
95	A	328	0.51591

95	I	305	0.47973
95	L	295	0.464
95	V	191	0.30042
95	G	125	0.19661
95	D	113	0.17774
95	M	80	0.12583
95	H	39	0.06134
95	P	5	0.00786
95	C	2	0.00315
95	F	1	0.00157
95	W	1	0.00157
96	P	52928	83.25023
96	T	3311	5.20786
96	S	2324	3.65541
96	L	1443	2.26969
96	A	1424	2.2398
96	V	905	1.42347
96	I	391	0.615
96	F	313	0.49232
96	R	202	0.31772
96	D	80	0.12583
96	H	79	0.12426
96	E	41	0.06449
96	N	38	0.05977
96	Y	31	0.04876
96	G	27	0.04247
96	Q	23	0.03618
96	M	15	0.02359
96	K	2	0.00315
97	E	54174	85.21006
97	D	7472	11.75268
97	G	684	1.07586
97	A	300	0.47187
97	N	199	0.31301
97	V	173	0.27211
97	S	164	0.25795
97	Q	143	0.22492
97	K	115	0.18088
97	T	95	0.14943
97	L	28	0.04404
97	W	10	0.01573
97	R	5	0.00786
97	Y	5	0.00786
97	H	3	0.00472
97	M	3	0.00472
97	F	2	0.00315

97	I	1	0.00157
97	P	1	0.00157
98	D	63070	99.20254
98	G	220	0.34604
98	E	85	0.1337
98	N	80	0.12583
98	V	72	0.11325
98	Y	20	0.03146
98	A	17	0.02674
98	H	13	0.02045
99	T	57256	90.08748
99	S	5107	8.03543
99	A	837	1.31695
99	G	67	0.10542
99	N	66	0.10385
99	I	57	0.08968
99	R	48	0.07552
99	Q	42	0.06608
99	M	39	0.06136
99	P	18	0.02832
99	D	7	0.01101
99	K	6	0.00944
99	V	3	0.00472
99	C	2	0.00315
99	L	1	0.00157
100	A	55998	88.10813
100	G	6838	10.75902
100	S	366	0.57587
100	D	132	0.20769
100	T	106	0.16678
100	V	84	0.13217
100	N	11	0.01731
100	P	7	0.01101
100	R	5	0.00787
100	E	4	0.00629
100	H	4	0.00629
100	C	1	0.00157
101	M	40771	64.14973
101	V	11512	18.11316
101	T	3970	6.24646
101	I	2678	4.21361
101	L	1987	3.12638
101	K	1056	1.66153
101	R	598	0.9409
101	A	239	0.37605
101	E	207	0.3257

101	N	121	0.19038
101	D	119	0.18724
101	S	119	0.18724
101	Q	90	0.14161
101	H	28	0.04406
101	W	18	0.02832
101	F	11	0.01731
101	G	11	0.01731
101	P	11	0.01731
101	Y	10	0.01573
102	Y	62940	99.03389
102	C	198	0.31155
102	H	196	0.3084
102	F	125	0.19668
102	N	65	0.10228
102	S	20	0.03147
102	D	4	0.00629
102	W	3	0.00472
102	L	2	0.00315
102	R	1	0.00157
103	Y	55892	88.1202
103	F	3227	5.08774
103	S	1308	2.06221
103	H	990	1.56085
103	T	482	0.75993
103	V	467	0.73628
103	I	334	0.52659
103	L	182	0.28694
103	R	177	0.27906
103	C	146	0.23019
103	N	89	0.14032
103	Q	58	0.09144
103	W	35	0.05518
103	A	12	0.01892
103	E	8	0.01261
103	K	8	0.01261
103	D	7	0.01104
103	M	3	0.00473
103	P	2	0.00315
104	C	63360	99.89752
104	R	37	0.05834
104	Y	12	0.01892
104	G	8	0.01261
104	S	7	0.01104
104	A	1	0.00158
105	A	7371	93.28018

105	V	209	2.6449
105	T	181	2.29056
105	G	56	0.70868
105	S	51	0.64541
105	E	21	0.26576
105	Q	9	0.1139
105	L	2	0.02531
105	K	1	0.01266
105	M	1	0.01266
106	A	6691	89.54764
106	T	212	2.83726
106	V	194	2.59636
106	S	120	1.606
106	P	110	1.47216
106	E	42	0.5621
106	G	41	0.54872
106	I	24	0.3212
106	K	14	0.18737
106	R	14	0.18737
106	L	8	0.10707
106	D	1	0.01338
106	Q	1	0.01338

Table S5: 2-VH

The positions of amino acids	Amino acids	Counts	Percentage(%)
1	H	30273	65.37315
1	D	9906	21.39155
1	Q	3775	8.15194
1	E	845	1.82474
1	S	725	1.5656
1	M	305	0.65863
1	L	161	0.34767
1	A	72	0.15548
1	P	67	0.14468
1	Y	57	0.12309
1	R	35	0.07558
1	N	23	0.04967
1	G	22	0.04751
1	V	16	0.03455
1	C	13	0.02807
1	W	6	0.01296
1	I	3	0.00648
1	K	3	0.00648
1	T	1	0.00216
2	V	45175	97.22162
2	C	722	1.55382
2	M	156	0.33573
2	G	89	0.19154
2	L	89	0.19154
2	W	87	0.18723
2	A	50	0.10761
2	H	24	0.05165
2	E	19	0.04089
2	S	17	0.03659
2	D	14	0.03013
2	R	14	0.03013
2	Q	8	0.01722
2	F	2	0.0043
3	Q	48039	97.68392
3	A	615	1.25056
3	S	157	0.31925
3	C	96	0.19521
3	P	76	0.15454
3	V	47	0.09557
3	R	42	0.0854
3	E	26	0.05287
3	K	26	0.05287
3	L	26	0.05287

3	H	15	0.0305
3	W	7	0.01423
3	G	6	0.0122
4	L	48483	98.00881
4	A	548	1.10779
4	W	150	0.30323
4	S	70	0.14151
4	Q	56	0.1132
4	P	48	0.09703
4	V	45	0.09097
4	M	34	0.06873
4	R	20	0.04043
4	G	7	0.01415
4	F	2	0.00404
4	T	2	0.00404
4	C	1	0.00202
4	D	1	0.00202
4	H	1	0.00202
5	V	48992	98.36961
5	G	493	0.98988
5	W	194	0.38953
5	L	35	0.07028
5	A	27	0.05421
5	E	24	0.04819
5	M	22	0.04417
5	F	7	0.01406
5	C	5	0.01004
5	P	1	0.00201
5	Q	1	0.00201
5	R	1	0.00201
5	S	1	0.00201
5	T	1	0.00201
6	E	49175	98.47211
6	G	435	0.87108
6	S	125	0.25031
6	M	57	0.11414
6	W	54	0.10813
6	A	31	0.06208
6	V	30	0.06007
6	K	14	0.02803
6	D	5	0.01001
6	Q	4	0.00801
6	R	3	0.00601
6	Y	3	0.00601
6	C	1	0.002
6	L	1	0.002

7	S	49281	98.49305
7	V	384	0.76746
7	L	114	0.22784
7	A	70	0.1399
7	F	60	0.11992
7	Y	49	0.09793
7	P	28	0.05596
7	G	14	0.02798
7	T	14	0.02798
7	W	7	0.01399
7	C	5	0.00999
7	E	5	0.00999
7	D	1	0.002
7	M	1	0.002
7	N	1	0.002
7	Q	1	0.002
8	G	49471	98.81552
8	W	398	0.79498
8	L	56	0.11186
8	R	31	0.06192
8	H	28	0.05593
8	D	22	0.04394
8	E	17	0.03396
8	S	12	0.02397
8	V	10	0.01997
8	A	8	0.01598
8	Q	7	0.01398
8	F	2	0.00399
8	P	1	0.002
8	T	1	0.002
9	G	49633	98.98686
9	E	234	0.46668
9	R	115	0.22935
9	V	85	0.16952
9	M	34	0.06781
9	S	14	0.02792
9	A	6	0.01197
9	C	6	0.01197
9	D	4	0.00798
9	W	4	0.00798
9	P	2	0.00399
9	F	1	0.00199
9	H	1	0.00199
9	K	1	0.00199
9	L	1	0.00199
10	NA	0	0

11	G	45494	90.62369
11	D	2408	4.79672
11	A	1041	2.07366
11	N	247	0.49202
11	R	216	0.43027
11	T	205	0.40836
11	S	161	0.32071
11	E	151	0.30079
11	V	101	0.20119
11	Q	66	0.13147
11	I	23	0.04582
11	C	18	0.03586
11	L	17	0.03386
11	Y	16	0.03187
11	H	15	0.02988
11	W	11	0.02191
11	K	6	0.01195
11	F	2	0.00398
11	M	2	0.00398
11	P	1	0.00199
12	L	41822	83.26929
12	S	6715	13.36984
12	V	347	0.69089
12	F	335	0.667
12	T	213	0.42409
12	W	186	0.37033
12	M	162	0.32255
12	A	124	0.24689
12	P	83	0.16526
12	Y	47	0.09358
12	R	46	0.09159
12	H	38	0.07566
12	Q	38	0.07566
12	E	19	0.03783
12	D	16	0.03186
12	K	14	0.02787
12	G	12	0.02389
12	I	5	0.00996
12	C	3	0.00597
13	V	48693	96.89571
13	A	803	1.59791
13	G	200	0.39799
13	M	192	0.38207
13	E	132	0.26267
13	L	79	0.1572
13	C	59	0.11741

13	I	39	0.07761
13	W	27	0.05373
13	R	8	0.01592
13	P	7	0.01393
13	T	5	0.00995
13	S	3	0.00597
13	N	2	0.00398
13	Q	2	0.00398
13	D	1	0.00199
13	F	1	0.00199
14	Q	47468	94.44301
14	R	1100	2.18858
14	H	361	0.71825
14	E	331	0.65856
14	K	229	0.45562
14	P	223	0.44368
14	L	191	0.38002
14	A	179	0.35614
14	S	60	0.11938
14	C	36	0.07163
14	T	18	0.03581
14	N	16	0.03183
14	G	9	0.01791
14	V	9	0.01791
14	D	8	0.01592
14	W	8	0.01592
14	Y	8	0.01592
14	M	4	0.00796
14	F	2	0.00398
14	I	1	0.00199
15	P	42098	83.74212
15	A	4959	9.86453
15	S	1915	3.80935
15	T	555	1.10402
15	F	230	0.45752
15	L	151	0.30037
15	V	150	0.29838
15	H	64	0.12731
15	G	31	0.06167
15	R	26	0.05172
15	D	25	0.04973
15	I	21	0.04177
15	E	20	0.03978
15	K	8	0.01591
15	Y	7	0.01392
15	Q	4	0.00796

15	N	3	0.00597
15	M	2	0.00398
15	C	1	0.00199
15	W	1	0.00199
16	G	49643	98.7115
16	E	195	0.38774
16	W	160	0.31815
16	R	125	0.24855
16	A	78	0.1551
16	V	42	0.08351
16	L	25	0.04971
16	D	5	0.00994
16	M	5	0.00994
16	T	4	0.00795
16	P	3	0.00597
16	S	3	0.00597
16	K	2	0.00398
16	C	1	0.00199
17	G	46615	92.64817
17	E	2452	4.8734
17	R	384	0.76321
17	K	223	0.44322
17	A	163	0.32397
17	V	139	0.27627
17	D	136	0.2703
17	W	72	0.1431
17	Q	53	0.10534
17	T	26	0.05168
17	S	22	0.04373
17	M	11	0.02186
17	N	7	0.01391
17	L	5	0.00994
17	C	3	0.00596
17	H	2	0.00398
17	I	1	0.00199
18	S	48911	97.08031
18	T	774	1.53626
18	P	256	0.50812
18	A	162	0.32154
18	F	126	0.25009
18	M	26	0.05161
18	V	23	0.04565
18	Y	23	0.04565
18	R	22	0.04367
18	L	18	0.03573
18	G	12	0.02382

18	N	9	0.01786
18	C	8	0.01588
18	I	6	0.01191
18	W	2	0.00397
18	D	1	0.00198
18	E	1	0.00198
18	H	1	0.00198
18	K	1	0.00198
19	L	49459	98.09596
19	P	259	0.5137
19	V	238	0.47204
19	Q	138	0.27371
19	R	113	0.22412
19	M	85	0.16859
19	A	58	0.11504
19	W	23	0.04562
19	T	17	0.03372
19	S	7	0.01388
19	G	5	0.00992
19	H	5	0.00992
19	K	5	0.00992
19	D	2	0.00397
19	E	2	0.00397
19	F	2	0.00397
19	I	1	0.00198
20	R	44359	87.93363
20	T	3125	6.19474
20	K	1476	2.9259
20	S	528	1.04666
20	G	378	0.74932
20	I	193	0.38259
20	N	131	0.25968
20	Q	64	0.12687
20	A	39	0.07731
20	E	39	0.07731
20	L	28	0.0555
20	M	25	0.04956
20	V	21	0.04163
20	H	14	0.02775
20	D	11	0.02181
20	C	7	0.01388
20	P	5	0.00991
20	W	2	0.00396
20	F	1	0.00198
21	L	47963	94.9424
21	V	1230	2.43478

21	I	797	1.57766
21	P	268	0.5305
21	H	100	0.19795
21	F	65	0.12867
21	R	41	0.08116
21	G	19	0.03761
21	A	15	0.02969
21	T	5	0.0099
21	S	4	0.00792
21	C	3	0.00594
21	Q	3	0.00594
21	D	2	0.00396
21	M	2	0.00396
21	W	1	0.00198
22	S	49009	96.92661
22	A	624	1.2341
22	T	458	0.9058
22	P	229	0.4529
22	F	66	0.13053
22	Q	32	0.06329
22	L	31	0.06131
22	Y	27	0.0534
22	V	25	0.04944
22	R	19	0.03758
22	D	10	0.01978
22	G	7	0.01384
22	I	7	0.01384
22	C	5	0.00989
22	N	5	0.00989
22	H	3	0.00593
22	E	2	0.00396
22	K	2	0.00396
22	W	2	0.00396
23	C	50112	98.94758
23	R	242	0.47784
23	Y	86	0.16981
23	S	70	0.13822
23	G	39	0.07701
23	W	33	0.06516
23	L	31	0.06121
23	F	19	0.03752
23	V	4	0.0079
23	A	3	0.00592
23	E	2	0.00395
23	P	2	0.00395
23	I	1	0.00197

23	M	1	0.00197
24	A	37809	74.63727
24	V	4398	8.68192
24	T	4057	8.00876
24	E	1582	3.12296
24	S	747	1.47462
24	K	415	0.81924
24	G	403	0.79555
24	Q	306	0.60406
24	L	303	0.59814
24	I	229	0.45206
24	R	207	0.40863
24	D	71	0.14016
24	P	69	0.13621
24	H	44	0.08686
24	N	11	0.02171
24	C	2	0.00395
24	F	2	0.00395
24	M	1	0.00197
24	Y	1	0.00197
25	A	42539	83.8835
25	V	3010	5.93548
25	T	2360	4.65373
25	G	1390	2.74097
25	S	295	0.58172
25	P	268	0.52847
25	I	237	0.46735
25	D	143	0.28198
25	F	107	0.211
25	L	89	0.1755
25	Y	77	0.15184
25	C	51	0.10057
25	R	41	0.08085
25	E	38	0.07493
25	H	26	0.05127
25	N	20	0.03944
25	M	13	0.02563
25	Q	4	0.00789
25	K	2	0.00394
25	W	2	0.00394
26	S	49022	96.58175
26	A	511	1.00676
26	P	401	0.79004
26	T	299	0.58908
26	F	158	0.31129
26	Y	101	0.19899

26	D	49	0.09654
26	L	39	0.07684
26	N	38	0.07487
26	H	28	0.05516
26	R	27	0.05319
26	I	23	0.04531
26	G	17	0.03349
26	E	14	0.02758
26	V	14	0.02758
26	C	10	0.0197
26	K	4	0.00788
26	Q	1	0.00197
26	W	1	0.00197
27	G	47908	94.34423
27	E	1014	1.99685
27	A	690	1.3588
27	R	615	1.21111
27	D	105	0.20677
27	V	99	0.19496
27	T	93	0.18314
27	K	71	0.13982
27	S	46	0.09059
27	L	32	0.06302
27	P	29	0.05711
27	I	28	0.05514
27	Q	22	0.04332
27	M	13	0.0256
27	N	5	0.00985
27	W	5	0.00985
27	C	2	0.00394
27	F	1	0.00197
27	H	1	0.00197
27	Y	1	0.00197
28	F	43334	85.283
28	L	3090	6.08124
28	Y	1189	2.34
28	S	723	1.42289
28	V	611	1.20247
28	I	495	0.97418
28	G	401	0.78918
28	D	195	0.38377
28	P	170	0.33457
28	A	160	0.31489
28	R	92	0.18106
28	N	86	0.16925
28	T	86	0.16925

28	H	55	0.10824
28	C	48	0.09447
28	M	36	0.07085
28	W	24	0.04723
28	E	10	0.01968
28	Q	6	0.01181
28	K	1	0.00197
29	T	39056	76.83047
29	A	2724	5.35862
29	S	2692	5.29567
29	I	1895	3.72782
29	P	1313	2.58292
29	R	673	1.32392
29	D	492	0.96786
29	N	366	0.71999
29	V	264	0.51934
29	G	244	0.47999
29	L	232	0.45639
29	M	199	0.39147
29	K	183	0.36
29	E	160	0.31475
29	Q	158	0.31082
29	F	104	0.20459
29	H	34	0.06688
29	Y	32	0.06295
29	W	8	0.01574
29	C	5	0.00984
30	F	44120	86.74282
30	L	1529	3.00611
30	V	1240	2.43792
30	Y	977	1.92085
30	S	886	1.74193
30	I	695	1.36642
30	A	386	0.7589
30	G	192	0.37748
30	T	158	0.31064
30	R	149	0.29294
30	D	127	0.24969
30	H	93	0.18284
30	N	79	0.15532
30	W	72	0.14156
30	M	48	0.09437
30	P	47	0.09241
30	C	33	0.06488
30	K	17	0.03342
30	E	15	0.02949

31	S	37539	73.78528
31	R	3149	6.18956
31	G	2495	4.90408
31	N	1955	3.84268
31	D	1827	3.59108
31	T	1451	2.85203
31	A	590	1.15968
31	K	509	1.00047
31	E	327	0.64274
31	V	289	0.56805
31	I	163	0.32039
31	H	128	0.25159
31	Y	113	0.22211
31	M	80	0.15725
31	C	72	0.14152
31	P	64	0.1258
31	F	46	0.09042
31	L	45	0.08845
31	Q	21	0.04128
31	W	13	0.02555
32	S	17874	35.11315
32	N	9170	18.0143
32	T	6279	12.33498
32	D	4728	9.28807
32	R	4122	8.0976
32	A	1800	3.53607
32	G	1528	3.00173
32	I	1157	2.27291
32	V	672	1.32013
32	K	585	1.14922
32	Y	585	1.14922
32	E	469	0.92134
32	L	468	0.91938
32	M	411	0.8074
32	H	344	0.67578
32	P	324	0.63649
32	F	152	0.2986
32	Q	104	0.20431
32	W	86	0.16895
32	C	46	0.09037
33	Y	34759	68.2459
33	H	2542	4.99097
33	N	2507	4.92225
33	S	2189	4.29789
33	F	1969	3.86594
33	A	1570	3.08254

33	V	1074	2.10869
33	T	1062	2.08513
33	D	755	1.48237
33	C	486	0.95421
33	R	363	0.71271
33	W	313	0.61454
33	G	254	0.4987
33	L	253	0.49674
33	I	249	0.48889
33	Q	204	0.40053
33	K	146	0.28666
33	M	102	0.20027
33	E	77	0.15118
33	P	58	0.11388
34	A	15872	31.13866
34	D	9274	18.1943
34	Y	6045	11.85945
34	G	5557	10.90206
34	W	4333	8.50075
34	S	1739	3.41168
34	T	1528	2.99772
34	V	1427	2.79958
34	C	1300	2.55042
34	P	946	1.85592
34	H	734	1.44001
34	F	597	1.17123
34	R	508	0.99663
34	E	349	0.68469
34	N	284	0.55717
34	I	186	0.36491
34	L	160	0.3139
34	Q	67	0.13144
34	M	51	0.10005
34	K	15	0.02943
35	NA	0	0
36	NA	0	0
37	NA	0	0
38	NA	0	0
39	M	44822	87.77269
39	I	2273	4.4511
39	V	1464	2.86688
39	L	1187	2.32444
39	T	495	0.96933
39	F	229	0.44844
39	A	188	0.36815
39	W	115	0.2252

39	K	96	0.18799
39	G	50	0.09791
39	R	46	0.09008
39	Q	38	0.07441
39	S	30	0.05875
39	P	17	0.03329
39	C	7	0.01371
39	H	3	0.00587
39	Y	3	0.00587
39	E	2	0.00392
39	N	1	0.00196
40	S	25554	49.91698
40	T	6246	12.20089
40	N	4782	9.34112
40	G	3303	6.45205
40	Y	3248	6.34462
40	A	2180	4.25839
40	H	1953	3.81497
40	D	1093	2.13506
40	I	751	1.467
40	M	591	1.15445
40	R	464	0.90637
40	V	352	0.68759
40	F	210	0.41021
40	C	151	0.29496
40	K	128	0.25003
40	L	88	0.1719
40	Q	59	0.11525
40	E	17	0.03321
40	W	16	0.03125
40	P	7	0.01367
41	W	50984	99.1463
41	R	263	0.51144
41	C	61	0.11862
41	L	35	0.06806
41	Y	27	0.05251
41	G	21	0.04084
41	S	11	0.02139
41	F	10	0.01945
41	V	4	0.00778
41	M	3	0.00583
41	E	1	0.00194
41	I	1	0.00194
41	P	1	0.00194
41	Q	1	0.00194
42	V	46329	89.62335

42	F	2639	5.10514
42	I	1121	2.16857
42	L	445	0.86085
42	A	440	0.85118
42	Y	408	0.78928
42	D	91	0.17604
42	G	66	0.12768
42	S	37	0.07158
42	T	27	0.05223
42	N	18	0.03482
42	W	15	0.02902
42	H	14	0.02708
42	M	12	0.02321
42	P	10	0.01934
42	R	10	0.01934
42	C	6	0.01161
42	Q	3	0.0058
42	E	2	0.00387
43	R	51407	99.04628
43	C	177	0.34103
43	H	156	0.30057
43	S	63	0.12138
43	L	24	0.04624
43	P	20	0.03853
43	G	18	0.03468
43	W	11	0.02119
43	F	6	0.01156
43	A	4	0.00771
43	Q	4	0.00771
43	T	4	0.00771
43	V	3	0.00578
43	N	2	0.00385
43	Y	2	0.00385
43	M	1	0.00193
44	Q	49758	95.65903
44	R	945	1.81675
44	L	669	1.28614
44	H	255	0.49023
44	K	135	0.25954
44	E	86	0.16533
44	M	32	0.06152
44	S	32	0.06152
44	P	27	0.05191
44	A	22	0.04229
44	W	22	0.04229
44	V	20	0.03845

44	D	5	0.00961
44	G	3	0.00577
44	T	3	0.00577
44	C	1	0.00192
44	F	1	0.00192
45	A	46700	89.66113
45	V	1758	3.37525
45	T	1143	2.19449
45	G	729	1.39964
45	P	514	0.98685
45	S	358	0.68734
45	D	332	0.63742
45	R	159	0.30527
45	I	135	0.25919
45	N	95	0.18239
45	L	46	0.08832
45	F	40	0.0768
45	Y	20	0.0384
45	C	19	0.03648
45	E	11	0.02112
45	K	11	0.02112
45	H	9	0.01728
45	M	3	0.00576
45	Q	3	0.00576
46	P	49979	95.81129
46	A	520	0.99686
46	S	480	0.92017
46	Q	456	0.87417
46	L	245	0.46967
46	R	166	0.31823
46	T	158	0.30289
46	K	64	0.12269
46	H	30	0.05751
46	V	19	0.03642
46	E	13	0.02492
46	G	11	0.02109
46	D	10	0.01917
46	M	6	0.0115
46	W	4	0.00767
46	C	2	0.00383
46	N	1	0.00192
47	G	51116	97.84652
47	E	611	1.16958
47	R	136	0.26033
47	V	122	0.23353
47	A	76	0.14548

47	D	44	0.08423
47	S	26	0.04977
47	N	24	0.04594
47	W	22	0.04211
47	T	18	0.03446
47	K	16	0.03063
47	Q	15	0.02871
47	L	6	0.01149
47	M	3	0.00574
47	H	2	0.00383
47	Y	2	0.00383
47	C	1	0.00191
47	P	1	0.00191
48	K	49771	95.20266
48	R	679	1.2988
48	E	542	1.03675
48	Q	425	0.81295
48	M	238	0.45525
48	N	196	0.37491
48	T	167	0.31944
48	A	73	0.13964
48	S	66	0.12625
48	L	43	0.08225
48	G	38	0.07269
48	V	15	0.02869
48	H	9	0.01722
48	D	6	0.01148
48	W	5	0.00956
48	C	2	0.00383
48	I	2	0.00383
48	P	2	0.00383
49	G	48337	92.34664
49	E	2658	5.07804
49	A	618	1.18067
49	R	230	0.43941
49	D	135	0.25791
49	V	104	0.19869
49	Q	62	0.11845
49	K	44	0.08406
49	W	33	0.06305
49	S	32	0.06114
49	P	30	0.05731
49	L	24	0.04585
49	T	13	0.02484
49	C	9	0.01719
49	N	9	0.01719

49	M	3	0.00573
49	H	1	0.00191
49	I	1	0.00191
50	L	47916	91.38868
50	R	2201	4.1979
50	F	838	1.59829
50	P	757	1.4438
50	V	192	0.3662
50	I	173	0.32996
50	H	100	0.19073
50	C	83	0.1583
50	M	51	0.09727
50	Q	39	0.07438
50	S	20	0.03815
50	K	15	0.02861
50	G	14	0.0267
50	A	7	0.01335
50	T	7	0.01335
50	Y	7	0.01335
50	D	5	0.00954
50	E	3	0.00572
50	W	3	0.00572
51	E	50865	96.90233
51	D	466	0.88777
51	K	366	0.69726
51	G	252	0.48008
51	Q	242	0.46103
51	V	104	0.19813
51	A	89	0.16955
51	N	22	0.04191
51	S	17	0.03239
51	W	16	0.03048
51	T	14	0.02667
51	R	12	0.02286
51	L	8	0.01524
51	M	6	0.01143
51	H	4	0.00762
51	Y	4	0.00762
51	C	3	0.00572
51	P	1	0.00191
52	W	48976	93.17581
52	G	1718	3.26846
52	R	549	1.04446
52	L	313	0.59548
52	C	292	0.55552
52	A	160	0.3044

52	Y	123	0.234
52	V	98	0.18644
52	S	83	0.15791
52	E	68	0.12937
52	F	62	0.11795
52	M	26	0.04946
52	H	23	0.04376
52	Q	18	0.03424
52	K	17	0.03234
52	P	12	0.02283
52	T	12	0.02283
52	D	10	0.01902
52	I	2	0.0038
52	N	1	0.0019
53	V	48135	91.49749
53	I	2806	5.33379
53	L	954	1.81341
53	A	264	0.50182
53	M	174	0.33075
53	D	80	0.15207
53	G	56	0.10645
53	T	34	0.06463
53	E	33	0.06273
53	F	33	0.06273
53	C	11	0.02091
53	S	10	0.01901
53	N	6	0.01141
53	Q	3	0.0057
53	R	3	0.0057
53	W	3	0.0057
53	H	2	0.0038
53	P	1	0.0019
54	S	46472	88.21899
54	A	4468	8.48172
54	T	600	1.139
54	G	301	0.5714
54	C	296	0.5619
54	L	170	0.32272
54	P	158	0.29994
54	I	74	0.14048
54	V	48	0.09112
54	F	27	0.05125
54	Q	26	0.04936
54	W	12	0.02278
54	M	9	0.01708
54	Y	9	0.01708

54	R	5	0.00949
54	E	2	0.0038
54	H	1	0.0019
55	S	11994	22.73485
55	A	9821	18.61589
55	T	9516	18.03776
55	G	9261	17.5544
55	V	2428	4.60232
55	R	1974	3.74175
55	C	1341	2.54189
55	D	1202	2.27841
55	N	1051	1.99219
55	H	901	1.70786
55	L	852	1.61498
55	I	546	1.03495
55	Y	542	1.02737
55	F	435	0.82455
55	Q	334	0.6331
55	E	225	0.42649
55	M	210	0.39806
55	K	54	0.10236
55	W	48	0.09098
55	P	21	0.03981
56	I	45050	85.30743
56	V	2686	5.08625
56	L	1272	2.40868
56	T	917	1.73645
56	M	860	1.62851
56	S	617	1.16836
56	F	546	1.03391
56	A	277	0.52453
56	N	107	0.20262
56	C	102	0.19315
56	D	93	0.17611
56	H	54	0.10226
56	R	49	0.09279
56	Y	42	0.07953
56	W	37	0.07006
56	G	32	0.0606
56	Q	25	0.04734
56	E	23	0.04355
56	K	12	0.02272
56	P	8	0.01515
57	N	20762	39.28551
57	S	7345	13.89809
57	Y	7304	13.82051

57	D	4256	8.05313
57	T	2648	5.0105
57	H	2174	4.11361
57	R	1248	2.36144
57	A	1239	2.34442
57	G	1022	1.93381
57	K	1005	1.90164
57	L	714	1.35102
57	V	711	1.34534
57	E	567	1.07287
57	I	550	1.0407
57	F	414	0.78336
57	W	353	0.66794
57	Q	163	0.30843
57	M	150	0.28383
57	P	114	0.21571
57	C	110	0.20814
58	S	25116	47.45852
58	T	5714	10.79702
58	G	4260	8.04958
58	R	3663	6.92151
58	N	2606	4.92423
58	A	2530	4.78062
58	P	1577	2.97986
58	W	1356	2.56226
58	K	1078	2.03696
58	I	920	1.73841
58	D	896	1.69306
58	V	609	1.15075
58	M	587	1.10918
58	Y	446	0.84275
58	H	370	0.69914
58	E	364	0.6878
58	L	329	0.62167
58	F	296	0.55931
58	Q	147	0.27777
58	C	58	0.1096
59	G	24189	45.64996
59	D	11380	21.47656
59	A	4334	8.17921
59	S	4175	7.87914
59	R	2024	3.81973
59	T	1266	2.38922
59	N	1208	2.27976
59	V	992	1.87212
59	E	729	1.37578

59	Y	598	1.12856
59	P	396	0.74734
59	F	394	0.74356
59	L	339	0.63977
59	I	286	0.53974
59	H	238	0.44916
59	W	159	0.30007
59	M	139	0.26232
59	K	75	0.14154
59	Q	54	0.10191
59	C	13	0.02453
60	G	42690	80.46367
60	S	3250	6.12572
60	D	3087	5.81849
60	A	1132	2.13363
60	T	576	1.08567
60	V	544	1.02535
60	N	389	0.7332
60	R	379	0.71435
60	E	278	0.52398
60	L	181	0.34116
60	Y	135	0.25445
60	P	110	0.20733
60	I	108	0.20356
60	H	56	0.10555
60	F	53	0.0999
60	C	34	0.06408
60	Q	15	0.02827
60	K	13	0.0245
60	M	13	0.0245
60	W	12	0.02262
61	G	21157	39.83019
61	S	11206	21.09643
61	D	5872	11.05463
61	T	4330	8.15166
61	R	2634	4.95877
61	A	1608	3.02722
61	N	1093	2.05768
61	V	1069	2.0125
61	I	965	1.81671
61	E	904	1.70187
61	L	374	0.70409
61	K	325	0.61185
61	Y	323	0.60808
61	Q	298	0.56102
61	H	290	0.54595

61	F	283	0.53278
61	M	166	0.31251
61	P	120	0.22591
61	W	62	0.11672
61	C	39	0.07342
62	S	17653	33.20043
62	T	11213	21.08856
62	N	5490	10.32518
62	R	4683	8.80743
62	I	2199	4.13571
62	G	1802	3.38907
62	A	1740	3.27246
62	K	1581	2.97343
62	D	1262	2.37347
62	V	1186	2.23054
62	Y	954	1.79421
62	M	951	1.78857
62	P	580	1.09082
62	L	560	1.05321
62	H	363	0.6827
62	E	347	0.65261
62	F	267	0.50215
62	W	161	0.3028
62	Q	141	0.26518
62	C	38	0.07147
63	T	38340	80.82128
63	A	2595	5.4703
63	I	1509	3.18099
63	S	1313	2.76782
63	P	1096	2.31038
63	K	598	1.26059
63	R	434	0.91488
63	E	329	0.69354
63	V	298	0.62819
63	Q	256	0.53965
63	L	238	0.50171
63	M	149	0.31409
63	G	78	0.16443
63	D	70	0.14756
63	N	67	0.14124
63	F	25	0.0527
63	Y	22	0.04638
63	W	9	0.01897
63	C	6	0.01265
63	H	6	0.01265
63	NA	0	0

64	NA	0	0
65	NA	0	0
66	Y	34791	65.23353
66	F	3096	5.80504
66	D	2778	5.20878
66	N	1952	3.66002
66	S	1646	3.08627
66	H	1574	2.95127
66	A	1288	2.41502
66	T	1073	2.01189
66	W	966	1.81126
66	V	853	1.59938
66	K	798	1.49626
66	R	697	1.30688
66	L	515	0.96563
66	G	444	0.83251
66	I	275	0.51563
66	E	200	0.375
66	Q	130	0.24375
66	C	129	0.24188
66	P	73	0.13688
66	M	55	0.10313
67	Y	49709	92.73202
67	S	1517	2.82996
67	V	352	0.65666
67	H	350	0.65292
67	F	341	0.63613
67	C	247	0.46078
67	T	211	0.39362
67	L	169	0.31527
67	D	156	0.29102
67	A	148	0.27609
67	N	128	0.23878
67	I	110	0.2052
67	E	57	0.10633
67	G	36	0.06716
67	K	16	0.02985
67	Q	15	0.02798
67	W	14	0.02612
67	M	12	0.02239
67	R	9	0.01679
67	P	8	0.01492
68	A	39815	73.82718
68	S	3708	6.87558
68	T	3095	5.73892
68	V	1937	3.59169

68	G	1403	2.60152
68	P	929	1.7226
68	R	652	1.20897
68	E	552	1.02355
68	L	546	1.01242
68	I	432	0.80104
68	K	287	0.53217
68	D	203	0.37641
68	Q	202	0.37456
68	H	61	0.11311
68	N	56	0.10384
68	M	34	0.06304
68	F	7	0.01298
68	Y	6	0.01113
68	C	4	0.00742
68	W	1	0.00185
69	D	46873	86.61739
69	E	2228	4.11716
69	N	1810	3.34473
69	G	1139	2.10478
69	A	793	1.4654
69	S	258	0.47676
69	V	218	0.40285
69	P	206	0.38067
69	H	172	0.31784
69	T	149	0.27534
69	R	85	0.15707
69	Y	70	0.12935
69	Q	46	0.085
69	K	38	0.07022
69	I	13	0.02402
69	L	7	0.01294
69	F	6	0.01109
69	M	2	0.0037
69	C	1	0.00185
69	W	1	0.00185
70	S	51318	93.86352
70	F	1067	1.9516
70	A	704	1.28766
70	P	562	1.02793
70	T	330	0.60359
70	Y	207	0.37861
70	V	102	0.18656
70	G	95	0.17376
70	D	88	0.16096
70	L	54	0.09877

70	H	45	0.08231
70	I	36	0.06585
70	N	26	0.04756
70	C	13	0.02378
70	R	12	0.02195
70	Q	5	0.00915
70	W	5	0.00915
70	E	2	0.00366
70	M	2	0.00366
71	V	51270	93.2504
71	M	1201	2.18439
71	A	1025	1.86428
71	L	936	1.70241
71	I	167	0.30374
71	E	111	0.20189
71	T	75	0.13641
71	G	66	0.12004
71	K	36	0.06548
71	R	25	0.04547
71	F	24	0.04365
71	P	20	0.03638
71	S	9	0.01637
71	W	6	0.01091
71	Q	4	0.00728
71	D	3	0.00546
71	N	3	0.00546
72	K	50540	91.81078
72	R	1776	3.22628
72	Q	814	1.47871
72	E	735	1.3352
72	N	278	0.50501
72	T	236	0.42872
72	M	198	0.35969
72	A	109	0.19801
72	L	105	0.19074
72	S	88	0.15986
72	V	66	0.1199
72	D	30	0.0545
72	G	25	0.04541
72	H	18	0.0327
72	W	11	0.01998
72	I	10	0.01817
72	P	5	0.00908
72	Y	4	0.00727
73	NA	0	0
74	G	53279	96.50769

74	D	1344	2.43447
74	A	236	0.42748
74	S	134	0.24272
74	E	64	0.11593
74	V	49	0.08876
74	C	42	0.07608
74	R	23	0.04166
74	N	14	0.02536
74	Q	8	0.01449
74	F	4	0.00725
74	T	4	0.00725
74	Y	3	0.00543
74	H	1	0.00181
74	K	1	0.00181
74	L	1	0.00181
75	R	54595	98.75014
75	Q	552	0.99844
75	L	56	0.10129
75	P	29	0.05245
75	G	19	0.03437
75	H	15	0.02713
75	W	7	0.01266
75	K	4	0.00724
75	C	3	0.00543
75	S	2	0.00362
75	V	2	0.00362
75	E	1	0.00181
75	T	1	0.00181
76	F	52797	95.44271
76	L	608	1.0991
76	C	580	1.04848
76	S	389	0.70321
76	V	371	0.67067
76	A	223	0.40312
76	Y	138	0.24947
76	I	125	0.22597
76	T	36	0.06508
76	G	20	0.03615
76	N	11	0.01989
76	M	7	0.01265
76	P	5	0.00904
76	H	3	0.00542
76	R	3	0.00542
76	D	1	0.00181
76	W	1	0.00181
77	T	51327	92.74008

77	A	2018	3.64622
77	I	827	1.49426
77	S	702	1.26841
77	N	122	0.22044
77	V	113	0.20417
77	F	57	0.10299
77	R	56	0.10118
77	P	33	0.05963
77	L	28	0.05059
77	D	23	0.04156
77	G	11	0.01988
77	K	10	0.01807
77	H	9	0.01626
77	Y	4	0.00723
77	E	2	0.00361
77	M	1	0.00181
77	Q	1	0.00181
77	W	1	0.00181
78	I	48122	86.92558
78	V	2860	5.16618
78	M	1841	3.32551
78	T	614	1.1091
78	A	546	0.98627
78	L	445	0.80383
78	S	271	0.48952
78	F	247	0.44617
78	C	173	0.3125
78	N	108	0.19509
78	G	107	0.19328
78	D	12	0.02168
78	H	6	0.01084
78	K	2	0.00361
78	R	2	0.00361
78	Y	2	0.00361
78	P	1	0.00181
78	Q	1	0.00181
79	S	52153	94.1917
79	A	1200	2.16728
79	F	662	1.19561
79	T	556	1.00417
79	Y	277	0.50028
79	P	250	0.45152
79	D	57	0.10295
79	G	44	0.07947
79	V	35	0.06321
79	W	31	0.05599

79	L	25	0.04515
79	C	18	0.03251
79	N	18	0.03251
79	E	13	0.02348
79	H	12	0.02167
79	I	10	0.01806
79	R	8	0.01445
80	R	46208	83.4456
80	Q	3505	6.32957
80	K	2568	4.63747
80	I	581	1.04921
80	E	568	1.02573
80	G	388	0.70068
80	L	328	0.59233
80	S	282	0.50926
80	H	232	0.41896
80	T	229	0.41354
80	A	200	0.36117
80	V	120	0.2167
80	P	42	0.07585
80	Y	35	0.06321
80	N	33	0.05959
80	M	32	0.05779
80	W	11	0.01986
80	F	8	0.01445
80	D	3	0.00542
80	C	2	0.00361
81	D	53198	96.06342
81	G	607	1.0961
81	N	503	0.9083
81	A	237	0.42797
81	E	221	0.39908
81	V	217	0.39185
81	Y	156	0.2817
81	S	77	0.13904
81	H	58	0.10473
81	T	31	0.05598
81	R	26	0.04695
81	W	20	0.03612
81	I	9	0.01625
81	F	6	0.01083
81	L	4	0.00722
81	Q	3	0.00542
81	K	2	0.00361
81	P	2	0.00361
81	C	1	0.00181

82	N	47480	85.73492
82	D	2573	4.64608
82	S	2046	3.69447
82	Y	546	0.98592
82	K	543	0.9805
82	T	521	0.94077
82	V	492	0.88841
82	I	353	0.63741
82	H	218	0.39364
82	A	215	0.38823
82	G	138	0.24919
82	R	122	0.2203
82	L	37	0.06681
82	M	33	0.05959
82	E	24	0.04334
82	F	24	0.04334
82	Q	7	0.01264
82	P	6	0.01083
82	W	2	0.00361
83	A	48452	87.48217
83	T	2252	4.06608
83	S	1105	1.99513
83	D	980	1.76943
83	G	839	1.51485
83	V	790	1.42638
83	P	248	0.44777
83	R	186	0.33583
83	N	157	0.28347
83	I	77	0.13903
83	Y	66	0.11917
83	F	59	0.10653
83	E	57	0.10292
83	L	51	0.09208
83	H	35	0.06319
83	Q	15	0.02708
83	M	11	0.01986
83	K	3	0.00542
83	W	2	0.00361
84	K	48491	87.55101
84	E	2513	4.53725
84	R	1350	2.43744
84	N	996	1.79829
84	Q	786	1.41913
84	A	323	0.58318
84	M	192	0.34666
84	G	144	0.25999

84	T	144	0.25999
84	L	140	0.25277
84	D	121	0.21847
84	V	77	0.13902
84	S	40	0.07222
84	H	21	0.03792
84	I	18	0.0325
84	W	15	0.02708
84	Y	9	0.01625
84	P	6	0.01083
85	N	49166	88.76973
85	S	2281	4.11837
85	D	1028	1.85606
85	K	1027	1.85426
85	T	528	0.95331
85	R	418	0.7547
85	Y	193	0.34846
85	G	162	0.29249
85	A	125	0.22569
85	H	125	0.22569
85	I	82	0.14805
85	L	75	0.13541
85	E	46	0.08305
85	M	38	0.06861
85	Q	31	0.05597
85	V	25	0.04514
85	F	15	0.02708
85	P	15	0.02708
85	C	5	0.00903
85	W	1	0.00181
86	T	47312	85.42231
86	M	4150	7.49287
86	A	1436	2.59271
86	I	665	1.20066
86	S	471	0.8504
86	V	428	0.77276
86	L	303	0.54707
86	E	205	0.37013
86	R	197	0.35569
86	K	127	0.2293
86	P	27	0.04875
86	Q	23	0.04153
86	N	19	0.0343
86	G	11	0.01986
86	H	8	0.01444
86	W	3	0.00542

86	F	1	0.00181
87	L	29880	53.9506
87	V	21664	39.11599
87	M	1577	2.84739
87	A	963	1.73877
87	I	419	0.75654
87	F	151	0.27264
87	Q	130	0.23472
87	T	122	0.22028
87	G	121	0.21847
87	P	117	0.21125
87	H	74	0.13361
87	W	67	0.12097
87	E	41	0.07403
87	S	28	0.05056
87	R	18	0.0325
87	N	6	0.01083
87	Y	3	0.00542
87	C	2	0.00361
87	D	1	0.00181
88	Y	48617	87.78642
88	F	1706	3.08048
88	S	1566	2.82768
88	H	809	1.46079
88	D	596	1.07618
88	T	583	1.05271
88	N	424	0.76561
88	W	282	0.5092
88	L	194	0.3503
88	I	151	0.27266
88	C	143	0.25821
88	A	136	0.24557
88	V	114	0.20585
88	R	18	0.0325
88	Q	12	0.02167
88	G	10	0.01806
88	E	9	0.01625
88	P	9	0.01625
88	M	2	0.00361
89	L	54614	98.61505
89	P	253	0.45684
89	V	175	0.31599
89	Q	146	0.26363
89	M	99	0.17876
89	R	32	0.05778
89	I	23	0.04153

89	S	18	0.0325
89	H	9	0.01625
89	F	7	0.01264
89	W	2	0.00361
89	A	1	0.00181
89	G	1	0.00181
89	T	1	0.00181
90	Q	50078	90.42288
90	E	1884	3.40183
90	H	1243	2.24441
90	R	1194	2.15594
90	L	401	0.72406
90	D	157	0.28349
90	T	95	0.17154
90	K	80	0.14445
90	S	60	0.10834
90	G	53	0.0957
90	V	34	0.06139
90	A	32	0.05778
90	I	26	0.04695
90	P	17	0.0307
90	N	14	0.02528
90	Y	7	0.01264
90	M	3	0.00542
90	F	2	0.00361
90	W	2	0.00361
91	M	32490	58.66633
91	L	21391	38.62516
91	I	597	1.07799
91	V	441	0.7963
91	T	173	0.31238
91	S	131	0.23654
91	F	62	0.11195
91	K	44	0.07945
91	A	21	0.03792
91	R	9	0.01625
91	W	7	0.01264
91	Q	6	0.01083
91	P	5	0.00903
91	G	3	0.00542
91	N	1	0.00181
92	N	46282	83.57018
92	D	3060	5.52536
92	S	2094	3.78108
92	T	1204	2.17403
92	I	726	1.31092

92	K	541	0.97687
92	H	344	0.62115
92	L	208	0.37558
92	Y	188	0.33947
92	V	167	0.30155
92	R	162	0.29252
92	A	114	0.20585
92	M	99	0.17876
92	G	97	0.17515
92	E	59	0.10653
92	F	24	0.04334
92	P	5	0.00903
92	C	3	0.00542
92	Q	2	0.00361
92	W	2	0.00361
93	S	45203	81.62186
93	N	4731	8.54264
93	G	1356	2.44849
93	D	1018	1.83818
93	R	789	1.42468
93	T	755	1.36328
93	V	411	0.74213
93	A	374	0.67532
93	M	141	0.2546
93	Y	115	0.20765
93	I	111	0.20043
93	K	91	0.16432
93	C	71	0.1282
93	L	66	0.11917
93	F	33	0.05959
93	E	30	0.05417
93	H	28	0.05056
93	W	28	0.05056
93	Q	21	0.03792
93	P	9	0.01625
94	L	53723	97.00619
94	V	849	1.53302
94	P	352	0.6356
94	M	199	0.35933
94	Q	108	0.19501
94	A	54	0.09751
94	R	36	0.065
94	T	22	0.03972
94	I	13	0.02347
94	F	8	0.01445
94	H	6	0.01083

94	S	4	0.00722
94	E	3	0.00542
94	G	3	0.00542
94	K	1	0.00181
95	K	45895	82.87138
95	E	2340	4.22528
95	R	2252	4.06638
95	Q	1395	2.51891
95	T	1270	2.29321
95	N	1085	1.95916
95	S	318	0.5742
95	I	246	0.4442
95	A	112	0.20224
95	G	108	0.19501
95	V	93	0.16793
95	H	66	0.11917
95	D	65	0.11737
95	M	62	0.11195
95	L	60	0.10834
95	Y	12	0.02167
95	P	2	0.00361
96	T	22922	41.38965
96	P	16842	30.41115
96	S	10199	18.41606
96	I	1912	3.45245
96	A	1646	2.97214
96	V	467	0.84325
96	L	369	0.66629
96	F	341	0.61573
96	R	140	0.25279
96	N	133	0.24015
96	G	111	0.20043
96	H	75	0.13543
96	D	70	0.1264
96	Y	67	0.12098
96	Q	33	0.05959
96	K	22	0.03972
96	E	13	0.02347
96	M	13	0.02347
96	C	3	0.00542
96	W	3	0.00542
97	E	49386	89.17499
97	D	4246	7.66689
97	G	806	1.45537
97	A	284	0.51281
97	K	133	0.24015

97	S	132	0.23835
97	V	131	0.23654
97	N	90	0.16251
97	Q	82	0.14807
97	W	35	0.0632
97	T	15	0.02709
97	R	12	0.02167
97	L	11	0.01986
97	M	7	0.01264
97	P	5	0.00903
97	I	3	0.00542
97	Y	3	0.00542
98	D	54862	99.06286
98	G	224	0.40447
98	N	87	0.15709
98	E	80	0.14445
98	V	72	0.13001
98	Y	23	0.04153
98	H	22	0.03972
98	A	10	0.01806
98	R	1	0.00181
99	T	44486	80.3446
99	M	8129	14.6815
99	S	1154	2.0842
99	A	1082	1.95416
99	V	131	0.23659
99	G	118	0.21312
99	I	57	0.10295
99	K	48	0.08669
99	L	45	0.08127
99	R	44	0.07947
99	Q	29	0.05238
99	P	17	0.0307
99	E	15	0.02709
99	W	10	0.01806
99	N	3	0.00542
99	D	1	0.00181
100	A	51421	92.87133
100	G	3266	5.89871
100	S	342	0.61769
100	V	129	0.23299
100	T	117	0.21131
100	D	60	0.10837
100	P	15	0.02709
100	N	8	0.01445
100	F	6	0.01084

100	C	2	0.00361
100	R	1	0.00181
100	Y	1	0.00181
101	M	27180	49.08973
101	V	13305	24.03013
101	L	8992	16.24043
101	I	1847	3.33586
101	T	1697	3.06495
101	R	883	1.59478
101	K	321	0.57976
101	A	245	0.44249
101	Q	242	0.43708
101	E	201	0.36303
101	S	113	0.20409
101	D	85	0.15352
101	H	75	0.13546
101	P	60	0.10837
101	W	47	0.08489
101	N	41	0.07405
101	G	26	0.04696
101	F	6	0.01084
101	C	1	0.00181
101	Y	1	0.00181
102	Y	54601	98.62007
102	F	261	0.47142
102	C	200	0.36124
102	H	189	0.34137
102	N	58	0.10476
102	I	20	0.03612
102	W	10	0.01806
102	D	9	0.01626
102	L	9	0.01626
102	S	5	0.00903
102	Q	2	0.00361
102	R	1	0.00181
103	Y	49334	89.12615
103	F	3577	6.46216
103	H	1181	2.13358
103	S	350	0.63231
103	W	175	0.31615
103	C	154	0.27821
103	I	102	0.18427
103	L	102	0.18427
103	R	93	0.16801
103	T	83	0.14995
103	V	73	0.13188

103	N	67	0.12104
103	K	21	0.03794
103	A	14	0.02529
103	M	9	0.01626
103	D	7	0.01265
103	E	6	0.01084
103	P	4	0.00723
103	Q	1	0.00181
104	C	55264	99.84643
104	R	47	0.08492
104	S	19	0.03433
104	Y	9	0.01626
104	W	6	0.01084
104	F	4	0.00723
105	A	1122	94.28571
105	T	27	2.26891
105	V	26	2.18487
105	G	7	0.58824
105	S	6	0.5042
105	D	1	0.08403
105	E	1	0.08403
106	A	289	67.52336
106	K	60	14.01869
106	R	27	6.30841
106	G	18	4.20561
106	V	11	2.57009
106	L	6	1.40187
106	E	5	1.16822
106	P	4	0.93458
106	I	2	0.46729
106	S	2	0.46729
106	T	2	0.46729
106	C	1	0.23364
106	Q	1	0.23364

Table S6: 2-VHH

The positions of amino acids	Amino acids	Counts	Percentage(%)
1	H	26226	67.51274
1	D	7668	19.73948
1	Q	3479	8.95588
1	E	645	1.6604
1	S	325	0.83664
1	M	151	0.38871
1	L	86	0.22139
1	P	79	0.20337
1	A	57	0.14673
1	Y	50	0.12871
1	N	22	0.05663
1	R	16	0.04119
1	G	10	0.02574
1	W	10	0.02574
1	V	8	0.02059
1	C	5	0.01287
1	K	3	0.00772
1	T	3	0.00772
1	I	2	0.00515
1	F	1	0.00257
2	V	38373	98.463
2	C	262	0.67228
2	M	88	0.2258
2	L	64	0.16422
2	W	47	0.1206
2	G	46	0.11803
2	A	43	0.11034
2	H	13	0.03336
2	E	12	0.03079
2	D	11	0.02823
2	S	7	0.01796
2	R	4	0.01026
2	F	1	0.00257
2	Q	1	0.00257
3	Q	40871	98.90857
3	A	195	0.4719
3	S	51	0.12342
3	P	47	0.11374
3	C	32	0.07744
3	R	30	0.0726
3	V	29	0.07018
3	L	21	0.05082
3	E	12	0.02904

3	H	11	0.02662
3	K	10	0.0242
3	W	7	0.01694
3	G	4	0.00968
3	M	1	0.00242
3	T	1	0.00242
4	L	41241	99.17516
4	A	146	0.3511
4	W	43	0.10341
4	Q	36	0.08657
4	P	28	0.06733
4	S	25	0.06012
4	M	24	0.05771
4	V	24	0.05771
4	R	9	0.02164
4	E	3	0.00721
4	C	2	0.00481
4	F	1	0.0024
4	G	1	0.0024
4	H	1	0.0024
5	V	41638	99.36759
5	G	125	0.29831
5	W	44	0.105
5	L	25	0.05966
5	A	23	0.05489
5	E	21	0.05012
5	M	16	0.03818
5	S	4	0.00955
5	T	3	0.00716
5	F	2	0.00477
5	R	2	0.00477
6	E	41764	99.43099
6	G	96	0.22856
6	A	41	0.09761
6	S	26	0.0619
6	V	23	0.05476
6	M	12	0.02857
6	W	11	0.02619
6	D	10	0.02381
6	K	9	0.02143
6	Q	7	0.01667
6	R	3	0.00714
6	C	1	0.00238
7	S	41877	99.55071
7	V	57	0.1355
7	F	30	0.07132

7	Y	29	0.06894
7	L	20	0.04754
7	A	19	0.04517
7	P	16	0.03804
7	T	7	0.01664
7	C	4	0.00951
7	G	4	0.00951
7	E	1	0.00238
7	R	1	0.00238
7	W	1	0.00238
8	G	41963	99.66985
8	W	68	0.16151
8	E	21	0.04988
8	R	15	0.03563
8	L	11	0.02613
8	V	8	0.019
8	D	7	0.01663
8	H	5	0.01188
8	C	2	0.00475
8	P	1	0.00238
8	Q	1	0.00238
9	G	41879	99.3382
9	E	69	0.16367
9	A	68	0.1613
9	R	65	0.15418
9	V	42	0.09963
9	M	19	0.04507
9	D	7	0.0166
9	S	3	0.00712
9	W	3	0.00712
9	L	1	0.00237
9	P	1	0.00237
9	Q	1	0.00237
10	NA	0	0
11	G	39389	93.34107
11	D	1678	3.9764
11	A	592	1.40288
11	E	118	0.27963
11	S	108	0.25593
11	R	101	0.23934
11	N	83	0.19669
11	T	46	0.10901
11	V	32	0.07583
11	C	22	0.05213
11	Q	17	0.04029
11	K	4	0.00948

11	W	3	0.00711
11	H	2	0.00474
11	L	2	0.00474
11	F	1	0.00237
11	Y	1	0.00237
12	S	38969	92.29111
12	L	1149	2.7212
12	T	781	1.84966
12	A	706	1.67203
12	P	348	0.82418
12	E	76	0.17999
12	V	54	0.12789
12	W	30	0.07105
12	M	22	0.0521
12	I	18	0.04263
12	Q	18	0.04263
12	F	17	0.04026
12	H	12	0.02842
12	D	9	0.02131
12	Y	5	0.01184
12	K	3	0.0071
12	R	3	0.0071
12	C	2	0.00474
12	G	2	0.00474
13	V	40763	96.49875
13	A	1085	2.56853
13	E	112	0.26514
13	M	112	0.26514
13	L	53	0.12547
13	I	48	0.11363
13	G	46	0.1089
13	Q	5	0.01184
13	R	5	0.01184
13	C	4	0.00947
13	D	3	0.0071
13	T	3	0.0071
13	P	2	0.00473
13	K	1	0.00237
14	Q	38468	91.01836
14	E	2009	4.75345
14	R	494	1.16884
14	P	286	0.6767
14	L	248	0.58679
14	H	216	0.51107
14	K	196	0.46375
14	S	85	0.20112

14	A	69	0.16326
14	N	49	0.11594
14	T	49	0.11594
14	D	28	0.06625
14	V	22	0.05205
14	M	18	0.04259
14	G	13	0.03076
14	W	6	0.0142
14	C	4	0.00946
14	F	4	0.00946
15	A	32863	77.72154
15	P	3678	8.69853
15	T	2929	6.92713
15	V	1210	2.86167
15	S	847	2.00317
15	D	289	0.68349
15	I	104	0.24596
15	G	103	0.2436
15	E	70	0.16555
15	N	51	0.12062
15	H	40	0.0946
15	R	38	0.08987
15	L	23	0.0544
15	F	20	0.0473
15	Q	8	0.01892
15	Y	8	0.01892
15	C	1	0.00237
15	K	1	0.00237
16	G	41894	99.03317
16	E	193	0.45623
16	R	77	0.18202
16	A	41	0.09692
16	T	35	0.08274
16	D	22	0.05201
16	V	21	0.04964
16	S	6	0.01418
16	W	5	0.01182
16	K	4	0.00946
16	Q	2	0.00473
16	H	1	0.00236
16	L	1	0.00236
16	N	1	0.00236
17	G	39992	94.48343
17	E	1410	3.33121
17	R	307	0.72531
17	D	167	0.39455

17	A	133	0.31422
17	V	68	0.16065
17	S	67	0.15829
17	Q	55	0.12994
17	T	37	0.08741
17	W	30	0.07088
17	N	26	0.06143
17	K	24	0.0567
17	M	6	0.01418
17	C	2	0.00473
17	Y	2	0.00473
17	I	1	0.00236
18	S	41144	97.1202
18	T	528	1.24634
18	A	237	0.55944
18	P	185	0.43669
18	F	100	0.23605
18	D	44	0.10386
18	N	28	0.06609
18	Y	25	0.05901
18	H	24	0.05665
18	G	16	0.03777
18	R	9	0.02124
18	V	7	0.01652
18	C	5	0.0118
18	M	4	0.00944
18	L	3	0.00708
18	I	2	0.00472
18	E	1	0.00236
18	Q	1	0.00236
18	W	1	0.00236
19	L	41469	97.84117
19	V	232	0.54738
19	R	179	0.42233
19	P	173	0.40817
19	M	172	0.40581
19	Q	80	0.18875
19	A	20	0.04719
19	H	18	0.04247
19	T	18	0.04247
19	S	6	0.01416
19	I	5	0.0118
19	F	4	0.00944
19	G	3	0.00708
19	K	2	0.00472
19	N	2	0.00472

19	E	1	0.00236
20	R	33566	79.15203
20	T	4261	10.04787
20	K	2694	6.35272
20	S	633	1.49268
20	N	400	0.94324
20	G	244	0.57538
20	I	204	0.48105
20	E	75	0.17686
20	M	69	0.16271
20	Q	63	0.14856
20	A	60	0.14149
20	L	45	0.10611
20	V	37	0.08725
20	W	18	0.04245
20	H	16	0.03773
20	P	13	0.03066
20	D	6	0.01415
20	C	2	0.00472
20	Y	1	0.00236
21	L	41515	97.76517
21	V	398	0.93726
21	I	214	0.50396
21	P	168	0.39563
21	H	65	0.15307
21	F	43	0.10126
21	G	27	0.06358
21	M	13	0.03061
21	R	7	0.01648
21	A	4	0.00942
21	Q	4	0.00942
21	S	2	0.00471
21	T	2	0.00471
21	D	1	0.00235
21	E	1	0.00235
22	S	39905	93.92506
22	T	1081	2.54437
22	A	976	2.29723
22	P	151	0.35541
22	V	78	0.18359
22	F	70	0.16476
22	L	44	0.10356
22	R	39	0.09179
22	N	35	0.08238
22	D	25	0.05884
22	Y	25	0.05884

22	G	17	0.04001
22	H	13	0.0306
22	I	8	0.01883
22	E	7	0.01648
22	Q	7	0.01648
22	C	2	0.00471
22	W	2	0.00471
22	K	1	0.00235
23	C	42169	99.16285
23	R	169	0.39741
23	S	82	0.19283
23	Y	47	0.11052
23	G	18	0.04233
23	W	16	0.03762
23	F	10	0.02352
23	L	9	0.02116
23	T	2	0.0047
23	H	1	0.00235
23	P	1	0.00235
23	V	1	0.00235
24	A	20821	48.96294
24	V	9333	21.94761
24	T	5761	13.54764
24	E	2109	4.95955
24	S	1320	3.10413
24	K	611	1.43684
24	Q	505	1.18756
24	L	466	1.09585
24	R	424	0.99708
24	G	415	0.97592
24	I	331	0.77838
24	D	193	0.45386
24	P	133	0.31276
24	N	40	0.09406
24	F	18	0.04233
24	H	18	0.04233
24	M	9	0.02116
24	Y	9	0.02116
24	C	7	0.01646
24	W	1	0.00235
25	A	26858	63.13439
25	V	6804	15.99398
25	T	1724	4.05256
25	G	1554	3.65295
25	S	1261	2.9642
25	I	1097	2.57869

25	F	667	1.5679
25	L	548	1.28817
25	P	494	1.16123
25	Y	427	1.00374
25	R	312	0.73341
25	M	216	0.50775
25	H	188	0.44193
25	D	154	0.362
25	W	61	0.14339
25	N	57	0.13399
25	K	44	0.10343
25	E	33	0.07757
25	Q	24	0.05642
25	C	18	0.04231
26	S	37989	89.26616
26	P	1341	3.15107
26	T	914	2.14771
26	A	474	1.1138
26	F	339	0.79658
26	H	266	0.62504
26	N	215	0.5052
26	R	180	0.42296
26	D	176	0.41356
26	Y	172	0.40416
26	L	154	0.36187
26	V	128	0.30077
26	G	71	0.16684
26	I	60	0.14099
26	Q	36	0.08459
26	E	25	0.05874
26	C	9	0.02115
26	K	6	0.0141
26	M	2	0.0047
27	G	32812	77.05784
27	E	2543	5.97215
27	R	1760	4.1333
27	A	1392	3.26906
27	T	726	1.70499
27	V	622	1.46075
27	D	528	1.23999
27	K	518	1.21651
27	S	370	0.86893
27	Q	360	0.84545
27	P	349	0.81961
27	L	190	0.44621
27	I	169	0.39689

27	F	59	0.13856
27	N	52	0.12212
27	M	40	0.09394
27	H	36	0.08454
27	Y	31	0.0728
27	W	20	0.04697
27	C	4	0.00939
28	Y	19282	45.2491
28	F	5277	12.38354
28	D	3678	8.63117
28	S	2617	6.14132
28	L	1869	4.38599
28	N	1855	4.35313
28	H	1456	3.4168
28	V	1182	2.7738
28	A	948	2.22467
28	I	899	2.10968
28	G	682	1.60045
28	R	570	1.33762
28	T	561	1.3165
28	W	541	1.26957
28	P	437	1.02551
28	E	281	0.65942
28	Q	175	0.41067
28	K	113	0.26518
28	C	100	0.23467
28	M	90	0.2112
29	T	23735	55.65586
29	I	4193	9.83211
29	S	3697	8.66904
29	A	2138	5.01337
29	P	1857	4.35445
29	R	1163	2.7271
29	L	1078	2.52779
29	V	923	2.16433
29	N	677	1.58749
29	D	635	1.489
29	G	524	1.22872
29	M	507	1.18886
29	F	351	0.82305
29	K	301	0.70581
29	Y	244	0.57215
29	Q	229	0.53698
29	H	194	0.45491
29	E	124	0.29077
29	W	71	0.16649

29	C	5	0.01172
30	Y	15000	35.15762
30	F	5955	13.95758
30	S	3709	8.69331
30	D	2616	6.13149
30	A	2432	5.70022
30	V	2078	4.8705
30	G	1724	4.04078
30	I	1416	3.31888
30	R	1221	2.86183
30	L	1214	2.84542
30	T	1116	2.61573
30	N	1023	2.39775
30	H	940	2.20321
30	W	699	1.63835
30	P	484	1.13442
30	E	404	0.94691
30	K	207	0.48518
30	Q	197	0.46174
30	M	141	0.33048
30	C	89	0.2086
31	S	19816	46.41728
31	R	4380	10.25977
31	N	3981	9.32515
31	G	3259	7.63393
31	T	2862	6.70399
31	D	2241	5.24935
31	A	1168	2.73594
31	K	881	2.06367
31	V	599	1.40311
31	I	568	1.33049
31	C	455	1.0658
31	E	420	0.98381
31	P	404	0.94634
31	Y	374	0.87606
31	M	325	0.76128
31	L	299	0.70038
31	H	257	0.602
31	F	157	0.36776
31	W	138	0.32325
31	Q	107	0.25064
32	S	10219	23.9164
32	T	6613	15.47697
32	R	6567	15.36931
32	N	3671	8.59156
32	G	2784	6.51563

32	D	2227	5.21204
32	I	1492	3.49186
32	A	1434	3.35611
32	P	1227	2.87165
32	L	1095	2.56272
32	V	890	2.08294
32	K	823	1.92614
32	M	723	1.6921
32	Y	722	1.68976
32	H	684	1.60082
32	F	564	1.31998
32	E	351	0.82148
32	W	304	0.71148
32	Q	289	0.67637
32	C	49	0.11468
33	Y	16614	38.86225
33	N	7262	16.98674
33	S	2151	5.03146
33	H	1774	4.14961
33	C	1623	3.7964
33	A	1620	3.78939
33	R	1600	3.7426
33	T	1264	2.95666
33	F	1251	2.92625
33	D	1243	2.90753
33	G	1239	2.89818
33	K	1119	2.61748
33	L	932	2.18007
33	V	744	1.74031
33	W	693	1.62101
33	I	555	1.29822
33	P	386	0.9029
33	Q	231	0.54034
33	M	227	0.53098
33	E	223	0.52163
34	C	30484	71.09639
34	S	2282	5.3222
34	Y	2216	5.16827
34	D	1542	3.59633
34	G	1320	3.07857
34	A	789	1.84015
34	R	737	1.71887
34	F	544	1.26875
34	T	544	1.26875
34	W	455	1.06117
34	V	444	1.03552

34	L	339	0.79063
34	N	279	0.6507
34	H	270	0.62971
34	I	228	0.53175
34	E	176	0.41048
34	P	96	0.2239
34	Q	62	0.1446
34	K	38	0.08863
34	M	32	0.07463
35	NA	0	0
36	NA	0	0
37	NA	0	0
38	NA	0	0
39	M	33998	78.94945
39	L	3366	7.81645
39	V	2520	5.85189
39	I	1656	3.84553
39	W	452	1.04962
39	T	260	0.60377
39	F	171	0.39709
39	R	157	0.36458
39	A	148	0.34368
39	K	138	0.32046
39	G	66	0.15326
39	Q	46	0.10682
39	S	26	0.06038
39	E	23	0.05341
39	C	12	0.02787
39	Y	12	0.02787
39	H	6	0.01393
39	P	6	0.01393
40	G	26909	61.67404
40	A	13411	30.73732
40	S	1058	2.42488
40	T	793	1.81752
40	V	376	0.86177
40	D	371	0.85031
40	H	148	0.33921
40	Y	136	0.3117
40	R	113	0.25899
40	N	107	0.24524
40	E	62	0.1421
40	L	30	0.06876
40	C	27	0.06188
40	Q	23	0.05271
40	K	21	0.04813

40	F	16	0.03667
40	I	10	0.02292
40	M	9	0.02063
40	W	8	0.01834
40	P	3	0.00688
41	W	43557	99.0382
41	R	200	0.45475
41	Y	99	0.2251
41	F	64	0.14552
41	L	22	0.05002
41	C	14	0.03183
41	S	9	0.02046
41	G	8	0.01819
41	V	3	0.00682
41	A	1	0.00227
41	E	1	0.00227
41	P	1	0.00227
41	Q	1	0.00227
42	F	37048	83.69025
42	Y	3736	8.4395
42	V	993	2.24316
42	L	921	2.08051
42	I	746	1.68519
42	W	237	0.53538
42	S	225	0.50827
42	H	160	0.36143
42	A	62	0.14006
42	C	29	0.06551
42	T	25	0.05647
42	N	23	0.05196
42	R	19	0.04292
42	D	13	0.02937
42	G	9	0.02033
42	P	7	0.01581
42	M	5	0.01129
42	Q	5	0.01129
42	E	3	0.00678
42	K	2	0.00452
43	R	44049	98.92427
43	H	234	0.52551
43	C	97	0.21784
43	S	32	0.07186
43	L	31	0.06962
43	Q	25	0.05614
43	P	22	0.04941
43	G	10	0.02246

43	F	9	0.02021
43	V	6	0.01347
43	Y	4	0.00898
43	A	2	0.00449
43	W	2	0.00449
43	D	1	0.00225
43	I	1	0.00225
43	K	1	0.00225
43	M	1	0.00225
43	T	1	0.00225
44	Q	42187	94.49223
44	R	1102	2.46831
44	E	372	0.83322
44	L	334	0.74811
44	H	198	0.44349
44	K	151	0.33822
44	D	65	0.14559
44	S	39	0.08735
44	A	36	0.08063
44	V	35	0.07839
44	P	31	0.06944
44	T	30	0.0672
44	M	26	0.05824
44	W	12	0.02688
44	G	11	0.02464
44	I	7	0.01568
44	Y	5	0.0112
44	N	3	0.00672
44	C	1	0.00224
44	F	1	0.00224
45	A	31568	70.57456
45	V	4343	9.70937
45	G	2036	4.55175
45	T	1690	3.77822
45	S	1534	3.42947
45	P	1115	2.49273
45	R	727	1.62531
45	I	425	0.95015
45	L	260	0.58127
45	D	235	0.52537
45	F	226	0.50525
45	Y	200	0.44713
45	N	181	0.40465
45	E	75	0.16767
45	H	49	0.10955
45	K	27	0.06036

45	Q	26	0.05813
45	C	6	0.01341
45	M	6	0.01341
45	W	1	0.00224
46	P	40614	90.60771
46	A	1266	2.82438
46	S	945	2.10825
46	L	545	1.21587
46	T	433	0.966
46	Q	388	0.86561
46	V	213	0.47519
46	R	138	0.30787
46	E	118	0.26325
46	D	51	0.11378
46	G	46	0.10262
46	K	18	0.04016
46	I	16	0.0357
46	H	15	0.03346
46	F	8	0.01785
46	N	5	0.01115
46	M	3	0.00669
46	W	1	0.00223
46	Y	1	0.00223
47	G	43033	95.79493
47	E	900	2.00347
47	D	230	0.512
47	A	204	0.45412
47	R	194	0.43186
47	T	81	0.18031
47	L	48	0.10685
47	S	48	0.10685
47	Q	37	0.08236
47	V	36	0.08014
47	P	35	0.07791
47	W	28	0.06233
47	K	16	0.03562
47	N	13	0.02894
47	M	8	0.01781
47	H	7	0.01558
47	Y	4	0.0089
48	K	36683	81.53046
48	N	2514	5.58754
48	Q	1645	3.65612
48	E	1230	2.73376
48	R	828	1.84029
48	S	410	0.91125

48	M	393	0.87347
48	T	277	0.61565
48	A	206	0.45785
48	D	199	0.44229
48	G	158	0.35117
48	L	154	0.34228
48	H	113	0.25115
48	P	69	0.15336
48	I	51	0.11335
48	V	37	0.08224
48	Y	15	0.03334
48	F	11	0.02445
49	E	37070	82.23706
49	G	1928	4.27713
49	Q	1787	3.96433
49	A	1197	2.65546
49	D	952	2.11194
49	K	902	2.00102
49	V	322	0.71433
49	R	279	0.61894
49	S	167	0.37048
49	P	166	0.36826
49	T	82	0.18191
49	L	69	0.15307
49	N	47	0.10427
49	M	46	0.10205
49	F	32	0.07099
49	W	12	0.02662
49	H	10	0.02218
49	Y	6	0.01331
49	I	3	0.00666
50	R	42036	93.04941
50	C	1687	3.73428
50	L	364	0.80574
50	H	304	0.67292
50	P	272	0.60209
50	S	126	0.27891
50	G	110	0.24349
50	V	50	0.11068
50	D	45	0.09961
50	A	33	0.07305
50	Y	29	0.06419
50	F	27	0.05977
50	Q	23	0.05091
50	T	17	0.03763
50	I	16	0.03542

50	N	13	0.02878
50	E	10	0.02214
50	K	9	0.01992
50	W	4	0.00885
50	M	1	0.00221
51	E	42720	94.40467
51	D	670	1.4806
51	V	474	1.04747
51	K	337	0.74472
51	Q	289	0.63865
51	G	270	0.59666
51	A	226	0.49943
51	R	90	0.19889
51	L	51	0.1127
51	M	26	0.05746
51	S	25	0.05525
51	I	24	0.05304
51	T	23	0.05083
51	N	11	0.02431
51	W	7	0.01547
51	Y	4	0.00884
51	F	2	0.00442
51	P	2	0.00442
51	H	1	0.00221
52	G	30935	68.30426
52	A	3846	8.49194
52	L	2323	5.12917
52	R	1677	3.7028
52	W	1506	3.32524
52	E	1466	3.23692
52	V	1329	2.93442
52	F	679	1.49923
52	M	338	0.7463
52	P	293	0.64694
52	K	238	0.5255
52	S	134	0.29587
52	T	126	0.27821
52	D	102	0.22522
52	I	84	0.18547
52	Q	79	0.17443
52	N	69	0.15235
52	H	41	0.09053
52	Y	15	0.03312
52	C	10	0.02208
53	V	40436	89.15052
53	I	3126	6.89199

53	L	1369	3.01828
53	A	150	0.33071
53	F	107	0.23591
53	D	66	0.14551
53	G	44	0.09701
53	C	14	0.03087
53	M	14	0.03087
53	T	12	0.02646
53	N	6	0.01323
53	P	3	0.00661
53	R	3	0.00661
53	S	3	0.00661
53	Y	2	0.00441
53	H	1	0.0022
53	K	1	0.0022
54	A	40182	88.46958
54	S	3512	7.73245
54	G	858	1.88908
54	V	448	0.98637
54	T	315	0.69354
54	P	28	0.06165
54	I	22	0.04844
54	L	18	0.03963
54	E	14	0.03082
54	C	4	0.00881
54	M	3	0.00661
54	Q	3	0.00661
54	W	3	0.00661
54	Y	3	0.00661
54	D	2	0.0044
54	F	2	0.0044
54	K	1	0.0022
54	R	1	0.0022
55	A	15061	33.11129
55	T	7376	16.21598
55	S	6872	15.10795
55	V	3778	8.30585
55	G	3209	7.05492
55	R	2544	5.59293
55	I	1264	2.77888
55	L	866	1.90388
55	H	820	1.80275
55	F	676	1.48617
55	C	617	1.35646
55	Y	471	1.03548
55	N	458	1.0069

55	D	359	0.78925
55	Q	280	0.61557
55	E	274	0.60238
55	M	274	0.60238
55	K	153	0.33637
55	W	109	0.23963
55	P	25	0.05496
56	I	34117	74.91985
56	L	3564	7.82643
56	V	2324	5.10343
56	M	1436	3.15341
56	F	1309	2.87452
56	T	790	1.73481
56	S	615	1.35052
56	A	459	1.00795
56	R	252	0.55338
56	Y	142	0.31183
56	K	138	0.30304
56	H	81	0.17787
56	D	61	0.13395
56	Q	54	0.11858
56	N	52	0.11419
56	W	45	0.09882
56	E	30	0.06588
56	G	24	0.0527
56	C	23	0.05051
56	P	22	0.04831
57	D	14695	32.2379
57	Y	8949	19.63232
57	S	4604	10.10026
57	G	2795	6.13167
57	N	2281	5.00406
57	A	2276	4.99309
57	E	1602	3.51447
57	T	1468	3.2205
57	H	1416	3.10642
57	R	1335	2.92872
57	F	951	2.0863
57	W	862	1.89106
57	V	586	1.28557
57	L	514	1.12761
57	Q	333	0.73054
57	C	301	0.66033
57	I	216	0.47386
57	K	192	0.42121
57	P	135	0.29616

57	M	72	0.15795
58	S	13336	29.20974
58	T	11558	25.3154
58	R	4247	9.30217
58	G	2560	5.60715
58	P	2288	5.01139
58	A	1967	4.30831
58	N	1899	4.15937
58	I	1487	3.25697
58	D	1157	2.53417
58	V	993	2.17496
58	Q	793	1.7369
58	L	695	1.52225
58	K	579	1.26818
58	H	430	0.94183
58	F	426	0.93306
58	Y	339	0.74251
58	M	320	0.70089
58	E	260	0.56948
58	W	226	0.49501
58	C	96	0.21027
59	D	18483	40.43093
59	G	11276	24.66586
59	A	2664	5.82741
59	R	2159	4.72274
59	S	2018	4.41431
59	N	1567	3.42776
59	E	1272	2.78246
59	Y	997	2.1809
59	V	961	2.10215
59	T	939	2.05403
59	H	843	1.84403
59	L	621	1.35842
59	P	500	1.09373
59	I	438	0.95811
59	F	409	0.89467
59	Q	241	0.52718
59	K	178	0.38937
59	W	76	0.16625
59	M	40	0.0875
59	C	33	0.07219
60	G	31151	68.03162
60	D	4940	10.78862
60	S	2726	5.95339
60	T	1575	3.43969
60	A	1515	3.30865

60	N	894	1.95243
60	R	777	1.69691
60	E	427	0.93254
60	L	383	0.83645
60	V	337	0.73598
60	F	202	0.44115
60	Y	188	0.41058
60	H	186	0.40621
60	I	164	0.35816
60	P	151	0.32977
60	Q	65	0.14196
60	K	49	0.10701
60	W	22	0.04805
60	C	20	0.04368
60	M	17	0.03713
61	G	12393	27.01236
61	S	9165	19.97646
61	T	6559	14.2963
61	R	4621	10.07215
61	D	2499	5.44694
61	A	1833	3.99529
61	N	1810	3.94516
61	V	1214	2.64609
61	I	1209	2.63519
61	E	908	1.97912
61	K	706	1.53883
61	L	631	1.37536
61	F	434	0.94597
61	P	415	0.90455
61	M	410	0.89366
61	Y	385	0.83916
61	H	325	0.70839
61	Q	246	0.53619
61	W	93	0.20271
61	C	23	0.05013
62	T	22145	48.19893
62	S	6024	13.11133
62	R	2876	6.25966
62	A	2836	6.1726
62	I	2522	5.48917
62	P	1689	3.67613
62	N	1509	3.28436
62	K	1084	2.35934
62	V	1060	2.30711
62	G	1038	2.25922
62	D	722	1.57144

62	L	546	1.18838
62	E	470	1.02296
62	M	398	0.86625
62	Y	312	0.67907
62	H	224	0.48754
62	F	213	0.4636
62	Q	178	0.38742
62	W	82	0.17847
62	C	17	0.037
63	T	13547	71.8407
63	A	1369	7.2599
63	P	1093	5.79626
63	S	698	3.70154
63	I	643	3.40987
63	K	283	1.50077
63	R	278	1.47425
63	L	255	1.35228
63	V	183	0.97046
63	E	164	0.8697
63	Q	111	0.58864
63	G	50	0.26515
63	M	44	0.23334
63	N	34	0.1803
63	F	32	0.1697
63	D	31	0.1644
63	W	23	0.12197
63	Y	11	0.05833
63	H	5	0.02652
63	C	3	0.01591
63	NA	0	0
64	NA	0	0
65	NA	0	0
66	Y	11645	25.27566
66	T	7190	15.60601
66	S	6726	14.59889
66	R	3094	6.71558
66	N	2563	5.56303
66	D	1886	4.09359
66	F	1870	4.05886
66	A	1772	3.84615
66	H	1427	3.09733
66	V	1408	3.05609
66	K	1174	2.54819
66	L	1025	2.22478
66	W	1007	2.18571
66	I	949	2.05982

66	E	737	1.59967
66	G	562	1.21983
66	M	457	0.99193
66	Q	356	0.7727
66	C	182	0.39503
66	P	42	0.09116
67	Y	43065	92.92265
67	V	783	1.6895
67	H	464	1.00119
67	F	427	0.92135
67	I	425	0.91704
67	S	264	0.56964
67	L	258	0.55669
67	C	180	0.38839
67	D	131	0.28266
67	T	129	0.27835
67	N	85	0.18341
67	A	56	0.12083
67	W	20	0.04315
67	P	12	0.02589
67	E	11	0.02374
67	R	10	0.02158
67	K	9	0.01942
67	G	8	0.01726
67	M	5	0.01079
67	Q	3	0.00647
68	A	31976	68.34523
68	S	3232	6.90805
68	T	2528	5.40333
68	V	2066	4.41585
68	G	2054	4.3902
68	D	1201	2.56701
68	R	796	1.70136
68	L	607	1.2974
68	E	512	1.09434
68	I	454	0.97038
68	P	434	0.92763
68	H	361	0.7716
68	K	148	0.31633
68	Q	147	0.3142
68	N	118	0.25221
68	F	65	0.13893
68	Y	55	0.11756
68	M	15	0.03206
68	W	12	0.02565
68	C	5	0.01069

69	D	38529	81.81818
69	E	2700	5.73358
69	N	1324	2.81158
69	A	1302	2.76486
69	G	903	1.91756
69	H	409	0.86853
69	S	406	0.86216
69	P	312	0.66255
69	R	270	0.57336
69	T	214	0.45444
69	V	204	0.4332
69	K	168	0.35676
69	Y	143	0.30367
69	Q	126	0.26757
69	L	39	0.08282
69	I	22	0.04672
69	W	11	0.02336
69	F	5	0.01062
69	M	3	0.00637
69	C	1	0.00212
70	S	42550	88.21395
70	F	1747	3.62185
70	A	1691	3.50575
70	P	745	1.54452
70	T	417	0.86452
70	Y	256	0.53073
70	D	229	0.47476
70	V	175	0.36281
70	L	105	0.21768
70	G	70	0.14512
70	R	47	0.09744
70	N	46	0.09537
70	W	39	0.08085
70	Q	36	0.07463
70	H	31	0.06427
70	I	31	0.06427
70	E	6	0.01244
70	K	5	0.01037
70	M	5	0.01037
70	C	4	0.00829
71	V	45883	93.48615
71	A	1833	3.73472
71	L	453	0.92298
71	M	424	0.8639
71	I	193	0.39324
71	E	111	0.22616

71	G	51	0.10391
71	T	41	0.08354
71	S	26	0.05297
71	F	22	0.04482
71	N	11	0.02241
71	D	9	0.01834
71	P	5	0.01019
71	Q	5	0.01019
71	R	5	0.01019
71	K	4	0.00815
71	W	3	0.00611
71	H	1	0.00204
72	K	42831	86.88006
72	E	1576	3.19682
72	R	1441	2.92298
72	Q	1399	2.83779
72	N	527	1.06899
72	L	353	0.71604
72	T	313	0.6349
72	M	252	0.51117
72	A	239	0.4848
72	S	159	0.32252
72	V	72	0.14605
72	W	37	0.07505
72	G	35	0.071
72	D	30	0.06085
72	I	15	0.03043
72	H	12	0.02434
72	F	4	0.00811
72	P	2	0.00406
72	Y	2	0.00406
73	NA	0	0
74	G	47373	95.14943
74	D	1561	3.13529
74	A	396	0.79537
74	S	119	0.23901
74	E	117	0.235
74	R	64	0.12855
74	V	60	0.12051
74	L	30	0.06026
74	T	21	0.04218
74	C	20	0.04017
74	N	12	0.0241
74	Y	9	0.01808
74	H	3	0.00603
74	P	2	0.00402

74	K	1	0.00201
75	R	49764	99.49815
75	Q	134	0.26792
75	L	33	0.06598
75	H	30	0.05998
75	G	18	0.03599
75	C	11	0.02199
75	P	10	0.01999
75	W	9	0.01799
75	K	3	0.006
75	S	3	0.006
76	F	48787	97.35787
76	L	346	0.69047
76	S	301	0.60067
76	V	270	0.5388
76	A	141	0.28138
76	Y	123	0.24546
76	I	79	0.15765
76	C	39	0.07783
76	M	7	0.01397
76	H	5	0.00998
76	W	5	0.00998
76	T	4	0.00798
76	G	3	0.00599
76	K	1	0.002
77	T	45154	89.93746
77	A	1956	3.89595
77	S	1273	2.53555
77	I	974	1.94001
77	V	202	0.40234
77	N	196	0.39039
77	F	124	0.24698
77	R	77	0.15337
77	L	50	0.09959
77	D	43	0.08565
77	P	34	0.06772
77	G	31	0.06175
77	K	26	0.05179
77	M	21	0.04183
77	E	18	0.03585
77	H	17	0.03386
77	Y	8	0.01593
77	C	1	0.00199
77	W	1	0.00199
78	I	44231	87.92565
78	V	3469	6.89593

78	L	1044	2.07534
78	T	415	0.82497
78	A	387	0.76931
78	M	322	0.6401
78	F	238	0.47311
78	N	61	0.12126
78	Y	42	0.08349
78	S	36	0.07156
78	C	17	0.03379
78	G	16	0.03181
78	R	9	0.01789
78	P	5	0.00994
78	W	5	0.00994
78	H	4	0.00795
78	D	1	0.00199
78	E	1	0.00199
78	K	1	0.00199
78	Q	1	0.00199
79	S	46945	93.12267
79	T	1145	2.27128
79	A	900	1.78529
79	F	593	1.17631
79	P	200	0.39673
79	Y	149	0.29556
79	V	142	0.28168
79	D	55	0.1091
79	L	54	0.10712
79	H	52	0.10315
79	I	51	0.10117
79	W	44	0.08728
79	R	38	0.07538
79	N	21	0.04166
79	G	20	0.03967
79	C	3	0.00595
80	K	15307	30.33492
80	Q	13899	27.54459
80	R	12393	24.56005
80	H	3157	6.25644
80	L	1775	3.51764
80	E	1269	2.51486
80	T	504	0.99881
80	I	370	0.73325
80	V	345	0.68371
80	A	220	0.43599
80	Y	220	0.43599
80	G	194	0.38446

80	S	191	0.37852
80	N	185	0.36663
80	P	180	0.35672
80	F	78	0.15458
80	M	75	0.14863
80	W	65	0.12881
80	D	30	0.05945
80	C	3	0.00595
81	D	45794	90.68119
81	G	1322	2.61782
81	V	1022	2.02376
81	N	839	1.66139
81	E	721	1.42772
81	A	363	0.71881
81	H	116	0.2297
81	S	105	0.20792
81	Q	40	0.07921
81	T	39	0.07723
81	Y	39	0.07723
81	R	37	0.07327
81	I	20	0.0396
81	P	17	0.03366
81	L	13	0.02574
81	F	8	0.01584
81	K	5	0.0099
82	N	33625	66.53014
82	S	3995	7.90447
82	D	2900	5.73792
82	K	2806	5.55193
82	T	1564	3.09452
82	I	905	1.79063
82	R	892	1.7649
82	Y	847	1.67587
82	G	603	1.19309
82	V	593	1.1733
82	A	463	0.91609
82	H	460	0.91015
82	L	223	0.44123
82	E	170	0.33636
82	Q	149	0.29481
82	M	122	0.24139
82	P	108	0.21369
82	F	95	0.18797
82	C	18	0.03561
82	W	3	0.00594
83	A	38757	76.63424

83	T	3487	6.89485
83	S	1849	3.65603
83	D	1670	3.30209
83	G	1316	2.60213
83	V	1314	2.59817
83	P	539	1.06577
83	N	414	0.8186
83	R	360	0.71183
83	E	220	0.43501
83	I	189	0.37371
83	F	150	0.2966
83	L	111	0.21948
83	Y	87	0.17203
83	H	64	0.12655
83	Q	23	0.04548
83	M	14	0.02768
83	W	7	0.01384
83	K	2	0.00395
83	C	1	0.00198
84	K	36552	72.24715
84	N	3656	7.2263
84	E	3050	6.0285
84	R	2180	4.3089
84	G	983	1.94296
84	Q	822	1.62473
84	T	813	1.60694
84	A	553	1.09304
84	D	457	0.90329
84	S	377	0.74516
84	M	312	0.61669
84	L	240	0.47437
84	V	220	0.43484
84	W	169	0.33404
84	H	96	0.18975
84	I	50	0.09883
84	Y	29	0.05732
84	P	20	0.03953
84	F	14	0.02767
85	N	36946	73.01581
85	K	3559	7.0336
85	S	2381	4.70553
85	D	1949	3.85178
85	T	1357	2.68182
85	R	1333	2.63439
85	H	681	1.34585
85	G	564	1.11462

85	A	442	0.87352
85	Y	384	0.75889
85	M	230	0.45455
85	I	173	0.3419
85	E	170	0.33597
85	Q	157	0.31028
85	P	89	0.17589
85	L	87	0.17194
85	V	64	0.12648
85	F	28	0.05534
85	C	5	0.00988
85	W	1	0.00198
86	T	42008	83.00828
86	I	2748	5.43008
86	A	1447	2.85929
86	M	1408	2.78222
86	S	1123	2.21906
86	V	600	1.18561
86	N	281	0.55526
86	K	212	0.41891
86	R	201	0.39718
86	L	200	0.3952
86	P	122	0.24107
86	E	117	0.23119
86	Q	40	0.07904
86	F	36	0.07114
86	Y	22	0.04347
86	G	21	0.0415
86	D	13	0.02569
86	H	3	0.00593
86	W	3	0.00593
86	C	2	0.00395
87	L	29556	58.39837
87	V	15167	29.96779
87	M	2232	4.41011
87	I	1185	2.34139
87	A	675	1.3337
87	T	447	0.88321
87	H	401	0.79232
87	F	221	0.43666
87	W	171	0.33787
87	P	117	0.23118
87	Q	106	0.20944
87	Y	94	0.18573
87	S	90	0.17783
87	G	60	0.11855

87	E	30	0.05928
87	R	29	0.0573
87	C	22	0.04347
87	K	6	0.01186
87	D	1	0.00198
87	N	1	0.00198
88	Y	39230	77.50667
88	F	2792	5.51615
88	H	2084	4.11736
88	D	1551	3.06431
88	S	1416	2.79759
88	T	1008	1.9915
88	N	670	1.32372
88	W	396	0.78238
88	L	338	0.66779
88	V	297	0.58678
88	I	293	0.57888
88	A	235	0.46429
88	C	108	0.21338
88	R	69	0.13632
88	E	52	0.10274
88	Q	26	0.05137
88	M	19	0.03754
88	P	17	0.03359
88	G	9	0.01778
88	K	5	0.00988
89	L	50232	99.23154
89	P	186	0.36744
89	Q	80	0.15804
89	M	46	0.09087
89	I	20	0.03951
89	R	16	0.03161
89	H	12	0.02371
89	S	11	0.02173
89	V	9	0.01778
89	F	8	0.0158
89	T	1	0.00198
90	Q	43608	86.13926
90	E	3574	7.05975
90	R	1058	2.08988
90	H	973	1.92198
90	L	425	0.83951
90	D	335	0.66173
90	K	143	0.28247
90	T	122	0.24099
90	A	92	0.18173

90	I	68	0.13432
90	V	53	0.10469
90	G	38	0.07506
90	M	34	0.06716
90	Y	28	0.05531
90	S	27	0.05333
90	N	21	0.04148
90	F	16	0.0316
90	P	9	0.01778
90	C	1	0.00198
91	M	47861	94.53838
91	L	1536	3.03401
91	I	739	1.45972
91	V	219	0.43258
91	T	156	0.30814
91	K	46	0.09086
91	A	40	0.07901
91	R	14	0.02765
91	S	9	0.01778
91	G	5	0.00988
91	P	1	0.00198
92	N	37718	74.50322
92	D	7011	13.84862
92	S	2556	5.04879
92	T	1549	3.05969
92	H	395	0.78023
92	I	233	0.46024
92	K	222	0.43851
92	E	198	0.3911
92	Y	173	0.34172
92	R	146	0.28839
92	A	123	0.24296
92	G	106	0.20938
92	V	66	0.13037
92	M	44	0.08691
92	L	29	0.05728
92	Q	24	0.04741
92	F	20	0.03951
92	P	6	0.01185
92	W	4	0.0079
92	C	3	0.00593
93	S	31458	62.13312
93	N	11172	22.06597
93	D	3017	5.95892
93	G	1250	2.46889
93	R	1188	2.34643

93	T	869	1.71637
93	A	673	1.32925
93	K	226	0.44638
93	M	172	0.33972
93	H	101	0.19949
93	I	99	0.19554
93	E	88	0.17381
93	Y	76	0.15011
93	V	53	0.10468
93	C	44	0.0869
93	Q	41	0.08098
93	F	35	0.06913
93	L	34	0.06715
93	P	33	0.06518
93	W	1	0.00198
94	L	49293	97.35735
94	V	597	1.17912
94	P	225	0.44439
94	M	184	0.36341
94	A	129	0.25478
94	Q	74	0.14616
94	I	71	0.14023
94	T	27	0.05333
94	R	17	0.03358
94	S	7	0.01383
94	F	3	0.00593
94	H	2	0.00395
94	D	1	0.00198
94	K	1	0.00198
95	K	37268	73.60708
95	Q	3070	6.06348
95	E	3065	6.0536
95	R	2248	4.43997
95	T	1738	3.43268
95	N	1400	2.7651
95	S	324	0.63992
95	I	316	0.62412
95	M	299	0.59055
95	A	246	0.48587
95	L	193	0.38119
95	V	157	0.31009
95	G	147	0.29034
95	D	86	0.16986
95	H	66	0.13035
95	Y	7	0.01383
95	W	1	0.00198

96	P	42389	83.72144
96	T	2288	4.51897
96	S	1838	3.63019
96	A	1098	2.16863
96	L	949	1.87435
96	V	815	1.60969
96	I	450	0.88878
96	R	186	0.36736
96	F	169	0.33379
96	Q	139	0.27454
96	D	115	0.22713
96	H	72	0.14221
96	Y	55	0.10863
96	E	40	0.079
96	G	10	0.01975
96	N	9	0.01778
96	M	6	0.01185
96	C	2	0.00395
96	K	1	0.00198
97	E	45003	88.88428
97	D	3844	7.59219
97	G	578	1.14159
97	A	301	0.5945
97	N	238	0.47007
97	Q	196	0.38711
97	V	125	0.24688
97	K	116	0.22911
97	S	94	0.18566
97	L	44	0.0869
97	T	39	0.07703
97	M	30	0.05925
97	R	10	0.01975
97	Y	6	0.01185
97	H	3	0.00593
97	W	3	0.00593
97	P	1	0.00198
98	D	50170	99.08949
98	G	165	0.32589
98	E	114	0.22516
98	N	74	0.14616
98	V	61	0.12048
98	A	23	0.04543
98	Y	15	0.02963
98	H	9	0.01778
99	T	45333	89.5449
99	S	4358	8.60823

99	A	696	1.37479
99	I	67	0.13234
99	G	65	0.12839
99	N	35	0.06913
99	M	20	0.03951
99	R	16	0.0316
99	P	15	0.02963
99	E	10	0.01975
99	C	4	0.0079
99	K	3	0.00593
99	D	2	0.00395
99	F	1	0.00198
99	V	1	0.00198
100	A	43183	85.30818
100	G	6360	12.5642
100	S	576	1.13789
100	D	214	0.42276
100	T	104	0.20545
100	V	66	0.13038
100	N	39	0.07704
100	R	15	0.02963
100	E	13	0.02568
100	H	12	0.02371
100	P	12	0.02371
100	Y	11	0.02173
100	W	9	0.01778
100	F	3	0.00593
100	C	2	0.00395
100	I	1	0.00198
101	M	32770	64.73726
101	V	7662	15.13631
101	T	3816	7.53852
101	I	2765	5.46227
101	L	1279	2.52667
101	R	835	1.64955
101	K	758	1.49743
101	A	331	0.65389
101	E	174	0.34374
101	N	67	0.13236
101	S	55	0.10865
101	D	34	0.06717
101	Q	30	0.05927
101	W	21	0.04149
101	G	7	0.01383
101	P	7	0.01383
101	F	4	0.0079

101	H	4	0.0079
101	C	1	0.00198
102	Y	50127	99.03195
102	H	166	0.32795
102	C	135	0.26671
102	F	124	0.24498
102	N	46	0.09088
102	S	9	0.01778
102	D	6	0.01185
102	L	2	0.00395
102	R	1	0.00198
102	V	1	0.00198
103	Y	44223	87.74753
103	F	1955	3.87912
103	S	1287	2.55367
103	H	693	1.37505
103	I	532	1.0556
103	V	470	0.93258
103	T	431	0.85519
103	L	168	0.33335
103	N	143	0.28374
103	Q	141	0.27977
103	C	122	0.24207
103	R	109	0.21628
103	W	68	0.13493
103	A	10	0.01984
103	D	10	0.01984
103	M	10	0.01984
103	G	9	0.01786
103	P	7	0.01389
103	E	5	0.00992
103	K	5	0.00992
104	C	50340	99.91465
104	R	20	0.0397
104	Y	12	0.02382
104	W	9	0.01786
104	S	2	0.00397
105	A	5162	94.85483
105	V	160	2.9401
105	G	45	0.8269
105	S	26	0.47777
105	T	24	0.44101
105	L	14	0.25726
105	E	8	0.147
105	K	2	0.03675
105	P	1	0.01838

106	A	4629	91.05035
106	T	167	3.28482
106	V	71	1.39654
106	P	63	1.23918
106	S	60	1.18017
106	G	35	0.68843
106	E	16	0.31471
106	R	12	0.23603
106	L	10	0.1967
106	K	9	0.17703
106	I	7	0.13769
106	Q	4	0.07868
106	D	1	0.01967

Table S7: 3-VH

The positions of amino acids	Amino acids	Counts	Percentage(%)
1	H	28806	64.60483
1	D	9868	22.13152
1	Q	3719	8.34081
1	E	925	2.07455
1	S	606	1.35911
1	M	252	0.56517
1	L	124	0.2781
1	Y	65	0.14578
1	A	59	0.13232
1	P	37	0.08298
1	N	28	0.0628
1	R	28	0.0628
1	G	25	0.05607
1	C	17	0.03813
1	W	15	0.03364
1	V	6	0.01346
1	K	5	0.01121
1	I	3	0.00673
2	V	43738	97.67743
2	C	594	1.32654
2	M	105	0.23449
2	G	83	0.18536
2	L	79	0.17643
2	W	73	0.16303
2	A	36	0.0804
2	H	19	0.04243
2	S	14	0.03127
2	E	11	0.02457
2	Q	9	0.0201
2	D	8	0.01787
2	R	8	0.01787
2	F	1	0.00223
3	Q	45865	98.09017
3	A	493	1.05437
3	S	107	0.22884
3	C	63	0.13474
3	P	48	0.10266
3	V	35	0.07485
3	R	34	0.07271
3	K	32	0.06844
3	L	31	0.0663
3	E	28	0.05988
3	H	13	0.0278

3	M	4	0.00855
3	W	4	0.00855
3	G	1	0.00214
4	L	46239	98.39341
4	A	420	0.89373
4	W	107	0.22769
4	P	50	0.1064
4	S	48	0.10214
4	V	47	0.10001
4	Q	39	0.08299
4	M	28	0.05958
4	R	9	0.01915
4	E	3	0.00638
4	C	2	0.00426
4	G	2	0.00426
5	V	46646	98.79069
5	G	328	0.69467
5	W	129	0.27321
5	L	33	0.06989
5	E	25	0.05295
5	A	23	0.04871
5	M	23	0.04871
5	S	5	0.01059
5	F	3	0.00635
5	D	1	0.00212
5	T	1	0.00212
6	E	46806	98.91378
6	G	280	0.59172
6	S	64	0.13525
6	M	46	0.09721
6	W	31	0.06551
6	A	30	0.0634
6	V	25	0.05283
6	K	19	0.04015
6	D	7	0.01479
6	R	5	0.01057
6	Q	3	0.00634
6	L	2	0.00423
6	H	1	0.00211
6	Y	1	0.00211
7	S	46929	99.00633
7	V	241	0.50844
7	L	60	0.12658
7	A	58	0.12236
7	F	29	0.06118
7	Y	29	0.06118

7	P	23	0.04852
7	T	10	0.0211
7	E	7	0.01477
7	G	7	0.01477
7	C	2	0.00422
7	H	2	0.00422
7	D	1	0.00211
7	Q	1	0.00211
7	W	1	0.00211
8	G	47041	99.17984
8	W	252	0.53131
8	L	31	0.06536
8	H	30	0.06325
8	R	22	0.04638
8	E	17	0.03584
8	D	15	0.03163
8	S	9	0.01898
8	V	4	0.00843
8	P	3	0.00633
8	Q	3	0.00633
8	A	2	0.00422
8	C	1	0.00211
9	G	47176	99.3409
9	E	106	0.22321
9	R	77	0.16214
9	V	68	0.14319
9	M	33	0.06949
9	A	10	0.02106
9	C	3	0.00632
9	L	3	0.00632
9	P	3	0.00632
9	W	3	0.00632
9	D	2	0.00421
9	Q	2	0.00421
9	K	1	0.00211
9	S	1	0.00211
9	T	1	0.00211
10	NA	0	0
11	G	39985	84.14706
11	D	4926	10.3666
11	A	1004	2.11288
11	S	413	0.86914
11	E	406	0.85441
11	R	189	0.39774
11	N	170	0.35776
11	T	168	0.35355

11	V	103	0.21676
11	Q	53	0.11154
11	H	37	0.07787
11	K	28	0.05893
11	C	20	0.04209
11	L	5	0.01052
11	W	4	0.00842
11	F	3	0.00631
11	M	2	0.00421
11	Y	2	0.00421
12	L	38255	80.47924
12	S	7644	16.08112
12	V	454	0.95511
12	T	220	0.46283
12	F	205	0.43127
12	A	156	0.32819
12	W	138	0.29032
12	M	133	0.2798
12	Q	95	0.19986
12	P	77	0.16199
12	E	37	0.07784
12	H	36	0.07574
12	R	27	0.0568
12	D	16	0.03366
12	Y	16	0.03366
12	K	15	0.03156
12	I	5	0.01052
12	G	4	0.00842
12	N	1	0.0021
13	V	46056	96.86008
13	A	879	1.84862
13	M	152	0.31967
13	L	151	0.31757
13	G	122	0.25658
13	E	80	0.16825
13	I	41	0.08623
13	C	26	0.05468
13	T	13	0.02734
13	R	7	0.01472
13	W	6	0.01262
13	Q	5	0.01052
13	K	4	0.00841
13	S	4	0.00841
13	P	2	0.00421
13	N	1	0.0021
14	Q	43326	91.10139

14	R	1681	3.53463
14	E	718	1.50974
14	H	595	1.2511
14	K	406	0.85369
14	L	300	0.63081
14	P	281	0.59086
14	A	84	0.17663
14	S	46	0.09672
14	G	29	0.06098
14	T	29	0.06098
14	N	22	0.04626
14	C	10	0.02103
14	D	8	0.01682
14	V	7	0.01472
14	Y	7	0.01472
14	M	5	0.01051
14	W	4	0.00841
15	P	40125	84.35825
15	A	5764	12.11815
15	T	628	1.3203
15	S	405	0.85147
15	V	219	0.46042
15	L	122	0.25649
15	D	62	0.13035
15	I	56	0.11773
15	F	49	0.10302
15	G	34	0.07148
15	N	34	0.07148
15	R	24	0.05046
15	H	21	0.04415
15	E	11	0.02313
15	Y	5	0.01051
15	Q	4	0.00841
15	C	1	0.0021
15	M	1	0.0021
16	G	46976	98.71189
16	R	270	0.56736
16	E	144	0.30259
16	W	100	0.21013
16	A	33	0.06934
16	D	26	0.05463
16	V	25	0.05253
16	L	8	0.01681
16	S	4	0.00841
16	C	1	0.0021
16	M	1	0.0021

16	T	1	0.0021
17	G	44334	93.13474
17	E	1603	3.36751
17	R	471	0.98945
17	D	350	0.73526
17	A	194	0.40755
17	V	160	0.33612
17	K	133	0.2794
17	N	85	0.17856
17	Q	77	0.16176
17	W	67	0.14075
17	S	45	0.09453
17	T	43	0.09033
17	M	19	0.03991
17	H	13	0.02731
17	L	4	0.0084
17	Y	3	0.0063
17	C	1	0.0021
18	S	46817	98.22092
18	T	284	0.59583
18	P	219	0.45946
18	F	132	0.27693
18	A	101	0.2119
18	Y	38	0.07972
18	D	13	0.02727
18	R	12	0.02518
18	V	12	0.02518
18	C	9	0.01888
18	M	7	0.01469
18	G	5	0.01049
18	L	4	0.00839
18	E	3	0.00629
18	I	3	0.00629
18	H	2	0.0042
18	N	2	0.0042
18	Q	1	0.0021
18	W	1	0.0021
19	L	46206	96.83544
19	V	468	0.9808
19	M	389	0.81524
19	P	211	0.4422
19	R	183	0.38352
19	Q	113	0.23682
19	A	58	0.12155
19	S	23	0.0482
19	T	20	0.04191

19	H	17	0.03563
19	I	13	0.02724
19	W	5	0.01048
19	E	4	0.00838
19	F	2	0.00419
19	G	2	0.00419
19	K	2	0.00419
20	R	39527	82.8016
20	T	4512	9.45179
20	K	1185	2.48235
20	S	874	1.83086
20	V	692	1.44961
20	G	317	0.66406
20	I	291	0.60959
20	N	97	0.2032
20	Q	67	0.14035
20	M	60	0.12569
20	L	45	0.09427
20	A	41	0.08589
20	E	16	0.03352
20	D	6	0.01257
20	C	5	0.01047
20	H	1	0.00209
20	P	1	0.00209
21	L	45723	95.61281
21	V	1312	2.74356
21	I	295	0.61688
21	P	231	0.48305
21	F	111	0.23212
21	H	78	0.16311
21	G	36	0.07528
21	R	13	0.02718
21	A	10	0.02091
21	M	6	0.01255
21	T	2	0.00418
21	E	1	0.00209
21	Q	1	0.00209
21	S	1	0.00209
21	W	1	0.00209
22	S	45967	96.02064
22	A	712	1.4873
22	T	637	1.33063
22	P	229	0.47836
22	F	72	0.1504
22	D	47	0.09818
22	R	41	0.08565

22	V	32	0.06684
22	L	25	0.05222
22	I	24	0.05013
22	G	23	0.04804
22	Y	22	0.04596
22	N	11	0.02298
22	Q	8	0.01671
22	K	7	0.01462
22	C	6	0.01253
22	H	5	0.01044
22	E	3	0.00627
22	W	1	0.00209
23	C	47469	99.07125
23	R	205	0.42785
23	S	86	0.17949
23	Y	81	0.16905
23	G	26	0.05426
23	W	20	0.04174
23	F	12	0.02504
23	L	11	0.02296
23	V	2	0.00417
23	A	1	0.00209
23	E	1	0.00209
24	A	32589	67.96313
24	V	6577	13.71609
24	T	3995	8.33142
24	E	1378	2.87377
24	S	1034	2.15637
24	G	635	1.32427
24	L	433	0.90301
24	I	324	0.67569
24	R	283	0.59019
24	K	267	0.55682
24	Q	199	0.41501
24	D	151	0.3149
24	P	24	0.05005
24	H	21	0.04379
24	N	20	0.04171
24	F	10	0.02085
24	M	6	0.01251
24	Y	3	0.00626
24	C	2	0.00417
25	A	39096	81.43643
25	T	2765	5.75946
25	G	2452	5.10748
25	V	2188	4.55757

25	S	327	0.68114
25	Y	253	0.527
25	P	246	0.51241
25	F	185	0.38535
25	I	134	0.27912
25	L	124	0.25829
25	D	68	0.14164
25	C	58	0.12081
25	H	36	0.07499
25	M	22	0.04583
25	E	19	0.03958
25	R	14	0.02916
25	W	10	0.02083
25	N	8	0.01666
25	Q	2	0.00417
25	K	1	0.00208
26	S	45840	95.41452
26	T	755	1.57151
26	F	354	0.73684
26	P	315	0.65566
26	A	307	0.63901
26	D	155	0.32263
26	Y	83	0.17276
26	L	63	0.13113
26	V	32	0.06661
26	I	28	0.05828
26	R	26	0.05412
26	G	21	0.04371
26	H	21	0.04371
26	N	16	0.0333
26	C	7	0.01457
26	E	6	0.01249
26	K	5	0.01041
26	Q	4	0.00833
26	W	4	0.00833
26	M	1	0.00208
27	G	45406	94.4738
27	E	798	1.66036
27	R	608	1.26503
27	A	298	0.62003
27	T	191	0.3974
27	Q	141	0.29337
27	D	131	0.27256
27	V	119	0.2476
27	N	105	0.21847
27	S	71	0.14773

27	K	55	0.11444
27	L	51	0.10611
27	P	30	0.06242
27	I	28	0.05826
27	M	10	0.02081
27	W	8	0.01665
27	F	5	0.0104
27	Y	4	0.00832
27	C	2	0.00416
27	H	1	0.00208
28	F	41781	86.88808
28	L	1728	3.59356
28	Y	1383	2.8761
28	I	830	1.72607
28	S	781	1.62417
28	V	372	0.77361
28	W	245	0.5095
28	D	243	0.50534
28	A	169	0.35145
28	N	110	0.22876
28	G	104	0.21628
28	H	79	0.16429
28	T	68	0.14141
28	R	58	0.12062
28	P	56	0.11646
28	C	30	0.06239
28	E	22	0.04575
28	K	9	0.01872
28	M	9	0.01872
28	Q	9	0.01872
29	T	34512	71.72368
29	S	2787	5.79201
29	A	2772	5.76084
29	I	1871	3.88836
29	P	1359	2.82431
29	V	927	1.92651
29	R	764	1.58776
29	Q	531	1.10354
29	K	517	1.07444
29	D	503	1.04535
29	N	417	0.86662
29	G	267	0.55489
29	E	235	0.48838
29	M	230	0.47799
29	L	183	0.38032
29	F	128	0.26601

29	Y	63	0.13093
29	W	24	0.04988
29	H	23	0.0478
29	C	5	0.01039
30	F	40400	83.91492
30	L	1631	3.38775
30	S	1577	3.27559
30	Y	1187	2.46552
30	V	876	1.81954
30	I	555	1.15279
30	A	435	0.90354
30	M	225	0.46735
30	T	222	0.46112
30	G	209	0.43411
30	D	174	0.36142
30	Q	150	0.31157
30	P	103	0.21394
30	R	102	0.21186
30	W	91	0.18902
30	E	65	0.13501
30	N	58	0.12047
30	H	38	0.07893
30	K	24	0.04985
30	C	22	0.0457
31	S	35032	72.74237
31	R	2555	5.30534
31	G	2416	5.01672
31	N	2258	4.68864
31	T	1924	3.9951
31	D	1342	2.7866
31	A	919	1.90826
31	E	409	0.84927
31	K	351	0.72884
31	V	217	0.45059
31	I	142	0.29486
31	C	123	0.2554
31	H	85	0.1765
31	P	84	0.17442
31	Y	84	0.17442
31	M	56	0.11628
31	F	51	0.1059
31	Q	44	0.09136
31	W	35	0.07268
31	L	32	0.06645
32	S	14025	29.1114
32	T	7726	16.0367

32	N	6993	14.51523
32	D	5458	11.32906
32	G	3401	7.05939
32	R	3384	7.0241
32	A	1536	3.18824
32	I	872	1.80999
32	K	677	1.40523
32	L	652	1.35334
32	V	617	1.28069
32	H	572	1.18729
32	Y	513	1.06482
32	E	486	1.00878
32	M	461	0.95689
32	P	297	0.61648
32	F	221	0.45873
32	Q	185	0.384
32	W	66	0.13699
32	C	35	0.07265
33	Y	30670	63.61355
33	H	2645	5.48607
33	A	2317	4.80576
33	S	2270	4.70827
33	N	2257	4.68131
33	F	1592	3.30201
33	V	1512	3.13608
33	T	1194	2.47651
33	R	596	1.23618
33	L	591	1.22581
33	D	556	1.15322
33	W	382	0.79232
33	K	303	0.62846
33	I	299	0.62016
33	C	294	0.60979
33	G	236	0.48949
33	Q	177	0.36712
33	E	174	0.3609
33	M	90	0.18667
33	P	58	0.1203
34	W	10691	22.15109
34	Y	9852	20.41273
34	A	7215	14.94903
34	D	4340	8.99221
34	G	2757	5.71233
34	T	2347	4.86284
34	S	2242	4.64528
34	C	1770	3.66733

34	P	1449	3.00224
34	F	1361	2.81991
34	V	1284	2.66037
34	H	819	1.69692
34	R	693	1.43585
34	L	403	0.83499
34	Q	329	0.68167
34	E	264	0.54699
34	N	201	0.41646
34	I	196	0.4061
34	M	43	0.08909
34	K	8	0.01658
35	NA	0	0
36	NA	0	0
37	NA	0	0
38	NA	0	0
39	M	42080	87.05546
39	I	2738	5.6644
39	L	1446	2.9915
39	V	1154	2.38741
39	T	348	0.71995
39	F	208	0.43031
39	W	73	0.15102
39	A	70	0.14482
39	K	70	0.14482
39	Q	37	0.07655
39	R	34	0.07034
39	S	24	0.04965
39	G	21	0.04344
39	D	11	0.02276
39	P	9	0.01862
39	C	5	0.01034
39	E	3	0.00621
39	H	2	0.00414
39	N	2	0.00414
39	Y	2	0.00414
40	S	17321	35.73035
40	Y	9923	20.4695
40	T	6600	13.6147
40	N	4310	8.89081
40	G	2544	5.24785
40	A	2137	4.40828
40	H	1533	3.16232
40	R	1104	2.27737
40	I	866	1.78641
40	F	847	1.74722

40	M	356	0.73437
40	D	205	0.42288
40	C	160	0.33005
40	L	160	0.33005
40	W	153	0.31561
40	V	144	0.29705
40	K	59	0.12171
40	Q	28	0.05776
40	E	17	0.03507
40	P	10	0.02063
41	W	48416	99.28636
41	R	215	0.4409
41	Y	43	0.08818
41	C	39	0.07998
41	L	18	0.03691
41	G	13	0.02666
41	F	6	0.0123
41	V	6	0.0123
41	S	5	0.01025
41	H	2	0.0041
41	N	1	0.00205
42	V	43209	87.95904
42	I	2257	4.5945
42	F	1893	3.85351
42	A	734	1.49418
42	L	604	1.22954
42	Y	197	0.40103
42	D	72	0.14657
42	G	38	0.07736
42	S	32	0.06514
42	T	28	0.057
42	H	20	0.04071
42	P	9	0.01832
42	M	8	0.01629
42	E	7	0.01425
42	K	6	0.01221
42	Q	3	0.00611
42	R	3	0.00611
42	W	3	0.00611
42	C	1	0.00204
43	R	49023	99.23684
43	H	144	0.2915
43	C	114	0.23077
43	S	34	0.06883
43	P	22	0.04453
43	L	21	0.04251

43	G	12	0.02429
43	W	10	0.02024
43	Q	7	0.01417
43	T	5	0.01012
43	A	4	0.0081
43	F	2	0.00405
43	K	1	0.00202
43	V	1	0.00202
44	Q	47725	96.33435
44	R	822	1.65923
44	L	397	0.80136
44	H	337	0.68024
44	E	93	0.18772
44	K	54	0.109
44	P	32	0.06459
44	M	29	0.05854
44	W	17	0.03432
44	S	11	0.0222
44	D	6	0.01211
44	V	6	0.01211
44	A	5	0.01009
44	G	3	0.00606
44	T	2	0.00404
44	Y	2	0.00404
45	A	42117	84.91331
45	T	3094	6.2379
45	V	1628	3.28226
45	G	741	1.49395
45	P	596	1.20161
45	S	556	1.12097
45	R	311	0.62702
45	L	131	0.26411
45	N	111	0.22379
45	I	84	0.16935
45	F	76	0.15323
45	D	67	0.13508
45	H	26	0.05242
45	C	20	0.04032
45	K	15	0.03024
45	Q	12	0.02419
45	Y	6	0.0121
45	E	5	0.01008
45	M	3	0.00605
45	W	1	0.00202
46	P	47862	96.30762
46	A	445	0.89543

46	L	394	0.7928
46	S	353	0.7103
46	Q	292	0.58756
46	R	150	0.30183
46	T	59	0.11872
46	E	56	0.11268
46	H	34	0.06841
46	D	18	0.03622
46	K	10	0.02012
46	N	6	0.01207
46	W	6	0.01207
46	M	4	0.00805
46	C	3	0.00604
46	I	2	0.00402
46	V	2	0.00402
46	Y	1	0.00201
47	G	46729	93.94274
47	A	1939	3.89811
47	E	496	0.99715
47	R	215	0.43223
47	V	143	0.28748
47	D	85	0.17088
47	W	49	0.09851
47	N	24	0.04825
47	T	20	0.04021
47	L	13	0.02613
47	S	11	0.02211
47	Q	8	0.01608
47	K	5	0.01005
47	C	2	0.00402
47	H	1	0.00201
47	M	1	0.00201
47	P	1	0.00201
48	K	47240	94.88421
48	Q	679	1.36381
48	R	568	1.14086
48	E	441	0.88577
48	N	270	0.54231
48	M	151	0.30329
48	T	143	0.28722
48	L	78	0.15667
48	S	75	0.15064
48	A	60	0.12051
48	G	59	0.1185
48	V	8	0.01607
48	H	5	0.01004

48	W	3	0.00603
48	D	2	0.00402
48	I	2	0.00402
48	P	2	0.00402
48	Y	1	0.00201
49	G	46393	93.08947
49	E	1783	3.57766
49	A	682	1.36846
49	D	297	0.59594
49	R	254	0.50966
49	H	94	0.18861
49	V	67	0.13444
49	K	64	0.12842
49	Q	50	0.10033
49	P	34	0.06822
49	S	30	0.0602
49	W	26	0.05217
49	L	24	0.04816
49	T	19	0.03812
49	C	12	0.02408
49	N	3	0.00602
49	F	2	0.00401
49	M	2	0.00401
49	I	1	0.00201
50	L	44899	89.95452
50	R	1733	3.47204
50	P	1369	2.74277
50	F	990	1.98345
50	V	464	0.92962
50	I	153	0.30653
50	H	87	0.1743
50	M	61	0.12221
50	Q	40	0.08014
50	Y	28	0.0561
50	C	26	0.05209
50	S	21	0.04207
50	A	12	0.02404
50	W	9	0.01803
50	G	6	0.01202
50	T	6	0.01202
50	D	5	0.01002
50	E	3	0.00601
50	K	1	0.002
51	E	48139	96.3358
51	D	763	1.52692
51	Q	404	0.80849

51	G	195	0.39023
51	K	139	0.27817
51	A	123	0.24615
51	V	107	0.21413
51	R	28	0.05603
51	L	21	0.04203
51	W	16	0.03202
51	T	14	0.02802
51	S	6	0.01201
51	N	4	0.008
51	Y	4	0.008
51	M	3	0.006
51	H	2	0.004
51	F	1	0.002
51	I	1	0.002
52	W	46883	93.68917
52	G	1153	2.30411
52	R	584	1.16704
52	Y	516	1.03115
52	C	328	0.65546
52	L	184	0.3677
52	S	89	0.17785
52	F	83	0.16586
52	A	72	0.14388
52	V	49	0.09792
52	E	34	0.06794
52	H	16	0.03197
52	M	12	0.02398
52	P	9	0.01799
52	D	7	0.01399
52	T	6	0.01199
52	Q	5	0.00999
52	I	4	0.00799
52	K	4	0.00799
52	N	3	0.006
53	V	46373	92.54425
53	I	1898	3.78774
53	L	1143	2.28103
53	A	222	0.44303
53	M	165	0.32928
53	T	96	0.19158
53	G	46	0.0918
53	D	45	0.0898
53	E	39	0.07783
53	F	32	0.06386
53	C	13	0.02594

53	S	12	0.02395
53	R	9	0.01796
53	P	8	0.01597
53	Y	3	0.00599
53	H	2	0.00399
53	N	2	0.00399
53	Q	1	0.002
54	S	40220	80.07964
54	A	7571	15.07417
54	C	787	1.56695
54	T	714	1.4216
54	G	498	0.99154
54	P	139	0.27675
54	V	79	0.15729
54	L	74	0.14734
54	I	48	0.09557
54	F	38	0.07566
54	Y	18	0.03584
54	Q	13	0.02588
54	W	9	0.01792
54	M	6	0.01195
54	R	6	0.01195
54	D	2	0.00398
54	E	2	0.00398
54	N	1	0.00199
55	S	15675	31.13764
55	T	8675	17.23247
55	G	8147	16.18363
55	A	4627	9.19132
55	D	2460	4.88667
55	R	2086	4.14374
55	V	1746	3.46835
55	L	1715	3.40677
55	H	864	1.71629
55	N	812	1.613
55	Q	717	1.42429
55	I	687	1.36469
55	Y	652	1.29517
55	F	476	0.94555
55	K	286	0.56813
55	C	258	0.5125
55	M	193	0.38339
55	E	187	0.37147
55	W	61	0.12117
55	P	17	0.03377
56	I	41460	82.25538

56	V	4833	9.58852
56	L	1134	2.24982
56	M	760	1.50782
56	T	608	1.20625
56	F	524	1.0396
56	S	271	0.53766
56	A	222	0.44044
56	R	153	0.30355
56	C	134	0.26585
56	N	88	0.17459
56	G	63	0.12499
56	D	53	0.10515
56	H	27	0.05357
56	Y	25	0.0496
56	Q	14	0.02778
56	K	13	0.02579
56	P	12	0.02381
56	W	8	0.01587
56	E	2	0.00397
57	N	14664	29.05949
57	Y	8570	16.98308
57	S	7760	15.37791
57	D	4166	8.25572
57	A	3746	7.42341
57	T	2643	5.2376
57	G	1149	2.27696
57	H	1134	2.24724
57	L	982	1.94602
57	R	978	1.93809
57	K	911	1.80532
57	V	816	1.61706
57	F	572	1.13353
57	I	497	0.9849
57	W	496	0.98292
57	E	413	0.81844
57	Q	338	0.66981
57	P	260	0.51524
57	M	206	0.40823
57	C	161	0.31905
58	S	23070	45.63618
58	T	5302	10.48821
58	G	4105	8.12035
58	N	2959	5.85338
58	R	2249	4.44888
58	P	2075	4.10468
58	A	2027	4.00973

58	D	1417	2.80305
58	Y	1371	2.71206
58	I	1122	2.2195
58	K	908	1.79617
58	E	893	1.7665
58	W	844	1.66957
58	V	482	0.95347
58	H	418	0.82687
58	L	383	0.75764
58	F	343	0.67851
58	M	279	0.55191
58	Q	227	0.44904
58	C	78	0.1543
59	D	17658	34.85934
59	G	15278	30.16089
59	S	6103	12.04817
59	N	3217	6.3508
59	A	2278	4.49709
59	R	1613	3.18429
59	T	1057	2.08666
59	V	676	1.33452
59	E	603	1.19041
59	Y	546	1.07788
59	L	420	0.82914
59	I	361	0.71266
59	H	258	0.50933
59	F	150	0.29612
59	P	145	0.28625
59	Q	133	0.26256
59	K	87	0.17175
59	C	28	0.05528
59	W	23	0.04541
59	M	21	0.04146
60	G	41371	81.53688
60	S	2938	5.79042
60	D	2123	4.18416
60	A	1312	2.58578
60	T	568	1.11945
60	N	566	1.11551
60	R	391	0.77061
60	F	269	0.53016
60	E	260	0.51243
60	V	226	0.44542
60	I	147	0.28972
60	H	124	0.24439
60	P	116	0.22862

60	L	100	0.19709
60	K	93	0.18329
60	Y	71	0.13993
60	Q	25	0.04927
60	C	21	0.04139
60	M	15	0.02956
60	W	3	0.00591
61	G	15288	30.08146
61	S	12320	24.24147
61	T	5106	10.04683
61	R	4407	8.67144
61	D	4389	8.63602
61	A	1880	3.69919
61	V	1296	2.55008
61	N	1043	2.05226
61	I	983	1.9342
61	E	963	1.89485
61	K	865	1.70202
61	W	635	1.24946
61	Y	429	0.84412
61	H	224	0.44075
61	L	221	0.43485
61	M	219	0.43092
61	Q	210	0.41321
61	F	159	0.31286
61	P	140	0.27547
61	C	45	0.08854
62	T	13689	26.90818
62	S	10927	21.47898
62	N	6503	12.78281
62	R	3285	6.45726
62	I	2328	4.5761
62	K	2292	4.50534
62	V	1916	3.76624
62	G	1722	3.3849
62	A	1657	3.25713
62	D	1621	3.18637
62	Y	1478	2.90527
62	P	631	1.24034
62	E	574	1.1283
62	L	539	1.0595
62	H	538	1.05754
62	Q	479	0.94156
62	M	362	0.71158
62	F	214	0.42066
62	W	91	0.17888

62	C	27	0.05307
63	T	35436	77.3847
63	A	2039	4.45274
63	S	1770	3.8653
63	I	1686	3.68187
63	P	1592	3.47659
63	R	726	1.58543
63	K	594	1.29717
63	V	587	1.28188
63	L	475	1.0373
63	Q	339	0.7403
63	G	141	0.30791
63	E	116	0.25332
63	N	95	0.20746
63	M	88	0.19217
63	D	70	0.15287
63	F	25	0.05459
63	Y	6	0.0131
63	H	4	0.00874
63	C	2	0.00437
63	W	1	0.00218
63	NA	0	0
64	NA	0	0
65	NA	0	0
66	Y	26142	51.20762
66	N	3728	7.3025
66	L	2455	4.80892
66	E	2332	4.56798
66	D	2251	4.40932
66	F	1934	3.78837
66	H	1894	3.71002
66	R	1877	3.67672
66	S	1866	3.65517
66	A	1460	2.85989
66	T	1285	2.51709
66	V	1105	2.1645
66	W	946	1.85305
66	G	659	1.29087
66	I	273	0.53476
66	Q	273	0.53476
66	K	207	0.40548
66	C	145	0.28403
66	M	123	0.24094
66	P	96	0.18805
67	Y	47375	92.38314
67	S	1025	1.99879

67	F	446	0.86972
67	C	440	0.85802
67	V	407	0.79367
67	L	321	0.62596
67	H	277	0.54016
67	A	199	0.38806
67	D	199	0.38806
67	N	190	0.37051
67	I	154	0.30031
67	T	103	0.20085
67	Q	45	0.08775
67	W	36	0.0702
67	E	25	0.04875
67	G	17	0.03315
67	R	10	0.0195
67	M	6	0.0117
67	P	4	0.0078
67	K	2	0.0039
68	A	36141	70.06242
68	S	3536	6.85484
68	T	2520	4.88524
68	V	1786	3.46231
68	P	1694	3.28396
68	L	1354	2.62484
68	G	1333	2.58413
68	R	927	1.79707
68	H	490	0.94991
68	E	434	0.84135
68	N	389	0.75411
68	D	332	0.64361
68	Q	232	0.44975
68	K	191	0.37027
68	I	151	0.29273
68	C	40	0.07754
68	M	24	0.04653
68	Y	8	0.01551
68	F	1	0.00194
68	W	1	0.00194
69	D	44433	85.74985
69	E	2260	4.3615
69	N	1159	2.23672
69	A	939	1.81215
69	G	672	1.29687
69	S	441	0.85107
69	P	400	0.77195
69	T	316	0.60984

69	Y	230	0.44387
69	H	200	0.38597
69	V	182	0.35124
69	K	155	0.29913
69	R	133	0.25667
69	W	104	0.20071
69	L	94	0.18141
69	Q	71	0.13702
69	I	18	0.03474
69	M	9	0.01737
69	F	1	0.00193
70	S	48273	92.10471
70	F	1062	2.02629
70	A	996	1.90036
70	P	803	1.53212
70	T	365	0.69642
70	L	139	0.26521
70	N	137	0.2614
70	R	113	0.2156
70	V	97	0.18508
70	D	66	0.12593
70	Y	64	0.12211
70	I	63	0.1202
70	G	57	0.10876
70	Q	55	0.10494
70	H	43	0.08204
70	M	32	0.06106
70	C	25	0.0477
70	K	10	0.01908
70	W	6	0.01145
70	E	5	0.00954
71	V	45706	86.59884
71	I	2085	3.95043
71	A	1949	3.69276
71	M	1284	2.43279
71	L	1280	2.42521
71	G	143	0.27094
71	E	102	0.19326
71	T	96	0.18189
71	F	49	0.09284
71	S	26	0.04926
71	K	11	0.02084
71	P	10	0.01895
71	Q	10	0.01895
71	R	10	0.01895
71	N	6	0.01137

71	D	5	0.00947
71	W	4	0.00758
71	H	2	0.00379
71	C	1	0.00189
72	K	45749	86.48368
72	V	1999	3.7789
72	R	1669	3.15507
72	E	998	1.88661
72	Q	907	1.71459
72	N	384	0.72591
72	T	291	0.5501
72	M	284	0.53687
72	A	192	0.36296
72	L	139	0.26276
72	S	124	0.23441
72	W	66	0.12477
72	I	27	0.05104
72	D	25	0.04726
72	G	23	0.04348
72	Y	11	0.02079
72	H	5	0.00945
72	P	4	0.00756
72	C	1	0.00189
72	F	1	0.00189
73	NA	0	0
74	G	51170	96.06503
74	D	1514	2.84234
74	A	227	0.42616
74	S	131	0.24594
74	E	79	0.14831
74	V	46	0.08636
74	N	26	0.04881
74	C	21	0.03942
74	R	17	0.03192
74	T	12	0.02253
74	F	8	0.01502
74	H	6	0.01126
74	L	3	0.00563
74	W	3	0.00563
74	Y	2	0.00375
74	P	1	0.00188
75	R	52841	98.95504
75	Q	448	0.83897
75	L	43	0.08053
75	G	31	0.05805
75	S	9	0.01685

75	P	8	0.01498
75	C	7	0.01311
75	H	5	0.00936
75	W	3	0.00562
75	K	2	0.00375
75	N	1	0.00187
75	V	1	0.00187
76	F	50904	95.26697
76	L	886	1.65815
76	S	605	1.13226
76	V	289	0.54086
76	A	163	0.30505
76	Y	150	0.28073
76	I	141	0.26388
76	C	136	0.25452
76	T	121	0.22645
76	G	15	0.02807
76	M	6	0.01123
76	W	6	0.01123
76	P	4	0.00749
76	H	3	0.00561
76	N	2	0.00374
76	D	1	0.00187
76	E	1	0.00187
77	T	47704	89.19136
77	I	2029	3.79359
77	A	2010	3.75806
77	S	904	1.69019
77	V	359	0.67122
77	F	218	0.40759
77	N	50	0.09348
77	L	48	0.08974
77	P	37	0.06918
77	D	32	0.05983
77	R	27	0.05048
77	Y	26	0.04861
77	G	13	0.02431
77	H	12	0.02244
77	K	11	0.02057
77	C	2	0.00374
77	M	2	0.00374
77	E	1	0.00187
78	I	44969	84.04478
78	V	3329	6.22173
78	A	1161	2.16985
78	M	1093	2.04276

78	S	840	1.56992
78	T	800	1.49516
78	L	497	0.92887
78	F	235	0.4392
78	C	214	0.39996
78	G	202	0.37753
78	N	110	0.20558
78	H	18	0.03364
78	D	11	0.02056
78	K	11	0.02056
78	P	8	0.01495
78	W	4	0.00748
78	E	2	0.00374
78	R	1	0.00187
78	Y	1	0.00187
79	S	51339	95.92847
79	T	691	1.29115
79	A	607	1.1342
79	F	291	0.54374
79	P	238	0.44471
79	Y	85	0.15883
79	V	45	0.08408
79	N	43	0.08035
79	W	33	0.06166
79	D	29	0.05419
79	G	25	0.04671
79	L	25	0.04671
79	R	22	0.04111
79	H	15	0.02803
79	I	14	0.02616
79	C	10	0.01869
79	E	3	0.00561
79	Q	2	0.00374
79	M	1	0.00187
80	R	45497	85.00458
80	K	2781	5.1959
80	Q	2375	4.43734
80	S	842	1.57316
80	G	385	0.71932
80	A	271	0.50632
80	T	238	0.44467
80	H	235	0.43906
80	L	229	0.42785
80	E	165	0.30828
80	V	150	0.28025
80	I	138	0.25783

80	P	59	0.11023
80	M	58	0.10836
80	N	42	0.07847
80	F	29	0.05418
80	Y	18	0.03363
80	W	8	0.01495
80	D	2	0.00374
80	C	1	0.00187
81	D	51540	96.28965
81	G	456	0.85192
81	N	449	0.83884
81	E	321	0.59971
81	A	226	0.42222
81	V	171	0.31947
81	S	139	0.25969
81	H	111	0.20738
81	I	57	0.10649
81	Y	21	0.03923
81	T	20	0.03737
81	Q	7	0.01308
81	L	3	0.0056
81	F	2	0.00374
81	P	2	0.00374
81	R	1	0.00187
82	N	46438	86.74325
82	D	2569	4.79873
82	S	1712	3.19791
82	K	640	1.19548
82	T	389	0.72663
82	I	341	0.63697
82	Y	306	0.57159
82	V	279	0.52115
82	G	230	0.42963
82	H	163	0.30447
82	R	125	0.23349
82	A	112	0.20921
82	E	59	0.11021
82	P	53	0.099
82	F	49	0.09153
82	L	37	0.06911
82	Q	21	0.03923
82	M	9	0.01681
82	C	2	0.00374
82	W	1	0.00187
83	A	45537	85.05706
83	T	2132	3.98229

83	S	1529	2.85597
83	V	1168	2.18167
83	D	1142	2.1331
83	G	878	1.63999
83	R	323	0.60332
83	P	242	0.45202
83	N	208	0.38852
83	I	80	0.14943
83	F	77	0.14383
83	E	71	0.13262
83	L	65	0.12141
83	Y	60	0.11207
83	H	11	0.02055
83	K	8	0.01494
83	M	4	0.00747
83	C	1	0.00187
83	W	1	0.00187
84	K	44533	83.17085
84	R	2354	4.39638
84	E	2257	4.21522
84	Q	1455	2.71739
84	N	1129	2.10855
84	T	476	0.88899
84	A	287	0.53601
84	M	276	0.51546
84	D	220	0.41088
84	G	195	0.36419
84	S	120	0.22411
84	L	105	0.1961
84	V	66	0.12326
84	P	29	0.05416
84	I	23	0.04296
84	H	9	0.01681
84	W	6	0.01121
84	Y	3	0.0056
84	F	1	0.00187
85	N	45754	85.45122
85	S	2416	4.51218
85	D	1506	2.81264
85	K	1204	2.24862
85	T	645	1.20462
85	R	513	0.95809
85	A	368	0.68729
85	G	225	0.42022
85	H	206	0.38473
85	E	154	0.28761

85	I	130	0.24279
85	Y	113	0.21104
85	Q	91	0.16995
85	V	83	0.15501
85	M	51	0.09525
85	L	41	0.07657
85	P	38	0.07097
85	F	4	0.00747
85	C	2	0.00374
86	T	44348	82.81915
86	M	4868	9.09091
86	A	1112	2.07664
86	I	1000	1.86748
86	S	922	1.72182
86	V	396	0.73952
86	L	306	0.57145
86	R	212	0.39591
86	K	115	0.21476
86	E	90	0.16807
86	W	54	0.10084
86	Q	40	0.0747
86	P	39	0.07283
86	N	21	0.03922
86	G	11	0.02054
86	F	9	0.01681
86	D	2	0.00373
86	H	2	0.00373
86	Y	1	0.00187
87	V	26682	49.82633
87	L	22096	41.26237
87	M	1596	2.98039
87	I	1113	2.07843
87	A	1027	1.91783
87	G	173	0.32306
87	F	159	0.29692
87	T	151	0.28198
87	H	129	0.2409
87	Y	121	0.22596
87	P	87	0.16246
87	Q	52	0.09711
87	W	52	0.09711
87	E	43	0.0803
87	S	38	0.07096
87	C	12	0.02241
87	R	8	0.01494
87	K	7	0.01307

87	N	3	0.0056
87	D	1	0.00187
88	Y	44091	82.33306
88	F	1975	3.688
88	S	1955	3.65066
88	N	1318	2.46116
88	T	855	1.59658
88	H	822	1.53496
88	D	776	1.44906
88	W	446	0.83284
88	I	390	0.72826
88	A	365	0.68158
88	L	147	0.2745
88	C	124	0.23155
88	V	108	0.20167
88	R	75	0.14005
88	K	43	0.0803
88	E	33	0.06162
88	Q	10	0.01867
88	M	9	0.01681
88	P	7	0.01307
88	G	3	0.0056
89	L	52958	98.8908
89	P	215	0.40148
89	Q	134	0.25022
89	F	70	0.13071
89	V	45	0.08403
89	M	42	0.07843
89	I	36	0.06722
89	S	24	0.04482
89	R	13	0.02428
89	H	11	0.02054
89	A	2	0.00373
89	T	1	0.00187
89	Y	1	0.00187
90	Q	45808	85.53929
90	E	2789	5.20802
90	H	2757	5.14827
90	R	1034	1.93083
90	L	326	0.60875
90	M	236	0.44069
90	D	208	0.38841
90	K	62	0.11578
90	V	60	0.11204
90	T	56	0.10457
90	S	50	0.09337

90	Y	45	0.08403
90	I	42	0.07843
90	G	31	0.05789
90	A	20	0.03735
90	P	11	0.02054
90	N	7	0.01307
90	F	6	0.0112
90	W	4	0.00747
91	M	38540	71.96743
91	L	12717	23.74701
91	I	1138	2.12504
91	V	434	0.81043
91	T	291	0.5434
91	A	207	0.38654
91	S	74	0.13818
91	F	67	0.12511
91	K	51	0.09523
91	C	13	0.02428
91	R	7	0.01307
91	Q	5	0.00934
91	P	3	0.0056
91	W	3	0.0056
91	E	1	0.00187
91	G	1	0.00187
92	N	41911	78.26225
92	D	4062	7.58515
92	T	3035	5.66739
92	S	2126	3.96997
92	H	445	0.83097
92	I	413	0.77121
92	Y	298	0.55647
92	R	277	0.51725
92	A	217	0.40521
92	K	177	0.33052
92	G	164	0.30624
92	V	105	0.19607
92	M	89	0.16619
92	E	82	0.15312
92	L	62	0.11578
92	F	60	0.11204
92	Q	15	0.02801
92	C	8	0.01494
92	P	3	0.0056
92	W	3	0.0056
93	S	42262	78.91769
93	N	5678	10.60278

93	G	1584	2.95787
93	D	1274	2.379
93	T	919	1.71609
93	R	737	1.37623
93	A	333	0.62183
93	K	168	0.31371
93	I	118	0.22035
93	M	94	0.17553
93	Y	69	0.12885
93	C	60	0.11204
93	H	60	0.11204
93	V	51	0.09523
93	E	41	0.07656
93	Q	34	0.06349
93	F	28	0.05229
93	L	22	0.04108
93	P	13	0.02428
93	W	7	0.01307
94	L	52477	97.99261
94	V	400	0.74694
94	P	352	0.65731
94	M	121	0.22595
94	Q	93	0.17366
94	A	46	0.0859
94	S	20	0.03735
94	R	17	0.03174
94	I	14	0.02614
94	F	5	0.00934
94	T	4	0.00747
94	C	2	0.00373
94	H	1	0.00187
95	K	41035	76.62646
95	T	2899	5.41343
95	Q	2656	4.95967
95	R	2105	3.93076
95	E	1864	3.48073
95	N	1652	3.08485
95	I	334	0.62369
95	S	319	0.59568
95	A	210	0.39214
95	L	116	0.21661
95	V	99	0.18487
95	M	91	0.16993
95	G	70	0.13071
95	D	54	0.10084
95	H	28	0.05229

95	Y	13	0.02428
95	W	3	0.0056
95	F	2	0.00373
95	C	1	0.00187
95	P	1	0.00187
96	P	21755	40.62407
96	S	12685	23.68726
96	T	11954	22.32223
96	I	1757	3.28092
96	R	1496	2.79355
96	A	1389	2.59374
96	F	946	1.76651
96	L	536	1.0009
96	V	463	0.86458
96	D	144	0.2689
96	H	136	0.25396
96	Y	93	0.17366
96	N	72	0.13445
96	E	51	0.09523
96	G	29	0.05415
96	M	25	0.04668
96	W	8	0.01494
96	Q	7	0.01307
96	C	4	0.00747
96	K	2	0.00373
97	E	47466	88.63535
97	D	4249	7.93434
97	G	854	1.59471
97	A	266	0.49671
97	V	133	0.24836
97	T	127	0.23715
97	Q	125	0.23342
97	K	114	0.21288
97	S	95	0.1774
97	R	41	0.07656
97	N	39	0.07283
97	M	19	0.03548
97	H	12	0.02241
97	L	7	0.01307
97	P	3	0.0056
97	I	1	0.00187
97	Y	1	0.00187
98	D	52992	98.95429
98	G	200	0.37347
98	E	179	0.33425
98	N	71	0.13258

98	V	65	0.12138
98	H	26	0.04855
98	Y	10	0.01867
98	A	8	0.01494
98	C	1	0.00187
99	T	48496	90.5604
99	S	3007	5.61521
99	A	1131	2.11201
99	M	648	1.21006
99	N	63	0.11764
99	G	46	0.0859
99	K	24	0.04482
99	L	23	0.04295
99	I	22	0.04108
99	P	22	0.04108
99	R	21	0.03921
99	E	18	0.03361
99	D	13	0.02428
99	V	13	0.02428
99	Q	3	0.0056
99	Y	1	0.00187
100	A	46676	87.16177
100	G	6226	11.6263
100	S	272	0.50793
100	T	91	0.16993
100	V	89	0.1662
100	D	83	0.15499
100	F	35	0.06536
100	R	30	0.05602
100	Y	23	0.04295
100	P	15	0.02801
100	N	8	0.01494
100	C	2	0.00373
100	E	1	0.00187
101	V	17349	32.39715
101	M	17048	31.83507
101	L	12109	22.61209
101	T	2047	3.82252
101	I	1447	2.7021
101	R	1378	2.57325
101	K	890	1.66197
101	A	388	0.72454
101	Q	265	0.49486
101	E	204	0.38095
101	H	134	0.25023
101	F	75	0.14005

101	P	55	0.10271
101	D	48	0.08963
101	S	40	0.0747
101	N	36	0.06723
101	G	18	0.03361
101	Y	10	0.01867
101	W	9	0.01681
101	C	1	0.00187
102	Y	52905	98.79552
102	H	294	0.54902
102	C	169	0.31559
102	F	114	0.21289
102	N	49	0.0915
102	S	11	0.02054
102	D	5	0.00934
102	L	1	0.00187
102	R	1	0.00187
102	W	1	0.00187
103	Y	48247	90.22853
103	F	2622	4.9035
103	S	879	1.64385
103	H	532	0.99491
103	R	239	0.44696
103	W	195	0.36468
103	I	192	0.35907
103	T	143	0.26743
103	L	139	0.25995
103	C	126	0.23564
103	N	63	0.11782
103	V	59	0.11034
103	D	12	0.02244
103	A	11	0.02057
103	Q	8	0.01496
103	G	2	0.00374
103	P	2	0.00374
103	K	1	0.00187
104	C	53030	99.6842
104	R	82	0.15414
104	F	33	0.06203
104	S	21	0.03948
104	Y	15	0.0282
104	W	14	0.02632
104	G	3	0.00564
105	A	852	88.93528
105	T	54	5.63674
105	V	40	4.17537

105	S	4	0.41754
105	G	2	0.20877
105	L	2	0.20877
105	P	2	0.20877
105	I	1	0.10438
105	K	1	0.10438
106	A	106	40.30418
106	R	90	34.22053
106	K	26	9.88593
106	G	20	7.60456
106	L	5	1.90114
106	S	5	1.90114
106	T	5	1.90114
106	Q	2	0.76046
106	V	2	0.76046
106	E	1	0.38023
106	I	1	0.38023

Table S8: 3-VHH

The positions of amino acids	Amino acids	Counts	Percentage(%)
1	H	30537	67.19403
1	D	9150	20.13379
1	Q	4063	8.94028
1	E	779	1.71412
1	S	389	0.85596
1	M	171	0.37627
1	L	88	0.19364
1	P	57	0.12542
1	Y	57	0.12542
1	A	53	0.11662
1	N	26	0.05721
1	R	18	0.03961
1	W	17	0.03741
1	G	15	0.03301
1	V	11	0.0242
1	C	10	0.022
1	K	3	0.0066
1	F	1	0.0022
1	I	1	0.0022
2	V	44956	98.47758
2	C	287	0.62868
2	M	103	0.22562
2	L	95	0.2081
2	W	62	0.13581
2	G	53	0.1161
2	A	44	0.09638
2	E	14	0.03067
2	Q	8	0.01752
2	S	8	0.01752
2	H	7	0.01533
2	R	7	0.01533
2	D	4	0.00876
2	I	2	0.00438
2	Y	1	0.00219
3	Q	48048	98.9049
3	A	242	0.49815
3	P	65	0.1338
3	C	41	0.0844
3	S	40	0.08234
3	L	28	0.05764
3	V	27	0.05558
3	E	24	0.0494
3	K	24	0.0494

3	R	24	0.0494
3	H	8	0.01647
3	G	4	0.00823
3	W	3	0.00618
3	M	1	0.00206
3	T	1	0.00206
4	L	48497	99.13938
4	A	183	0.3741
4	P	49	0.10017
4	W	38	0.07768
4	Q	37	0.07564
4	S	35	0.07155
4	V	34	0.0695
4	M	31	0.06337
4	R	10	0.02044
4	C	1	0.00204
4	F	1	0.00204
4	I	1	0.00204
4	T	1	0.00204
5	V	49036	99.39797
5	G	139	0.28176
5	W	50	0.10135
5	A	34	0.06892
5	L	27	0.05473
5	E	19	0.03851
5	M	19	0.03851
5	F	5	0.01014
5	C	3	0.00608
5	S	1	0.00203
6	E	49163	99.49003
6	G	123	0.24891
6	A	31	0.06273
6	V	28	0.05666
6	W	15	0.03036
6	S	14	0.02833
6	M	12	0.02428
6	D	10	0.02024
6	K	7	0.01417
6	Q	4	0.00809
6	Y	4	0.00809
6	C	1	0.00202
6	F	1	0.00202
6	I	1	0.00202
6	R	1	0.00202
7	S	49292	99.51546
7	V	89	0.17968

7	F	44	0.08883
7	A	24	0.04845
7	Y	24	0.04845
7	P	21	0.0424
7	L	14	0.02826
7	T	13	0.02625
7	C	5	0.01009
7	G	3	0.00606
7	D	1	0.00202
7	I	1	0.00202
7	R	1	0.00202
8	G	49396	99.61683
8	W	100	0.20167
8	R	26	0.05243
8	E	23	0.04638
8	L	15	0.03025
8	H	7	0.01412
8	V	7	0.01412
8	D	3	0.00605
8	P	3	0.00605
8	S	3	0.00605
8	A	1	0.00202
8	F	1	0.00202
8	M	1	0.00202
9	G	49331	99.33749
9	E	88	0.1772
9	A	85	0.17116
9	R	76	0.15304
9	M	33	0.06645
9	V	32	0.06444
9	D	7	0.0141
9	C	3	0.00604
9	P	3	0.00604
9	L	1	0.00201
9	T	1	0.00201
10	NA	0	0
11	G	46439	93.40856
11	D	1602	3.2223
11	A	587	1.18071
11	E	446	0.8971
11	N	161	0.32384
11	S	129	0.25947
11	R	118	0.23735
11	V	114	0.2293
11	T	42	0.08448
11	C	27	0.05431

11	K	18	0.03621
11	Q	13	0.02615
11	H	9	0.0181
11	W	8	0.01609
11	F	1	0.00201
11	I	1	0.00201
11	P	1	0.00201
12	S	46529	93.5576
12	L	1498	3.01208
12	T	608	1.22253
12	A	526	1.05765
12	P	338	0.67963
12	E	52	0.10456
12	Q	51	0.10255
12	W	36	0.07239
12	V	29	0.05831
12	D	18	0.03619
12	R	11	0.02212
12	H	10	0.02011
12	F	8	0.01609
12	K	8	0.01609
12	M	5	0.01005
12	Y	3	0.00603
12	C	1	0.00201
12	G	1	0.00201
12	I	1	0.00201
13	V	47803	96.07098
13	A	1555	3.12513
13	M	106	0.21303
13	E	92	0.18489
13	G	61	0.12259
13	L	51	0.1025
13	I	31	0.0623
13	T	31	0.0623
13	C	8	0.01608
13	S	7	0.01407
13	P	6	0.01206
13	R	4	0.00804
13	D	2	0.00402
13	Q	1	0.00201
14	Q	45994	92.40382
14	E	1920	3.85736
14	R	607	1.21949
14	L	395	0.79357
14	H	229	0.46007
14	P	214	0.42993

14	K	152	0.30537
14	T	97	0.19488
14	A	69	0.13862
14	D	17	0.03415
14	Y	17	0.03415
14	N	14	0.02813
14	G	11	0.0221
14	S	10	0.02009
14	V	9	0.01808
14	C	8	0.01607
14	M	8	0.01607
14	W	3	0.00603
14	F	1	0.00201
15	A	39087	78.50214
15	P	4883	9.80699
15	T	3021	6.06736
15	V	1124	2.25744
15	S	743	1.49224
15	D	320	0.64269
15	E	162	0.32536
15	G	152	0.30528
15	I	105	0.21088
15	N	68	0.13657
15	R	50	0.10042
15	L	43	0.08636
15	H	12	0.0241
15	Y	7	0.01406
15	F	6	0.01205
15	Q	4	0.00803
15	C	3	0.00603
15	K	1	0.00201
16	G	49371	99.10671
16	E	232	0.46571
16	R	120	0.24089
16	D	26	0.05219
16	A	24	0.04818
16	V	23	0.04617
16	W	13	0.0261
16	S	3	0.00602
16	H	1	0.00201
16	K	1	0.00201
16	N	1	0.00201
16	T	1	0.00201
17	G	47092	94.49394
17	E	1714	3.43928
17	R	384	0.77053

17	D	147	0.29497
17	A	110	0.22072
17	T	104	0.20868
17	Q	88	0.17658
17	V	56	0.11237
17	W	32	0.06421
17	S	31	0.0622
17	K	27	0.05418
17	N	22	0.04414
17	Y	20	0.04013
17	M	7	0.01405
17	H	1	0.00201
17	L	1	0.00201
18	S	48704	97.65213
18	T	398	0.79799
18	A	317	0.63559
18	P	244	0.48922
18	F	105	0.21053
18	Y	41	0.08221
18	N	15	0.03008
18	D	9	0.01805
18	C	6	0.01203
18	L	6	0.01203
18	H	5	0.01003
18	M	5	0.01003
18	V	5	0.01003
18	R	4	0.00802
18	Q	3	0.00602
18	W	3	0.00602
18	E	2	0.00401
18	G	2	0.00401
18	I	1	0.00201
19	L	48243	96.67936
19	V	686	1.37475
19	M	272	0.54509
19	R	246	0.49299
19	P	209	0.41884
19	Q	139	0.27856
19	T	39	0.07816
19	A	26	0.0521
19	H	19	0.03808
19	S	7	0.01403
19	W	5	0.01002
19	I	4	0.00802
19	F	2	0.00401
19	C	1	0.002

19	E	1	0.002
19	G	1	0.002
20	R	40880	81.88774
20	T	4666	9.34658
20	K	2546	5.09996
20	S	457	0.91543
20	N	358	0.71712
20	G	303	0.60695
20	I	232	0.46472
20	Q	138	0.27643
20	E	109	0.21834
20	L	86	0.17227
20	V	66	0.13221
20	A	47	0.09415
20	M	17	0.03405
20	D	7	0.01402
20	P	3	0.00601
20	W	3	0.00601
20	C	2	0.00401
20	H	2	0.00401
21	L	48867	97.78289
21	V	470	0.94047
21	I	212	0.42421
21	P	211	0.42221
21	H	91	0.18209
21	F	62	0.12406
21	G	34	0.06803
21	R	9	0.01801
21	A	7	0.01401
21	S	6	0.01201
21	M	3	0.006
21	Q	2	0.004
21	T	1	0.002
22	S	47220	94.40601
22	A	1091	2.18121
22	T	1091	2.18121
22	P	164	0.32788
22	F	96	0.19193
22	D	76	0.15195
22	N	61	0.12196
22	G	57	0.11396
22	V	41	0.08197
22	I	35	0.06997
22	L	22	0.04398
22	R	16	0.03199
22	Y	12	0.02399

22	H	9	0.01799
22	Q	9	0.01799
22	C	8	0.01599
22	E	7	0.01399
22	K	2	0.004
22	M	1	0.002
23	C	49624	99.16272
23	R	202	0.40365
23	S	75	0.14987
23	Y	68	0.13588
23	W	27	0.05395
23	G	17	0.03397
23	F	14	0.02798
23	L	12	0.02398
23	A	2	0.004
23	H	1	0.002
23	M	1	0.002
24	A	26232	52.39484
24	V	8410	16.79783
24	T	5474	10.93357
24	E	2951	5.89422
24	G	2129	4.25239
24	S	1451	2.89817
24	K	828	1.65382
24	R	604	1.20641
24	L	567	1.13251
24	D	531	1.0606
24	Q	483	0.96473
24	I	299	0.59721
24	P	41	0.08189
24	H	18	0.03595
24	N	17	0.03396
24	Y	11	0.02197
24	C	9	0.01798
24	M	6	0.01198
24	F	5	0.00999
25	A	33165	66.23198
25	V	7064	14.10712
25	T	1503	3.00156
25	F	1423	2.84179
25	G	1324	2.64409
25	I	1322	2.64009
25	S	1070	2.13684
25	P	930	1.85725
25	Y	703	1.40392
25	L	429	0.85673

25	H	260	0.51923
25	R	192	0.38343
25	D	175	0.34948
25	M	146	0.29157
25	E	88	0.17574
25	W	88	0.17574
25	N	84	0.16775
25	Q	72	0.14379
25	K	21	0.04194
25	C	15	0.02996
26	S	43043	85.93132
26	P	1806	3.60551
26	G	1166	2.32781
26	R	905	1.80675
26	T	868	1.73288
26	D	648	1.29367
26	A	471	0.94031
26	Y	234	0.46716
26	F	197	0.39329
26	N	172	0.34338
26	I	157	0.31344
26	V	149	0.29746
26	L	93	0.18567
26	H	84	0.1677
26	K	40	0.07986
26	C	17	0.03394
26	E	16	0.03194
26	Q	15	0.02995
26	W	7	0.01397
26	M	2	0.00399
27	G	38953	77.73188
27	R	3013	6.01253
27	E	1879	3.7496
27	A	1673	3.33852
27	T	1079	2.15318
27	V	967	1.92968
27	L	522	1.04167
27	D	425	0.8481
27	K	404	0.80619
27	P	293	0.58469
27	S	278	0.55476
27	Q	248	0.49489
27	I	153	0.30532
27	N	97	0.19357
27	F	36	0.07184
27	H	36	0.07184

27	Y	26	0.05188
27	M	14	0.02794
27	W	14	0.02794
27	C	2	0.00399
28	Y	23665	47.19879
28	S	5122	10.2156
28	F	4800	9.57339
28	D	3044	6.07112
28	N	1865	3.71966
28	L	1855	3.69971
28	H	1742	3.47434
28	R	1516	3.02359
28	I	1302	2.59678
28	A	1217	2.42725
28	G	1112	2.21783
28	V	931	1.85684
28	P	583	1.16277
28	T	433	0.8636
28	W	319	0.63623
28	E	201	0.40089
28	Q	141	0.28122
28	K	134	0.26726
28	C	107	0.21341
28	M	50	0.09972
29	T	28999	57.79456
29	I	6130	12.217
29	S	4026	8.02376
29	A	2649	5.27942
29	P	1490	2.96955
29	R	1358	2.70647
29	L	963	1.91924
29	D	763	1.52065
29	V	721	1.43694
29	G	590	1.17586
29	N	571	1.13799
29	M	566	1.12803
29	K	348	0.69356
29	F	322	0.64174
29	Y	224	0.44643
29	H	157	0.3129
29	E	129	0.2571
29	Q	109	0.21724
29	W	51	0.10164
29	C	10	0.01993
30	Y	22254	44.32626
30	F	5552	11.05866

30	V	3215	6.40374
30	S	3151	6.27627
30	D	2499	4.97759
30	A	2382	4.74455
30	G	1602	3.19092
30	I	1364	2.71686
30	L	1260	2.50971
30	E	1097	2.18504
30	R	1095	2.18106
30	P	903	1.79863
30	T	868	1.72891
30	N	854	1.70103
30	H	799	1.59147
30	Q	577	1.14929
30	W	335	0.66726
30	K	193	0.38442
30	C	109	0.21711
30	M	96	0.19122
31	S	20489	40.78712
31	N	6872	13.67998
31	G	4907	9.76828
31	R	4174	8.30911
31	D	3683	7.33169
31	T	2412	4.80153
31	K	1851	3.68476
31	A	1560	3.10547
31	C	645	1.28399
31	I	586	1.16654
31	V	483	0.9615
31	E	440	0.8759
31	L	435	0.86595
31	F	326	0.64896
31	P	323	0.64299
31	M	310	0.61711
31	H	255	0.50762
31	Y	243	0.48374
31	Q	128	0.25481
31	W	112	0.22296
32	R	10387	20.66242
32	S	10341	20.57092
32	T	7600	15.11836
32	D	3913	7.78397
32	N	3684	7.32843
32	G	3304	6.57251
32	I	1803	3.58663
32	A	1574	3.13109

32	K	1574	3.13109
32	P	1163	2.31351
32	Y	822	1.63517
32	M	684	1.36065
32	L	659	1.31092
32	E	618	1.22936
32	V	615	1.22339
32	H	559	1.112
32	W	304	0.60473
32	Q	302	0.60076
32	F	267	0.53113
32	C	97	0.19296
33	Y	15181	30.17671
33	N	11630	23.11806
33	A	3305	6.56966
33	S	2560	5.08876
33	H	2524	5.01719
33	G	2182	4.33737
33	F	1725	3.42895
33	C	1511	3.00356
33	D	1279	2.54239
33	V	1229	2.443
33	R	1228	2.44101
33	L	1193	2.37144
33	W	1092	2.17067
33	T	897	1.78305
33	K	865	1.71944
33	I	741	1.47296
33	P	498	0.98992
33	M	420	0.83487
33	Q	125	0.24847
33	E	122	0.24251
34	C	37423	74.25788
34	Y	3031	6.01437
34	S	1857	3.68482
34	D	1648	3.2701
34	R	1315	2.60933
34	A	1002	1.98825
34	G	956	1.89698
34	F	680	1.34931
34	V	594	1.17866
34	T	342	0.67863
34	W	289	0.57346
34	L	252	0.50004
34	N	233	0.46234
34	H	220	0.43654

34	E	211	0.41868
34	I	122	0.24208
34	P	106	0.21033
34	Q	55	0.10914
34	K	44	0.08731
34	M	16	0.03175
35	NA	0	0
36	NA	0	0
37	NA	0	0
38	NA	0	0
39	M	39336	77.8191
39	V	4172	8.25354
39	L	3324	6.57593
39	I	2194	4.34043
39	F	308	0.60932
39	T	266	0.52623
39	W	262	0.51832
39	A	213	0.42138
39	K	135	0.26707
39	R	133	0.26312
39	G	51	0.10089
39	S	51	0.10089
39	Y	39	0.07715
39	Q	21	0.04154
39	E	20	0.03957
39	C	7	0.01385
39	P	7	0.01385
39	H	5	0.00989
39	N	3	0.00593
39	D	1	0.00198
40	G	31644	61.96201
40	A	16196	31.71333
40	S	1475	2.88819
40	T	422	0.82632
40	D	290	0.56785
40	V	226	0.44253
40	Y	146	0.28588
40	R	117	0.2291
40	E	102	0.19973
40	L	96	0.18798
40	N	96	0.18798
40	H	70	0.13707
40	K	50	0.0979
40	M	43	0.0842
40	C	25	0.04895
40	Q	24	0.04699

40	F	23	0.04504
40	I	12	0.0235
40	W	9	0.01762
40	P	4	0.00783
41	W	50682	98.63574
41	Y	360	0.70062
41	R	209	0.40675
41	F	47	0.09147
41	C	27	0.05255
41	L	20	0.03892
41	G	14	0.02725
41	S	9	0.01752
41	V	7	0.01362
41	I	3	0.00584
41	Q	2	0.00389
41	A	1	0.00195
41	H	1	0.00195
41	P	1	0.00195
42	F	42053	81.36561
42	Y	3554	6.8764
42	L	2384	4.61265
42	V	1579	3.0551
42	I	1186	2.29471
42	S	345	0.66752
42	W	230	0.44501
42	H	116	0.22444
42	A	61	0.11802
42	R	42	0.08126
42	N	32	0.06191
42	M	27	0.05224
42	C	26	0.05031
42	D	23	0.0445
42	T	12	0.02322
42	P	6	0.01161
42	Q	5	0.00967
42	G	2	0.00387
42	E	1	0.00193
43	R	51287	98.67439
43	H	430	0.8273
43	C	121	0.2328
43	S	43	0.08273
43	L	30	0.05772
43	Q	14	0.02694
43	P	11	0.02116
43	W	11	0.02116
43	K	10	0.01924

43	G	8	0.01539
43	F	3	0.00577
43	V	3	0.00577
43	I	2	0.00385
43	A	1	0.00192
43	M	1	0.00192
43	Y	1	0.00192
44	Q	49226	94.5109
44	R	1576	3.02582
44	E	485	0.93117
44	L	340	0.65278
44	H	121	0.23231
44	K	101	0.19391
44	V	57	0.10944
44	A	43	0.08256
44	T	33	0.06336
44	S	26	0.04992
44	P	24	0.04608
44	M	18	0.03456
44	D	13	0.02496
44	G	11	0.02112
44	W	8	0.01536
44	C	3	0.00576
45	A	37905	72.68038
45	V	4673	8.96017
45	S	1702	3.26347
45	R	1701	3.26156
45	T	1612	3.09091
45	G	1452	2.78412
45	P	1292	2.47733
45	I	499	0.9568
45	L	369	0.70753
45	Y	319	0.61166
45	F	231	0.44293
45	D	207	0.39691
45	N	62	0.11888
45	K	29	0.05561
45	H	27	0.05177
45	E	25	0.04794
45	Q	17	0.0326
45	M	15	0.02876
45	C	14	0.02684
45	W	2	0.00383
46	P	47534	90.96546
46	S	1942	3.71639
46	A	1139	2.1797

46	T	485	0.92814
46	L	471	0.90135
46	Q	232	0.44398
46	E	120	0.22964
46	V	75	0.14353
46	R	67	0.12822
46	I	59	0.11291
46	G	42	0.08038
46	D	41	0.07846
46	K	20	0.03827
46	H	9	0.01722
46	M	7	0.0134
46	C	5	0.00957
46	F	4	0.00765
46	N	1	0.00191
46	W	1	0.00191
46	Y	1	0.00191
47	G	51059	97.53018
47	E	464	0.88631
47	R	197	0.3763
47	D	177	0.3381
47	A	144	0.27506
47	S	48	0.09169
47	V	43	0.08214
47	N	41	0.07832
47	T	37	0.07068
47	L	35	0.06686
47	W	30	0.0573
47	P	23	0.04393
47	K	22	0.04202
47	Q	18	0.03438
47	M	6	0.01146
47	H	4	0.00764
47	Y	4	0.00764
48	K	43979	83.89257
48	N	2101	4.00778
48	Q	1942	3.70448
48	R	1026	1.95716
48	E	888	1.69391
48	S	549	1.04725
48	L	412	0.78591
48	T	390	0.74395
48	M	367	0.70007
48	G	271	0.51695
48	A	212	0.4044
48	D	130	0.24798

48	H	62	0.11827
48	Y	23	0.04387
48	V	17	0.03243
48	F	16	0.03052
48	P	16	0.03052
48	I	15	0.02861
48	W	6	0.01145
48	C	1	0.00191
49	E	42283	80.48233
49	G	2279	4.3379
49	D	2200	4.18752
49	Q	2008	3.82207
49	A	1348	2.56581
49	K	1037	1.97385
49	R	277	0.52725
49	S	253	0.48157
49	P	233	0.4435
49	V	197	0.37497
49	N	139	0.26458
49	L	92	0.17511
49	T	68	0.12943
49	M	47	0.08946
49	H	23	0.04378
49	Y	21	0.03997
49	W	17	0.03236
49	I	8	0.01523
49	F	6	0.01142
49	C	1	0.0019
50	R	49551	94.11754
50	C	1480	2.81112
50	P	649	1.23272
50	L	349	0.66289
50	H	312	0.59262
50	S	63	0.11966
50	Y	61	0.11586
50	V	41	0.07788
50	G	40	0.07598
50	N	25	0.04749
50	A	22	0.04179
50	F	10	0.01899
50	Q	10	0.01899
50	D	7	0.0133
50	I	6	0.0114
50	T	6	0.0114
50	K	5	0.0095
50	M	5	0.0095

50	E	4	0.0076
50	W	2	0.0038
51	E	50259	95.29218
51	D	803	1.52251
51	V	399	0.75651
51	A	317	0.60104
51	Q	290	0.54985
51	G	252	0.4778
51	K	192	0.36404
51	R	70	0.13272
51	S	60	0.11376
51	L	35	0.06636
51	T	20	0.03792
51	M	14	0.02654
51	N	14	0.02654
51	H	4	0.00758
51	I	4	0.00758
51	W	3	0.00569
51	Y	3	0.00569
51	F	2	0.00379
51	P	1	0.0019
52	G	38931	73.73994
52	A	3810	7.21659
52	W	2281	4.32048
52	L	1965	3.72194
52	R	1432	2.71238
52	E	1080	2.04565
52	V	912	1.72744
52	F	765	1.449
52	T	341	0.64589
52	M	324	0.61369
52	K	272	0.5152
52	P	255	0.483
52	S	128	0.24245
52	D	92	0.17426
52	Q	89	0.16858
52	I	44	0.08334
52	Y	35	0.06629
52	H	18	0.03409
52	N	14	0.02652
52	C	7	0.01326
53	V	46072	87.15359
53	I	4123	7.79941
53	L	2247	4.25061
53	A	197	0.37266
53	D	81	0.15323

53	F	44	0.08323
53	G	38	0.07188
53	C	16	0.03027
53	T	14	0.02648
53	M	12	0.0227
53	P	8	0.01513
53	H	3	0.00568
53	S	3	0.00568
53	E	2	0.00378
53	N	1	0.00189
53	R	1	0.00189
53	W	1	0.00189
54	A	47662	90.03192
54	S	3005	5.67634
54	G	1352	2.55388
54	V	548	1.03515
54	T	230	0.43446
54	I	37	0.06989
54	L	31	0.05856
54	P	30	0.05667
54	E	14	0.02645
54	Q	10	0.01889
54	C	5	0.00944
54	D	5	0.00944
54	R	4	0.00756
54	W	2	0.00378
54	Y	2	0.00378
54	F	1	0.00189
54	K	1	0.00189
55	A	16164	30.49236
55	S	7696	14.51802
55	T	7286	13.74458
55	R	5905	11.13941
55	V	5272	9.94529
55	G	2930	5.52726
55	F	1957	3.69176
55	I	989	1.86569
55	C	795	1.49972
55	L	703	1.32616
55	Y	637	1.20166
55	H	558	1.05263
55	Q	493	0.93001
55	N	456	0.86022
55	D	356	0.67157
55	E	335	0.63196
55	M	283	0.53386

55	K	99	0.18676
55	W	69	0.13016
55	P	27	0.05093
56	I	39704	74.82004
56	L	4597	8.6628
56	V	3695	6.96303
56	M	1139	2.14638
56	F	954	1.79776
56	T	842	1.5867
56	H	514	0.96861
56	D	322	0.60679
56	A	281	0.52953
56	S	250	0.47111
56	R	249	0.46923
56	K	152	0.28644
56	Y	83	0.15641
56	N	77	0.1451
56	Q	59	0.11118
56	G	50	0.09422
56	C	39	0.07349
56	W	31	0.05842
56	P	20	0.03769
56	E	8	0.01508
57	D	17905	33.7067
57	Y	9373	17.64495
57	A	5451	10.26167
57	S	3598	6.77334
57	G	2480	4.66867
57	R	2319	4.36559
57	N	2208	4.15663
57	W	1896	3.56928
57	T	1580	2.9744
57	E	1313	2.47176
57	H	1256	2.36446
57	V	974	1.83358
57	F	657	1.23682
57	L	480	0.90361
57	M	397	0.74736
57	C	373	0.70218
57	K	278	0.52334
57	Q	262	0.49322
57	I	200	0.37651
57	P	120	0.2259
58	S	15339	28.83054
58	T	14997	28.18773
58	R	4439	8.34336

58	I	2901	5.4526
58	G	2822	5.30411
58	P	2204	4.14255
58	A	2096	3.93955
58	N	1759	3.30614
58	F	1473	2.76859
58	L	1102	2.07127
58	D	940	1.76678
58	V	786	1.47733
58	K	734	1.3796
58	Y	389	0.73115
58	M	323	0.6071
58	H	306	0.57514
58	E	248	0.46613
58	W	141	0.26502
58	Q	135	0.25374
58	C	70	0.13157
59	D	20058	37.64216
59	G	16267	30.52772
59	S	2987	5.6056
59	A	2748	5.15708
59	R	2286	4.29006
59	N	1644	3.08524
59	T	1103	2.06996
59	E	1058	1.98551
59	L	1043	1.95736
59	V	1007	1.8898
59	Y	964	1.80911
59	P	490	0.91957
59	F	419	0.78632
59	H	385	0.72252
59	I	306	0.57426
59	Q	264	0.49544
59	K	159	0.29839
59	W	46	0.08633
59	M	33	0.06193
59	C	19	0.03566
60	G	36458	68.30923
60	S	5901	11.05636
60	D	3942	7.3859
60	T	2022	3.7885
60	A	1099	2.05913
60	R	882	1.65255
60	N	524	0.98179
60	E	485	0.90872
60	L	445	0.83377

60	Y	412	0.77194
60	V	311	0.5827
60	H	226	0.42344
60	I	194	0.36349
60	K	142	0.26606
60	F	122	0.22858
60	Q	80	0.14989
60	P	66	0.12366
60	W	27	0.05059
60	C	21	0.03935
60	M	13	0.02436
61	G	16714	31.27503
61	S	8768	16.40657
61	T	5495	10.28218
61	R	4393	8.22013
61	A	3979	7.44545
61	I	3499	6.54728
61	D	3104	5.80817
61	N	1362	2.54856
61	V	1211	2.26601
61	E	920	1.72149
61	L	859	1.60735
61	K	823	1.53999
61	H	618	1.15639
61	Y	436	0.81584
61	P	415	0.77654
61	M	302	0.5651
61	F	218	0.40792
61	Q	181	0.33868
61	W	119	0.22267
61	C	26	0.04865
62	T	23933	44.72371
62	S	6331	11.83077
62	N	5161	9.64439
62	A	4306	8.04664
62	R	3391	6.33678
62	I	2712	5.06793
62	P	1928	3.60286
62	V	950	1.77527
62	E	915	1.70986
62	D	719	1.3436
62	K	685	1.28006
62	G	562	1.05021
62	L	461	0.86147
62	Q	338	0.63162
62	Y	306	0.57182

62	M	282	0.52697
62	F	264	0.49334
62	H	201	0.37561
62	W	53	0.09904
62	C	15	0.02803
63	T	18816	79.16526
63	P	1153	4.85106
63	A	1088	4.57758
63	S	617	2.59593
63	R	578	2.43184
63	K	489	2.05739
63	I	357	1.50202
63	V	163	0.6858
63	E	159	0.66897
63	G	100	0.42073
63	L	80	0.33659
63	Q	54	0.2272
63	M	50	0.21037
63	N	18	0.07573
63	D	12	0.05049
63	F	12	0.05049
63	Y	12	0.05049
63	H	6	0.02524
63	W	4	0.01683
63	NA	0	0
64	NA	0	0
65	NA	0	0
66	Y	16127	30.06749
66	T	8566	15.97062
66	S	7186	13.39772
66	A	3471	6.4714
66	N	2778	5.17936
66	R	2752	5.13088
66	D	2164	4.0346
66	W	1487	2.77239
66	F	1315	2.45171
66	I	1261	2.35103
66	H	1078	2.00984
66	K	1066	1.98747
66	V	1036	1.93154
66	G	1000	1.86442
66	L	765	1.42628
66	M	627	1.16899
66	E	548	1.0217
66	Q	259	0.48288
66	C	124	0.23119

66	P	26	0.04847
67	Y	50566	93.7762
67	V	1401	2.5982
67	I	316	0.58603
67	F	285	0.52854
67	H	266	0.49331
67	A	256	0.47476
67	L	228	0.42283
67	C	190	0.35236
67	T	127	0.23553
67	S	95	0.17618
67	D	86	0.15949
67	N	52	0.09644
67	M	11	0.0204
67	Q	11	0.0204
67	W	11	0.0204
67	P	6	0.01113
67	E	5	0.00927
67	R	5	0.00927
67	G	3	0.00556
67	K	2	0.00371
68	A	39341	72.30074
68	T	3909	7.18395
68	S	2353	4.32433
68	G	2115	3.88694
68	V	1802	3.31171
68	D	1142	2.09876
68	L	1132	2.08039
68	R	680	1.2497
68	P	396	0.72777
68	H	374	0.68734
68	I	349	0.64139
68	E	330	0.60647
68	N	144	0.26464
68	K	125	0.22972
68	Y	65	0.11946
68	M	52	0.09557
68	Q	52	0.09557
68	F	47	0.08638
68	W	4	0.00735
68	C	1	0.00184
69	D	43429	79.32962
69	E	2913	5.32103
69	N	2147	3.92182
69	G	1687	3.08156
69	Y	1413	2.58106

69	A	1228	2.24313
69	S	489	0.89323
69	P	368	0.67221
69	H	267	0.48772
69	T	199	0.3635
69	V	170	0.31053
69	Q	132	0.24112
69	R	111	0.20276
69	K	82	0.14979
69	W	33	0.06028
69	I	29	0.05297
69	F	18	0.03288
69	L	18	0.03288
69	C	6	0.01096
69	M	6	0.01096
70	S	51170	91.38316
70	A	1746	3.11814
70	F	1184	2.11447
70	P	922	1.64658
70	T	267	0.47683
70	D	149	0.2661
70	L	89	0.15894
70	Y	89	0.15894
70	V	87	0.15537
70	G	82	0.14644
70	R	72	0.12858
70	N	52	0.09287
70	H	32	0.05715
70	K	15	0.02679
70	Q	13	0.02322
70	C	11	0.01964
70	I	7	0.0125
70	W	4	0.00714
70	M	3	0.00536
70	E	1	0.00179
71	V	52005	91.47435
71	A	1757	3.09048
71	L	1421	2.49947
71	M	1278	2.24794
71	E	96	0.16886
71	T	95	0.1671
71	I	58	0.10202
71	G	52	0.09147
71	F	45	0.07915
71	Q	10	0.01759
71	S	9	0.01583

71	P	6	0.01055
71	R	6	0.01055
71	D	5	0.00879
71	K	4	0.00704
71	N	2	0.00352
71	W	2	0.00352
71	C	1	0.00176
72	K	49716	87.10339
72	E	1726	3.02399
72	Q	1564	2.74016
72	R	1390	2.43531
72	A	674	1.18086
72	T	496	0.869
72	N	381	0.66752
72	S	298	0.5221
72	L	266	0.46604
72	M	254	0.44501
72	G	131	0.22951
72	D	80	0.14016
72	V	37	0.06482
72	W	29	0.05081
72	I	18	0.03154
72	F	7	0.01226
72	P	6	0.01051
72	Y	3	0.00526
72	H	1	0.00175
73	NA	0	0
74	G	54303	94.18447
74	D	2414	4.1869
74	A	489	0.84813
74	E	124	0.21507
74	S	109	0.18905
74	V	73	0.12661
74	C	29	0.0503
74	P	22	0.03816
74	R	21	0.03642
74	N	20	0.03469
74	T	18	0.03122
74	H	15	0.02602
74	K	10	0.01734
74	L	5	0.00867
74	W	2	0.00347
74	F	1	0.00173
74	Y	1	0.00173
75	R	57633	99.51995
75	Q	139	0.24002

75	L	43	0.07425
75	G	20	0.03454
75	P	15	0.0259
75	C	11	0.01899
75	H	10	0.01727
75	W	9	0.01554
75	S	7	0.01209
75	K	6	0.01036
75	T	6	0.01036
75	D	4	0.00691
75	I	3	0.00518
75	F	2	0.00345
75	V	2	0.00345
75	N	1	0.00173
76	F	56527	97.38814
76	V	397	0.68398
76	L	336	0.57888
76	S	272	0.46862
76	A	207	0.35663
76	Y	132	0.22742
76	I	65	0.11199
76	G	38	0.06547
76	W	29	0.04996
76	C	24	0.04135
76	T	9	0.01551
76	D	2	0.00345
76	P	2	0.00345
76	H	1	0.00172
76	M	1	0.00172
76	Q	1	0.00172
77	T	51332	88.2448
77	I	2264	3.89204
77	A	2247	3.86282
77	S	1353	2.32594
77	V	245	0.42118
77	N	204	0.3507
77	D	106	0.18222
77	F	102	0.17535
77	L	67	0.11518
77	H	59	0.10143
77	R	56	0.09627
77	K	40	0.06876
77	Y	28	0.04813
77	P	27	0.04642
77	G	17	0.02922
77	M	14	0.02407

77	E	5	0.0086
77	C	4	0.00688
78	I	51209	87.86116
78	V	4167	7.14947
78	L	1603	2.75033
78	A	350	0.60051
78	T	329	0.56448
78	F	212	0.36374
78	M	203	0.34829
78	S	86	0.14755
78	N	67	0.11495
78	C	19	0.0326
78	G	13	0.0223
78	Y	9	0.01544
78	D	7	0.01201
78	R	4	0.00686
78	H	2	0.00343
78	P	2	0.00343
78	Q	1	0.00172
78	W	1	0.00172
79	S	54197	92.82533
79	T	2038	3.49056
79	A	739	1.26571
79	W	340	0.58233
79	F	302	0.51725
79	P	254	0.43504
79	Y	112	0.19183
79	V	104	0.17812
79	I	74	0.12674
79	L	52	0.08906
79	D	45	0.07707
79	R	31	0.05309
79	G	28	0.04796
79	H	27	0.04624
79	N	26	0.04453
79	E	10	0.01713
79	C	5	0.00856
79	Q	2	0.00343
80	Q	19081	32.66008
80	R	15895	27.20675
80	K	14953	25.59437
80	H	1989	3.40448
80	L	1649	2.82252
80	V	1459	2.4973
80	E	1283	2.19605
80	T	341	0.58367

80	A	316	0.54088
80	I	304	0.52034
80	P	290	0.49638
80	S	243	0.41593
80	G	205	0.35089
80	Y	109	0.18657
80	F	89	0.15234
80	N	86	0.1472
80	M	81	0.13864
80	W	23	0.03937
80	D	19	0.03252
80	C	8	0.01369
81	D	54539	93.27211
81	G	1173	2.00605
81	E	986	1.68625
81	N	789	1.34934
81	V	404	0.69092
81	A	181	0.30954
81	Q	150	0.25653
81	H	92	0.15734
81	S	45	0.07696
81	I	35	0.05986
81	T	26	0.04446
81	Y	22	0.03762
81	P	11	0.01881
81	F	9	0.01539
81	L	4	0.00684
81	K	3	0.00513
81	R	3	0.00513
81	M	1	0.00171
82	N	38917	66.51001
82	S	6249	10.67968
82	K	4706	8.04266
82	D	2392	4.08798
82	T	1306	2.23198
82	G	1185	2.02519
82	Y	722	1.23391
82	R	629	1.07497
82	I	606	1.03567
82	H	373	0.63747
82	A	314	0.53663
82	V	294	0.50245
82	P	225	0.38453
82	E	177	0.3025
82	Q	134	0.22901
82	L	115	0.19654

82	F	104	0.17774
82	M	52	0.08887
82	W	7	0.01196
82	C	6	0.01025
83	A	46334	79.13984
83	G	2844	4.85764
83	T	2162	3.69276
83	V	1867	3.18889
83	D	1648	2.81483
83	S	1313	2.24264
83	R	941	1.60726
83	N	425	0.72591
83	P	392	0.66955
83	E	186	0.31769
83	I	109	0.18618
83	F	87	0.1486
83	K	50	0.0854
83	Y	46	0.07857
83	L	44	0.07515
83	H	43	0.07345
83	W	24	0.04099
83	M	15	0.02562
83	Q	15	0.02562
83	C	2	0.00342
84	K	44364	75.75432
84	N	3532	6.03111
84	R	3339	5.70155
84	E	2823	4.82045
84	Q	914	1.56071
84	T	679	1.15944
84	G	554	0.94599
84	S	455	0.77694
84	A	449	0.7667
84	D	413	0.70522
84	M	390	0.66595
84	L	271	0.46275
84	V	178	0.30395
84	P	83	0.14173
84	H	54	0.09221
84	I	40	0.0683
84	W	14	0.02391
84	Y	8	0.01366
84	F	3	0.00512
85	N	43727	74.65895
85	D	4385	7.4869
85	K	3048	5.20412

85	S	2254	3.84845
85	R	1282	2.18887
85	T	1249	2.13253
85	H	623	1.0637
85	A	387	0.66076
85	Y	325	0.5549
85	G	297	0.50709
85	I	287	0.49002
85	M	170	0.29026
85	E	159	0.27147
85	L	135	0.2305
85	P	102	0.17415
85	Q	71	0.12122
85	F	32	0.05464
85	V	22	0.03756
85	W	8	0.01366
85	C	6	0.01024
86	T	47332	80.79753
86	I	3244	5.53763
86	M	1740	2.97025
86	S	1709	2.91733
86	L	1437	2.45301
86	A	1338	2.28402
86	V	872	1.48854
86	W	186	0.31751
86	R	176	0.30044
86	K	164	0.27995
86	N	116	0.19802
86	E	112	0.19119
86	P	47	0.08023
86	F	31	0.05292
86	G	27	0.04609
86	Q	23	0.03926
86	H	13	0.02219
86	D	7	0.01195
86	C	4	0.00683
86	Y	3	0.00512
87	L	27239	46.49484
87	V	25387	43.33362
87	M	1794	3.06222
87	I	1618	2.7618
87	A	1023	1.74618
87	T	460	0.78518
87	W	237	0.40454
87	F	235	0.40113
87	P	119	0.20312

87	G	110	0.18776
87	S	107	0.18264
87	Q	82	0.13997
87	E	51	0.08705
87	H	41	0.06998
87	Y	38	0.06486
87	R	22	0.03755
87	C	9	0.01536
87	K	8	0.01366
87	D	4	0.00683
87	N	1	0.00171
88	Y	47733	81.46675
88	N	2615	4.46307
88	F	2152	3.67286
88	S	1553	2.65053
88	D	1107	1.88934
88	H	1063	1.81424
88	T	713	1.21689
88	V	360	0.61442
88	A	245	0.41815
88	W	238	0.4062
88	L	236	0.40279
88	I	222	0.37889
88	C	133	0.22699
88	E	90	0.1536
88	R	64	0.10923
88	Q	30	0.0512
88	K	19	0.03243
88	P	9	0.01536
88	G	7	0.01195
88	M	3	0.00512
89	L	58168	99.25602
89	P	227	0.38735
89	Q	99	0.16893
89	M	30	0.05119
89	V	20	0.03413
89	S	17	0.02901
89	I	15	0.0256
89	R	15	0.0256
89	F	7	0.01194
89	H	3	0.00512
89	A	2	0.00341
89	W	1	0.00171
90	Q	49624	84.6667
90	E	2865	4.88816
90	M	2116	3.61024

90	R	1510	2.57631
90	H	857	1.46218
90	L	853	1.45536
90	D	280	0.47773
90	K	150	0.25592
90	V	91	0.15526
90	T	58	0.09896
90	S	44	0.07507
90	I	39	0.06654
90	A	29	0.04948
90	G	23	0.03924
90	P	17	0.029
90	W	16	0.0273
90	F	15	0.02559
90	N	14	0.02389
90	Y	9	0.01536
90	C	1	0.00171
91	M	55173	94.12939
91	L	1337	2.28103
91	I	1152	1.9654
91	V	650	1.10895
91	T	189	0.32245
91	K	67	0.11431
91	A	19	0.03242
91	R	14	0.02389
91	S	5	0.00853
91	F	4	0.00682
91	P	2	0.00341
91	C	1	0.00171
91	N	1	0.00171
92	N	46267	78.93507
92	D	6991	11.92718
92	S	1863	3.17842
92	T	1475	2.51646
92	H	521	0.88887
92	A	248	0.42311
92	G	213	0.36339
92	K	204	0.34804
92	R	199	0.33951
92	I	148	0.2525
92	Y	145	0.24738
92	E	140	0.23885
92	V	102	0.17402
92	F	44	0.07507
92	L	25	0.04265
92	M	13	0.02218

92	Q	12	0.02047
92	P	2	0.00341
92	C	1	0.00171
92	W	1	0.00171
93	S	37372	63.75734
93	N	12310	21.00109
93	D	2673	4.56019
93	G	2556	4.36058
93	R	985	1.68043
93	T	838	1.42964
93	A	383	0.65341
93	H	317	0.54081
93	Y	310	0.52887
93	K	293	0.49986
93	Q	145	0.24737
93	M	139	0.23714
93	I	95	0.16207
93	C	58	0.09895
93	E	38	0.06483
93	V	38	0.06483
93	L	36	0.06142
93	F	21	0.03583
93	W	6	0.01024
93	P	3	0.00512
94	L	57709	98.4476
94	V	327	0.55784
94	P	266	0.45378
94	Q	95	0.16206
94	M	93	0.15865
94	A	52	0.08871
94	T	27	0.04606
94	R	19	0.03241
94	I	17	0.029
94	S	7	0.01194
94	F	3	0.00512
94	H	3	0.00512
94	K	1	0.00171
95	K	41925	71.52118
95	Q	7172	12.23494
95	E	2636	4.49684
95	T	2154	3.67458
95	R	2140	3.65069
95	N	1244	2.12218
95	I	307	0.52372
95	S	304	0.5186
95	A	139	0.23712

95	M	139	0.23712
95	G	115	0.19618
95	D	110	0.18765
95	L	103	0.17571
95	V	79	0.13477
95	H	46	0.07847
95	F	2	0.00341
95	P	2	0.00341
95	W	1	0.00171
95	Y	1	0.00171
96	P	48892	83.40498
96	T	2192	3.73934
96	I	1993	3.39986
96	S	1522	2.59638
96	L	1242	2.11873
96	A	1083	1.84749
96	V	962	1.64108
96	H	223	0.38042
96	F	179	0.30536
96	R	157	0.26783
96	D	47	0.08018
96	E	38	0.06482
96	N	31	0.05288
96	G	18	0.03071
96	Y	17	0.029
96	Q	16	0.02729
96	M	8	0.01365
97	E	52238	89.11293
97	D	4343	7.40873
97	G	1278	2.18014
97	A	209	0.35653
97	V	122	0.20812
97	Q	101	0.1723
97	K	88	0.15012
97	R	63	0.10747
97	N	52	0.08871
97	S	40	0.06824
97	T	38	0.06482
97	H	17	0.029
97	P	14	0.02388
97	M	8	0.01365
97	L	5	0.00853
97	W	3	0.00512
97	Y	1	0.00171
98	D	58177	99.24259
98	G	221	0.377

98	V	72	0.12282
98	N	61	0.10406
98	E	39	0.06653
98	Y	23	0.03924
98	A	15	0.02559
98	H	11	0.01876
98	I	1	0.00171
98	K	1	0.00171
99	T	52068	88.82293
99	S	5464	9.32105
99	A	832	1.41931
99	G	57	0.09724
99	I	57	0.09724
99	E	34	0.058
99	N	29	0.04947
99	M	22	0.03753
99	P	21	0.03582
99	R	14	0.02388
99	D	7	0.01194
99	F	6	0.01024
99	C	5	0.00853
99	K	3	0.00512
99	V	1	0.00171
100	A	50268	85.75669
100	G	7507	12.80686
100	D	271	0.46232
100	S	267	0.4555
100	T	128	0.21837
100	V	91	0.15525
100	N	41	0.06995
100	Y	28	0.04777
100	P	7	0.01194
100	R	4	0.00682
100	E	2	0.00341
100	L	2	0.00341
100	C	1	0.00171
101	M	35575	60.69162
101	T	8985	15.32858
101	V	7391	12.60919
101	I	3360	5.73222
101	L	1356	2.31336
101	R	624	1.06456
101	K	575	0.98096
101	E	192	0.32756
101	A	161	0.27467
101	N	118	0.20131

101	S	96	0.16378
101	Q	63	0.10748
101	D	34	0.058
101	W	25	0.04265
101	F	24	0.04094
101	P	14	0.02388
101	G	12	0.02047
101	H	10	0.01706
101	C	1	0.00171
102	Y	58024	98.99848
102	H	170	0.29005
102	C	168	0.28664
102	F	164	0.27981
102	N	58	0.09896
102	S	18	0.03071
102	D	6	0.01024
102	L	1	0.00171
102	Q	1	0.00171
102	R	1	0.00171
103	Y	51952	89.02293
103	F	1918	3.28661
103	T	817	1.39998
103	I	789	1.352
103	S	736	1.26118
103	R	677	1.16008
103	H	584	1.00072
103	V	266	0.45581
103	L	188	0.32215
103	Q	152	0.26046
103	C	130	0.22276
103	N	63	0.10795
103	A	35	0.05997
103	D	22	0.0377
103	W	13	0.02228
103	P	6	0.01028
103	G	5	0.00857
103	K	5	0.00857
104	C	58234	99.79778
104	R	74	0.12682
104	S	16	0.02742
104	Y	15	0.02571
104	W	7	0.012
104	F	4	0.00685
104	G	2	0.00343
105	A	4930	94.75303
105	V	169	3.24813

105	T	35	0.67269
105	G	31	0.59581
105	S	30	0.57659
105	E	7	0.13454
105	Q	1	0.01922
106	A	3924	84.45975
106	E	303	6.52174
106	P	131	2.81963
106	T	110	2.36763
106	V	66	1.42058
106	S	55	1.18381
106	G	41	0.88248
106	R	11	0.23676
106	K	2	0.04305
106	D	1	0.02152
106	I	1	0.02152
106	L	1	0.02152
